# Synthesis, Antibacterial and Antifungal Activity of Some New Pyrazoline and Pyrazole Derivatives

**DOI:** 10.3390/molecules18032683

**Published:** 2013-02-28

**Authors:** Seham Y. Hassan

**Affiliations:** Department of Chemistry, Faculty of Science, University of Alexandria, PO Box 426, Ibrahimia 21321, Alexandria, Egypt; E-Mail: sehamyassen@yahoo.com; Tel.: +20-01-286-525-997; Fax: +20-3-5932-488

**Keywords:** chalcones, pyrazoline, pyrazole, carbothioamide, benzenesulfonamide, thiazolidine, indole, quinoxaline

## Abstract

A series of 2-pyrazolines **5**–**9** have been synthesized from *α,β*-unsaturated ketones **2**–**4**. New 2-pyrazoline derivatives **13**–**15** bearing benzenesulfonamide moieties were then synthesized by condensing the appropriate chalcones **2**–**4** with 4-hydrazinyl benzenesulfonamide hydrochloride. Ethyl [1,2,4] triazolo[3,4-*c*][1,2,4]triazino[5,6-*b*]-5*H*-indole-5-ethanoate (**26**) and 1-(5*H*-[1,2,4]triazino[5,6-*b*] indol-3-yl)-3-methyl-1*H*-pyrazol-5(4*H*)-one (**32**) were synthesized from 3-hydrazinyl-5*H*-[1,2,4]triazino[5,6-*b*]indole (**24**). On the other hand ethyl[1,2,4]triazolo[3,4-*c*][1,2,4]triazino[5,6-*b*]-5,10-dihydroquinoxaline-5-ethanoate (**27**) and 1-(5,10-dihydro-[1,2,4]triazino[5,6-*b*]quinoxalin-3-yl)-3-methyl-1*H*-pyrazol-5(4*H*)-one (**33**) were synthesized from 3-hydrazinyl-5,10-dihydro-[1,2,4]triazino[5,6-*b*]quinoxaline (**25**) by reaction with diethyl malonate or ethyl acetoacetate, respectively. Condensation of 6,6-dimethyl-4-*oxo*-4,5,6,7-tetrahydro-1*H*-indole-2-carbaldehyde (**1**') with compound **24** or **25** afforded the corresponding Schiff's bases **36** and **37**, respectively. Reaction of the Schiff's base **37** with benzoyl hydrazine or acetic anhydride afforded benzohydrazide derivative **39** and the cyclized compound **40**, respectively. Furthermore, the pyrazole derivatives **42**–**44** were synthesized by cyclization of hydrazine derivative **25** with the prepared chalcones **2**–**4**. All the newly synthesized compounds have been characterized on the basis of IR and ^1^H-NMR spectral data as well as physical data. Antimicrobial activity against the organisms *E. coli* ATCC8739 and *P. aeruginosa* ATCC 9027 as examples of Gram-negative bacteria, *S. aureus* ATCC 6583P as an example of Gram-positive bacteria and *C. albicans* ATCC 2091 as an example of a yeast-like fungus have been studied using the Nutrient Agar (NA) and Sabouraud Dextrose Agar (SDA) diffusion methods. The best performance was found for the compounds **16**, **17**, **19** and **20**.

## 1. Introduction

Chalcones have been recently the subject of great interest due to their interesting pharmacological activities, including antioxidant [1,2], antibacterial [3], antileishmanial [4], anticancer [5], antiangiogenic [6], anti-infective, anti-inflammatory [7], antifungal [8], anti-malarial [9], anti-tumor [10], anti-protozoal [11] and cytotoxic properties [12]. Many pyrazole derivatives are reported to have a broad spectrum of biological activities, such as anti-inflammatory [13], antifungal [14], antiviral [15], cytotoxic [12], A3 adenosine receptor antagonists [16], antioxidant [13], antihypertensive [17], tranquilizing, muscle relaxant, psychoanaleptic, hypnotic, ulcerogenic, antidepressant, antibacterial and analgesic effects [18]. Pharmacologically-interesting heterocyclic systems like pyrazolines have been widely studied owing to their pharmacological activities, which include anti-tumor [19,20], anti-inflammatory [21,22,23,24,25,26,27,28,29,30,31,32], anti-parasitary [33], anticonvulsant [34], antimicrobial [35,36,37,38,39], antinociceptives [40], antimalarial [41], nitric oxide synthase inhibitory, associated with diseases such as Alzheimer, Huntington, and inflammatory arthritis [42], antidepressant [43,44], anticancer [45,46,47], antibacterial [48], antitubercular, analgesic [49], antiviral [46], antioxidant [50], antiamoebic [51,52,53], cytotoxic [53], antidiabetic [20], antifungal [54,55], antinociceptive [56], antimycobacterial [57], antihepatotoxic [58] and pesticidal properties [59].

Substituted 2-pyrazolines have been synthesized from *α,β*-unsaturated ketones and hydrazine hydrate with acetic/formic acid in ethanol/dimethyl sulfoxide (DMSO) [60], hydrazine in dimethyl formamide (DMF) or acetic acid [46], nicotinic acid hydrazide in *n*-butanol [41], phenyl hydrazine hydrochloride in the presence of sodium acetate [39], hydrazine hydrate in ethanol and DMF [25], and phenyl hydrazine in the presence of hot pyridine [27]. Some new substituted 2-pyrazoline derivatives bearing benzenesulfonamide moieties [21,22,23,26] were synthesized by condensing appropriate chalcones with 4-hydrazinobenzenesulfonamide hydrochloride. In view of these observations and in continuation of our research programme on the synthesis of five-membered heterocyclic compounds [61,62,63,64,65,66], we report herein the synthesis of some new pyrazoline and pyrazole derivatives bearing an indoline and quinoxaline moiety, which have been found to possess an interesting profile of antimicrobial activity.

## 2. Results and Discussion

### 2.1. Chemistry

#### 2.1.1. Preparation of the Chalcones **2–4**

**Scheme 1:**
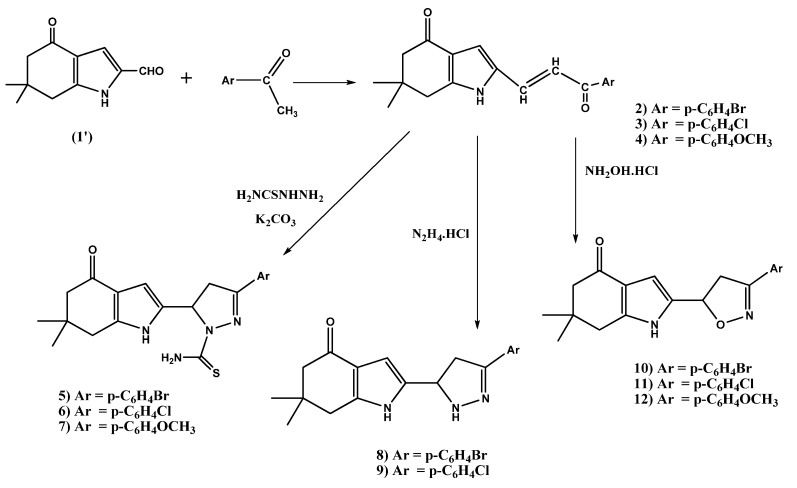
Synthesis of chalcones **2**–**4**, pyrazoline derivatives **5**–**9** and isoxazoline derivatives **10**–**12**.

The chalcones **2**–**4** were prepared as starting material to obtain the desired pyrazoline and pyrazole derivatives. The sequence leading to the title compounds is outlined in Scheme 1. The desired compounds were prepared by the reaction of 6,6-dimethyl-4-oxo-4,5,6,7-tetrahydro-1*H*-indole-2-carbaldehyde (**1**') [67] with different acetophenones (*p*-bromo-, *p*-chloro-, or *p*-methoxyacetophenones) in aqueous ethanolic KOH in good yield (Scheme 1). Their ^1^H-NMR spectra showed the -CH=CH- protons as a multiplet in the 7.52–7.63 ppm range for compound **3**, and two doublet peaks at 7.54, 7.60 and 7.48, 7.60 ppm with coupling constants of 15.3 Hz for compounds **2** and **4**, respectively. The ^13^C-NMR spectrum of prototypical compound **2** showed the two carbonyl carbons at 187.8 and 192.7 ppm.

#### 2.1.2. Synthesis of Pyrazoline Derivatives **5–9** and Isoxazoline Derivatives **10–12**

The compounds **2**–**4** were converted into the corresponding 3-(aryl)-5-(6,6-dimethyl-4-oxo-4,5,6,7-tetrahydro-1*H*-indol-2-yl)-4,5-dihydro-1*H*-pyrazole-1-carbothioamides **5**–**7** by treatment with thiosemicarbazide (Scheme 1).

Their ^1^H-NMR spectra showed multiplets within the 3.33–4.82 range corresponding to H_4_, H_4'_ of the pyrazoline ring, where a multiplet at 6.92–6.98 ppm is observed for compound **7** corresponding to H_5_. A doublet of doublets at 6.73–6.88 corresponding to H_5_ of the pyrazoline ring was observed for compounds **5** and **6**, respectively. In addition to a broad signal corresponding to the exchangeable NH_2_ protons was observed in the 7.25–8.01 ppm range. The ^13^C-NMR spectrum of compound **6** chosen as a prototype showed C=S and C=O peaks at 180.0 and 204.6 ppm, respectively. Reaction of compounds **2** and **3** with hydrazinum chloride gave rise to 2-(3-(aryl)-4,5-dihydro-1*H*-pyrazol-5-yl)-6,6-dimethyl-6,7-dihydro-1*H*-indol-4(5*H*)-ones **8** and **9** (Scheme 1). In their ^1^H-NMR spectra, the appearance of signals in the ranges 3.33–3.75 and 5.49–6.70 ppm corresponding to (H_4_, H_4'_) and H_5_ of the pyrazoline ring respectively was observed. The product of compound **4** with hydrazinium chloride could not be separated in a pure form. The 2-(3-(aryl)-4,5-dihydroisoxazol-5-yl)-6,6-dimethyl-6,7-dihydro-*1H*-indol-4(5H)-ones **10**–**12** were synthesized by cyclization of **2**–**4** in presence of hydroxylamine hydrochloride. Their ^1^H-NMR spectra showed three signals within the ranges 3.34–4.22 and 5.55–6.52 ppm corresponding to the (H_4_, H_4'_) and H_5_ of the pyrazoline ring, respectively. The ^13^C-NMR spectrum of compound **11** selected as a prototype showed the carbonyl carbon at 193.7 ppm.

#### 2.1.3. Synthesis of Benzenesulfonamide Derivatives **13–21**

**Scheme 2:**
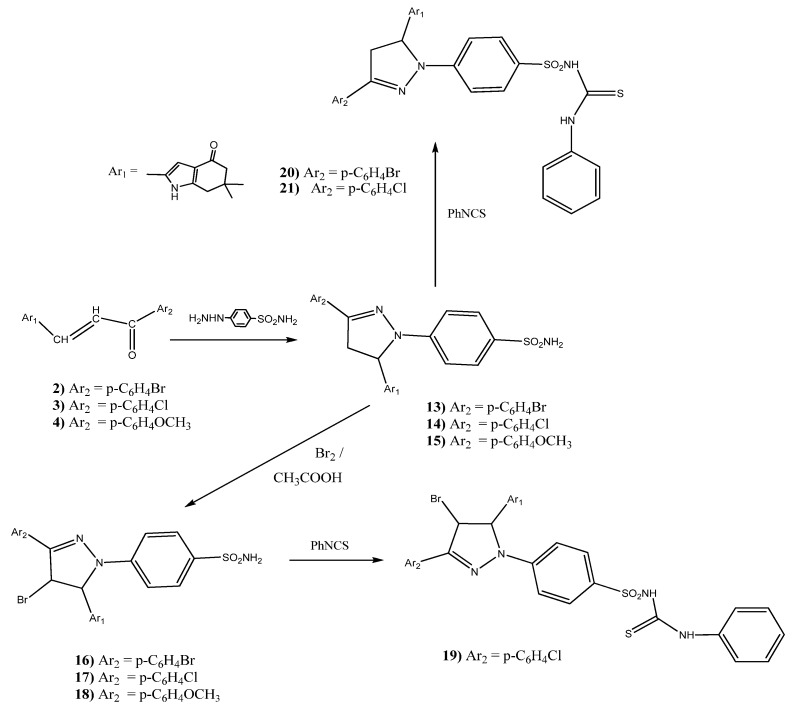
Synthesis of benzenesulfonamide derivatives **13**–**21**.

Reaction of the prepared chalcones **2**–**4** with 4-hydrazinyl benzenesulfonamide hydrochloride afforded 4-(3-(aryl)-5-(6,6-dimethyl-4-oxo-4,5,6,7-tetrahydro-1*H*-indol-2-yl)-4,5-dihydro-1*H*-pyrazol-1-yl)benzenesulfonamides **13**–**15** (Scheme 2).

Their ^1^H-NMR spectra showed three signals at 3.15–3.40, 3.36–4.00, and 4.80–5.56 ppm corresponding to the H_4_, H_4'_ , and H_5_ of the pyrazoline ring. In addition a broad singlet was observed in the 6.63–7.35 ppm range corresponding to the NH_2_ protons. The ^13^C-NMR spectrum of compound **15** as a prototype showed C=O at 193.8 ppm. On the other hand, the reaction of pyrazolines **13**–**15** with bromine in acetic acid [68] at room temperature in order to obtain the pyrazoles, afforded the corresponding substituted 4-(4-bromo-3-(4-bromophenyl)-5-(6,6-dimethyl-4-oxo-4,5,6,7-tetrahydro-1*H*-indol-2-yl)-1*H*-pyrazol-1-yl)benzenesulfonamide derivatives **16**–**18**, respectively in 79–84% yield.

Furthermore, the prepared compound **17** was treated with phenyl isothiocyanate to furnish 4-(4-bromo-3-aryl-5-(6,6-dimethyl-4-oxo-4,5,6,7-tetrahydro-1*H*-indol-2-yl)-1*H*-pyrazol-1-yl)-*N*-(phenyl-carbamothioyl)benzene sulfonamide **19** in 76.8% yield (Scheme 2). The proton NMR spectrum showed three broad singlets at 8.62, 9.76, and 11.11 ppm corresponding to three NH protons.

The prepared substituted benzenesulfonamides **13** and **14** were allowed to react with phenyl isothiocyanate to correspondingly furnish 4-(3-(aryl)-5-(6,6-dimethyl-4-oxo-4,5,6,7-tetrahydro-1*H*-indol-2-yl)-4,5-dihydro-1*H*-pyrazol-1-yl)-*N*-(phenylcarbamothioyl)benzenesulfonamides **20** and **21** (Scheme 2). Their ^1^H-NMR spectra showed D_2_O exchangeable signals at the ranges 9.00–9.15, 10.54–10.60, and 11.03–11.08 ppm, corresponding to three NH protons.

#### 2.1.4. Synthesis of [1,2,4]Triazolo[3,4-c][1,2,4]triazino[5,6-b]-5-*N*-(phenylcarbamothioyl) Ethanoic Acid Hydrazide Derivatives **30**, **31** and 3-Methyl-4-(propan-2-ylidene)-1*H*-pyrazol-5(4*H*)-one Derivatives **34**, **35**

**Scheme 3:**
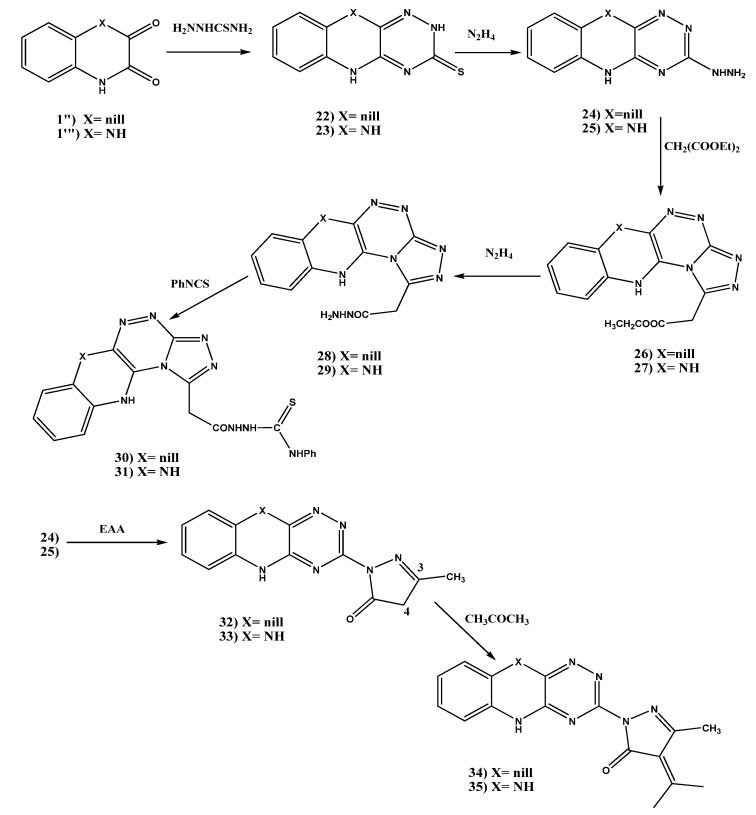
Synthesis of [1,2,4]triazolo[3,4-*c*][1,2,4]triazino[5,6-*b*]-5-*N*-(phenylcarbamothioyl) ethanoic acid hydrazide derivatives **30**, **31** and 3-methyl-4-(propan-2-ylidene)-1*H*-pyrazol-5(4*H*)-one derivatives **34**, **35**.

Reaction of indoline-2,3-dione (**1**'') [69,70] with thiosemicarbazide gave rise to 5*H*-[1,2,4] triazino[5,6-*b*]indole-3-thiol (**22**) [70] (Scheme 3). The ^1^H-NMR spectrum showed two broad singlets exchangeable with D_2_O at 12.43 and 14.54 ppm, corresponding to the two NH protons, which confirm the structure of **22**. The ^13^C-NMR also confirmed the structure of **22** with a peak at 179.5 corresponding to the C=S group. Treatment of the thiol derivative **22** with hydrazine hydrate afforded 3-hydrazinyl-*5H*-[1,2,4]triazino[5,6-*b*]indole (**24**) [70] in 92.6% yield (Scheme 3). The proton NMR spectrum showed two broad singlets at 4.31 and 8.54 ppm corresponding to NH_2_ and NH protons of hydrazine chain, in addition to a broad singlet at 11.82 ppm corresponding to the indole ring NH.

The reaction of **24** with diethyl malonate gave rise to the corresponding ester **26**. The proton NMR spectrum showed a triplet signal at 1.16 ppm corresponding to the CH_3_ protons, and a quartet signal at 4.31 ppm corresponding to CH_2_ of the ester moiety, and a singlet signal at 4.12 corresponding to CH_2_ protons. Reaction of the ester **26** with hydrazine hydrate afforded the corresponding 5-ethanoic hydrazide **28** (Scheme 3). From the proton NMR spectrum the disappearance of CH_3_ and CH_2_ protons of the ester chain can be observed. Treatment of the prepared hydrazide **28** with phenyl isothiocyanate afforded the corresponding 5-*N*-(phenylcarbamothioyl)ethanoic acid hydrazide **30** in 80.1% yield (Scheme 3). Its structure was confirmed by ^1^H-NMR, ^13^C-NMR spectra, and elemental analysis. The ^13^C-NMR spectrum showed C=S and C=O carbons at 168.7 and 189.4 ppm respectively (see Experimental part).

On the other hand, treatment of the prepared hydrazine derivative **24** with ethyl acetoacetate in acetic acid afforded 1-(*5H*-[1,2,4]triazino[5,6-*b*]indol-3-yl)-3-methyl-1*H*-pyrazol-5(4*H*)-one (**32**) (Scheme 3). The proton NMR spectrum of this compound showed the CH_3_ protons as a singlet at 1.80 ppm, and the CH_2_ protons as a singlet at 2.43 ppm. 1-(*5H*-[1,2,4]Triazino[5,6-*b*]indol-3-yl)-3-methyl-4-(propan-2-ylidene)-1*H*-pyrazol-5(4*H*)-one (**34**) was prepared from the previous pyrazoline-5-one derivative **32** by its reaction with acetone (Scheme 3). The proton NMR spectrum showed two methyl protons as a singlet at 1.87 ppm.

Cyclization of quinoxaline-2,3(1*H*,4*H*)-dione (**1**''') with thiosemicarbazide afforded 5,10-dihydro-[1,2,4]triazino[5,6-*b*]quinoxaline-3-thiol (**23**) in good yield (Scheme 3). Its proton NMR spectrum showed two broad singlets, exchangeable with D_2_O, at 11.88 and 14.50 ppm, corresponding to the three NH protons, which confirm the structure of **23**. Treatment of this thiol derivative **23** with hydrazine hydrate gave 3-hydrazinyl-5,10-dihydro-[1,2,4]triazino[5,6-*b*]quinoxaline (**25**, Scheme 3). Its proton NMR spectrum showed NH_2_ protons as a broad singlet signal at 4.55 ppm, in addition to three NH protons, see Experimental part. Treatment of the prepared hydrazine derivative **25** with diethyl malonate gave rise to the corresponding ester **27** (Scheme 3). The proton NMR spectrum showed the ester protons (CH_3_, CH_2_) as a triplet and a quartet signals at 1.09 and 4.09 ppm, respectively, in addition to CH_2_ protons at 4.66 ppm as a singlet signal.

Reaction of the ester **27** with hydrazine hydrate leads to the corresponding 5-ethanoic hydrazide **29**. The proton NMR spectrum showed the disappearance of CH_3_ and CH_2_ protons of the ester chain, and NH and NH_2_ protons at 8.90 and 9.61 ppm were observed as a two broad singlets. Its ^13^C-NMR spectrum showed the carbonyl carbon at 168.4 ppm. Treatment of the prepared hydrazide **29** with phenyl isothiocyanate leads to corresponding 5-*N*-(phenylcarbamothioyl) ethanoic acid hydrazide **31** (Scheme 3). Its structure was also confirmed by ^1^H-NMR, and elemental analysis.

Reaction of the hydrazine derivative **25** with ethyl acetoacetate in acetic acid afforded 1-(5,10-dihyro-[1,2,4]triazino[5,6-*b*]quinoxalin-3-yl)-3-methyl-1*H*-pyrazol-5(4*H*)-one (**33**, Scheme 3). The proton NMR spectrum showed the CH_3_ protons at position 3 of the pyrazoline ring as a singlet signal at 2.29 ppm, and the CH_2_ protons (H_4_) of the pyrazoline ring as a singlet signal at 2.92 ppm, with the disappearance of the peak corresponding to NH_2_ protons. ^13^C-NMR spectrum showed the carbonyl carbon at 170.0 ppm. Reaction of the prepared pyrazoline-5-one **33** with acetone gave rise to 1-(5,10-dihydro-[1,2,4]triazino[5,6-*b*]quinoxalin-3-yl)-3-methyl-4-(propan-2-ylidene)-1*H*-pyrazol-5(4*H*)-one (**35**, Scheme 3). Its proton NMR spectrum showed three methyl groups at 2.30, 2.42, and 2.93 ppm.

#### 2.1.5. Synthesis of Schiff’s Bases **36**, **37**, 4-Oxo-4,5,6,7-tetrahydro-1*H*-indol-2-yl)thiazolidin-4-one (**38**), Benzohydrazide Derivative **39**, 1,2,4]Triazolo[3,4-c]-5,10-dihydro [1,2,4]triazino[5,6-b] quinoxaline (**40**), and Pyrazole Derivatives **41–44**

**Scheme 4:**
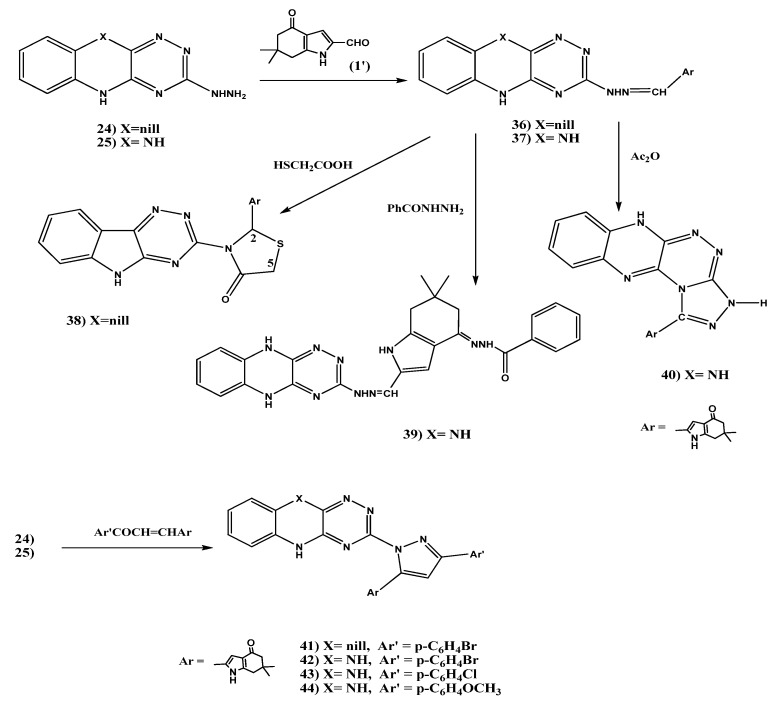
Synthesis of Schiff’s bases **36**, **37**, 4-oxo-4,5,6,7-tetrahydro-1*H*-indol-2-yl)thiazolidin-4-one (**38**), benzohydrazide derivative **39**, 1,2,4]triazolo[3,4-*c*]-5,10-dihydro [1,2,4]triazino[5,6-*b*] quinoxaline (**40**), and pyrazole derivatives **41**–**44**.

Condensation of hydrazine derivative **24** with 6,6-dimethyl-4-oxo-4,5,6,7-tetrahydro-1*H*-indole-2-carbaldehyde (**1**') afforded the corresponding Schiff's base **36** (Scheme 4). Its proton NMR spectrum showed the disappearance of the NH_2_ signal, and a singlet signal corresponding to a CH=N proton at 8.03 ppm was observed. The ^13^C-NMR spectrum showed the carbonyl carbon at 192.6 ppm. Treatment of the prepared compound **36** with thioglycolic acid in dry benzene gave rise to corresponding 3-(5*H*-[1,2,4]triazino[5,6-*b*]indol-3-yl)-2-(6,6-dimethyl-4-oxo-4,5,6,7-tetrahydro-1*H*-indol-2-yl) thiazolidin-4-one (**38**, Scheme 4). The proton NMR spectrum showed the CH_2_ (H_5_, H_5'_) protons of the thiazolidine ring at 3.35–3.49 ppm as a multiplet and the CH proton of thiazolidine ring (H_2_) as a singlet signal at 7.93 ppm.

Condensation of the hydrazine derivative **25** with 6,6-dimethyl-4-oxo-4,5,6,7-tetrahydro-1*H*-indole-2-carbaldehyde (**1**') furnished to the corresponding Schiff's base **37** in 86.6% yield (Scheme 4). Its proton NMR spectrum showed the CH=N proton as a singlet at 9.45 ppm. Condensation of **37** with benzoyl hydrazine afforded the corresponding *N'*-2-((2-(5,10-dihydro-[1,2,4]triazino[5,6-*b*]quinoxalin-3-yl)hydrazono)methyl)-6,6-dimethyl-6,7-dihydro-1*H*-indol-4(5*H*)-ylidene)benzohydrazide (**39**). Its ^13^C-NMR spectrum showed the C=O group at 162.7 ppm. On the other hand, oxidative cyclization of Schiff's base **37** with acetic anhydride afforded the cyclized compound **40** (Scheme 4). The proton NMR spectrum showed the disappearance of CH=N proton. We expected to obtain the acetylated product, but the ^1^H-NMR spectrum confirmed the structure of the cyclized compound **40 **as shown, with an exchangeable peak corresponding to three NH protons being observed at 12.23 ppm, and no peak observed corresponding to the acetyl methyl group. Oxidative cyclization of 3-hydrazinyl-5*H*-[1,2,4]triazino[5,6-*b*]indole (**24**) with 2-(3-(4-bromophenyl)-3-oxoprop-1-enyl)-6,6-dimethyl-6,7-dihydro-1*H*-indol-4(5*H*)-one (**2**) afforded 2-(1-(5*H*-[1,2,4]triazino[5,6-*b*]indol-3-yl)-3-(4-bromo-phenyl)-1*H*-pyrazol-5-yl)-(6,6-dimethyl-6,7-dihydro-1*H*-indol-4 (5*H*)-one (**41**) in 80.8% yield. The proton NMR spectrum showed the CH proton of pyrazole ring as a singlet signal at 6.12 ppm. The ^13^C-NMR spectrum showed C=O carbon 190.8 at ppm.

**Scheme 5:**
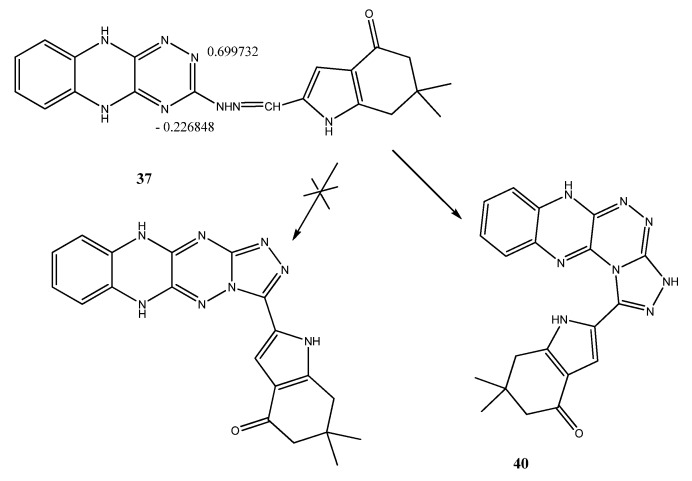
Charge distribution on nitrogen atoms N_1_, N_3_ of compound **37**.

On the other hand, the hydrazine derivative **25** was allowed to react with the prepared chalcones **2**–**4** to correspondingly furnish 2-(3-(aryl)-1-(5,10-dihydro-[1,2,4]triazino[5,6-*b*]quinoxalin-3-yl)-1*H*-pyrazol-5-yl)-6,6-dimethyl-6,7-dihydro-1*H*-indol-4(5*H*)-ones **42**–**44**, respectively (Scheme 4). Their proton NMR spectra showed the CH protons of the pyrazole ring (H_4_) and indole ring (H_3_) at 6.80 and 6.91, 6.90 and 7.27 and 6.78 and 6.85, ppm respectively. The ^13^C-NMR spectra of compounds **42**–**44** showed the C=O of the indole ring at 192.8–193.3 ppm. According to the charge distribution determined using ChemDraw Ultra, the N_1_ nitrogen atom has a better nucleophile character compared to the N_3_ nitrogen atom, which is in accordance with the proposed structure of compound **40** (Scheme 5).

### 2.2. Pharmacological Screening

Four test organisms representing different groups of microorganisms were used to evaluate the bioactivity of the designed products. The utilized test organisms were: *Escherichia coli* ATCC8739, *Pseudomonas aeruginosa* ATCC 9027 as Gram-negative bacteria, *Staphylococcus aureus* ATCC 6538P as an example of Gram-positive bacteria, and *Candida albicans* ATCC 2091 as yeast-like fungi. The inhibition zone (IZ) and minimal inhibitory zone (MIC) results are given in Table 1.

**Table 1:** *In vitro* antimicrobial activity of the test compounds and evaluation of the inhibition zone (IZ) and the minim. inhibitory concentration (MIC).

Microorganism	*Escherichia coli*		*Staphylococcus * *aureus*		*Candida albicans*		*Pseudomonas aeruginosa*	
	IZ	MIC	IZ	MIC	IZ	MIC	IZ	MIC
ampicillin 10.0 µg/disc	18	25	22	12.5	-----	------	-----	-----
ciprofloxacin 5.0 µg/disc	28	12.5	30	25	-----	------	38	25
clotrimazole 100.0 µg/disc	----	-----	-----	------	40	12.5	-----	------
imipenam 10.0 µg/disc	26	-----	30	------	-----	----	30	------
2	18	200	17	200	21	200	16	200
3	19	200	17	200	24	200	18	200
4	19	200	15	200	23	200	18	200
5	19	200	15	200	23	200	19	200
6	19	200	16	200	25	200	20	200
7	18	200	13	200	21	200	16	200
8	19	200	17	200	22	200	18	200
9	18	200	16	200	24	200	18	200
10	19	200	16	200	24	200	20	200
11	19	200	18	100	23	200	19	200
12	19	200	15	200	23	200	18	200
13	18	200	19	200	23	200	18	200
14	18	200	15	200	23	200	18	200
15	18	200	16	100	22	200	19	200
16	19	200	26	**25**	27	50	20	200
17	19	200	>**50**	100	>**40**	50	21	100
18	19	200	20	100	23	200	19	200
19	19	200	16	100	25	**12.5**	19	200
20	18	200	16	200	26	**12.5**	18	200
21	18	200	16	200	28	50	20	200
22	19	200	16	200	25	50	18	200
23	18	200	8	200	24	200	20	200
24	18	200	16	200	23	200	20	200
25	19	200	15	200	22	200	18	200
26	19	200	13	200	23	200	19	200
27	19	200	17	200	25	200	17	200
28	19	200	17	200	23	200	18	200
29	18	200	15	200	21	200	18	200
30	19	200	17	200	26	200	16	200
31	19	200	16	200	34	**25**	20	200
32	19	200	16	200	23	200	20	200
33	19	200	17	200	24	200	18	200
34	19	200	17	200	24	200	17	200
35	19	200	16	100	23	200	19	200
36	19	200	17	200	24	200	18	200
37	17	200	15	200	22	200	18	200
38	19	200	17	200	21	200	18	200
39	19	200	17	200	22	200	16	200
40	19	200	17	200	21	200	18	200
41	18	200	13	200	21	200	16	200
42	18	200	16	200	23	200	18	200
43	18	200	17	100	24	200	19	200
44	20	200	17	100	23	200	19	200
DMF	18		13		21		16	

The compounds under investigation **2**–**44** did not show any activity against the test organisms *Escherichia coli* and *Pseudomonas aeruginosa*. The inhibition Zone (IZ) listed in Table 1 showed that compound **16** has good antimicrobial activity against *Staphylococcus aureus*, comparable to that of ampicillin, while compound **17** has remarkable antimicrobial activity against *Staphylococcus aureus* exceeding that of ampicillin, ciprofloxacin and imipenam.. The minimal inhibitory concentration (MIC) value showed that compound **16** has good antimicrobial activity against *Staphylococcus aureus*, comparable to that of ciprofloxacin, while its activity is about 50% of that of ampicillin. In addition, compound **17** has an IZ against *Candida albicans *comparable to that of clotrimazole. The minimal inhibitory concentration (MIC) of compound **17** against *Candida albicans *is about 25% of that clotrimazole. On the other hand, the minimal inhibitory concentration (MIC) of compounds **19** and **20** against *Candida albicans *was good, and comparable to that of clotrimazole, while compound **31** has 50 % activity compared to that of clotrimazole.

## 3. Experimental

### 3.1. General Methods

Fresh solvents were used without purification. Melting points were obtained in open capillary tubes by using a MEL-Temp II melting point apparatus and are uncorrected. Infrared spectra (IR) were recorded on a Perkin-Elmer 1600 series Fourier Transform instrument with the samples as KBr pellets. ^1^H-NMR and ^13^C-NMR spectra were recorded on a JEOL 500 MHz spectrometer at ambient temperature using tetramethylsilane as an internal reference. Elemental analyses were carried out by the University of Cairo Microanalytical Laboratories. The antimicrobial tests were carried out at the Pharmaceutical Chemistry Department, Faculty of Pharmacy, Alexandria University. ChemDraw-Ultra-11.0 has been used for the nomenclature of the prepared compounds.

### 3.2. General Procedure for the Preparation of Compounds ***2–4***

An equimolar mixture of 6,6-dimethyl-4-oxo-4,5,6,7-tetrahydro-1*H*-indole-2-carbaldehyde (**1**', 1.91 g, 0.01 mol) [67] and the substituted acetophenone (0.01 mol) in 2% ethanolic KOH (20 mL) was stirred at room temperature (R.T.) for 5 h. The solid product was cooled, collected by filtration, washed with water, dried and recrystallized from chloroform/ethanol.

*2-(3-(4-Bromophenyl)-3-oxoprop-1-enyl)-6,6-dimethyl-6,7-dihydro-1H-indol-4(5H)-one* (**2**). Yellow crystals; yield 3.36 g, 90.5%; m.p. 269–270 °C; IR (KBr): 1651 (C=O), 3252 cm^−1^ (NH); ^1^H-NMR (DMSO-*d*_6_) δ 1.01 (s, 6H, 2 CH_3_), 2.23 (s, 2H, CH_2_), 2.72 (s, 2H, CH_2_), 6.91 (s, 1H, CH-pyrrole), 7.54 (d, 1H, COCH=; *J* = 15.3 Hz), 7.60 (d, 1H, CH=; *J* = 15.3 Hz), 7.76 (d, 2H, ArH; *J =* 8.4 Hz), 7.93 (d, 2H, ArH; *J* = 8.4 Hz), 12.14 (bs, 1H, NH; exchangeable with D_2_O); ^13^C-NMR (DMSO*-d*_6_) δ:28.6, 35.7, 36.5, 52.2, 113.5, 116.7, 121.0, 127.5, 130.6, 132.4, 134.6, 137.5, 147.8, 187.8, 192.7. Anal. Calcd for C_19_H_18_BrNO_2_ (372.26): C, 61.30; H, 4.87; N, 3.76 Found: C, 61.42; H, 4.97; N, 3.53.

*2-(3-(4-Chlorophenyl)-3-oxoprop-1-enyl)-6,6-dimethyl-6,7-dihydro-1H-indol-4(5H)-one* (**3**). Yellow crystals; yield 2.99 g, 91.5%; m.p. 261–262 °C; IR (KBr): 1651 (C=O), 3250 cm^−1^ (NH); ^1^H-NMR (DMSO-*d*_6_) δ 1.01 (s, 6H, 2 CH_3_), 2.23 (s, 2H, CH_2_), 2.72 (s, 2H, CH_2_), 6.91 (s, 1H, CH-pyrrole), 7.52–7.63 (m, 4H, CH=CH, 2 ArH), 8.01 (d, 2H, ArH; *J* = 8.4 Hz), 12.12 (bs, 1H, NH; exchangeable with D_2_O). Anal. Calcd for C_19_H_18_ClNO_2_ (327.80): C, 69.62; H, 5.53; N, 4.27 Found: C, 69.82; H, 5.76; N, 3.99.

*2-(3-(4-Methoxyphenyl)-3-oxoprop-1-enyl)-6,6-dimethyl-6,7-dihydro-1H-indol-4(5H)-one* (**4**). Yellow crystals; yield 3.00 g, 93.0%; m.p. 240–241 °C; IR (KBr): 1651 (C=O), 3232 cm^−1^ (NH); ^1^H-NMR (DMSO-*d*_6_) δ 1.01 (s, 6H, 2 CH_3_), 2.23 (s, 2H, CH_2_), 2.71 (s, 2H, CH_2_), 3.82 (s, 3H, OCH_3_), 6.85 (s, 1H, CH-pyrrole), 7.07 (d, 2H, ArH; *J* = 8.4 Hz), 7.48 (d, 1H, -COCH=; *J* = 15.3 Hz), 7.60 (d, 1H, CH=; *J* = 15.3 Hz), 8.01 (d, 2H, ArH; *J* = 8.4 Hz), 12.03 (bs, 1H, NH; exchangeable with D_2_O). Anal. Calcd for C_20_H_21_NO_3_ (323.39): C, 74.28; H, 6.55; N, 4.33 Found: C, 74.40; H, 6.71; N, 4.11.

### 3.3. General Procedure for the Preparation of Compounds ***5–7***

A mixture of the appropriate 2-(3-aryl-3-oxoprop-1-enyl)-6,6-dimethyl-6,7-dihydro-1*H*-indol-4(5*H*)-one **2**–**4** (0.002 mol) and thiosemicarbazide (0.003 mol) was dissolved in a mixture of acetone and ethanol (30 mL), then potassium carbonate (0.004 mol) was added with vigorous stirring. Heating under reflux was performed for 14 h. The solvent was removed under vacuum, and ice-water was added to the reaction mixture. The solid product obtained was filtered, washed with ethanol, dried and recrystallized from chloroform/ethanol.

*3-(4-Bromophenyl)-5-(6,6-dimethyl-4-oxo-4,5,6,7-tetrahydro-1H-indol-2-yl)-4,5-dihydro-1H-pyrazole-1-carbothioamide* **5**. Buff crystals; yield 0.71 g, 80.0%; m.p. 192–193 °C; IR (KBr): 1167 (C=S), 1651 (C=O), 3134, 3232, 3436 cm^−1^ (NH, NH_2_); ^1^H-NMR (DMSO-*d*_6_) δ 1.13 (s, 6H, 2 CH_3_), 2.39 (s, 2H, CH_2_), 2.80 (s, 2H, CH_2_), 3.86–3.89 (m, 2H, H_4,_ H_4'_-pyrazoline), 6.73–6.88 (dd, 1H, H_5_-pyrazoline; *J* = 17.4, 12.0 Hz), 6.92 (s, 1H, CH-pyrrole), 6.97 (d, 2H, ArH; *J* = 8.6 Hz), 7.25 (bs, 2H, NH_2_; exchangeable with D_2_O), 8.01 (d, 2H, ArH; *J* = 8.6 Hz), 12.05 (bs, 1H, NH; exchangeable with D_2_O). Anal. Calcd for C_20_H_21_BrN_4_OS (445.38): C, 53.94; H, 4.75; N, 12.58 Found: C, 53.70; H, 4.60; N, 12.76.

*3-(4-Chlorophenyl)-5-(6,6-dimethyl-4-oxo-4,5,6,7-tetrahydro-1H-indol-2-yl)-4,5-dihydro-1H-pyrazole-1-carbothioamide* **6**. Buff crystals; yield 0.63 g, 79.8%; m.p. 210–211 °C; IR (KBr): 1092 (C=S), 1646 (C=O), 3237, 3428 cm^−1^ (NH, NH_2_); ^1^H-NMR (DMSO-*d*_6_) δ 0.95 (s, 3H, CH_3_), 1.02 (s, 3H, CH_3_), 2.24 (s, 2H, CH_2_), 2.72 (s, 2H, CH_2_), 3.33–3.41 (m, 2H, H_4,_ H_4’_-pyrazoline), 6.80–6.88 (dd, 1H, H_5_-pyrazoline; *J* = 17.4, 12.0 Hz), 7.58 (s, 1H, CH-pyrrole), 7.47 (bs, 2H, NH_2_; exchangeable with D_2_O), 7.77 (d, 2H, ArH; *J* = 7.5 Hz), 7.93 (d, 2H, ArH; *J* = 7.5 Hz), 12.00 (bs, 1H, NH; exchangeable with D_2_O); ^13^C-NMR (DMSO*-d*_6_) δ: 29.0, 31.4, 40.4, 41.0, 52.5, 63.5, 114.4, 120.2, 125.3, 129.3, 129.5, 130.6, 136.0, 140.0, 150.49, 180.0, 204.6. Anal. Calcd for C_20_H_21_ClN_4_OS (400.92): C, 59.91; H, 5.28; N, 13.97 Found: C, 59.70; H, 5.11; N, 14.12.

*5-(6,6-Dimethyl-4-oxo-4,5,6,7-tetrahydro-1H-indol-2-yl)-3-(4-methoxyphenyl)-4,5-dihydro-1H-pyrazole-1-carbothioamide* **7**. Yellow crystals; yield 0.60 g, 76.7%; m.p. 259–260 °C; IR (KBr): 1167 (C=S), 1651 (C=O), 3134, 3232, 3436 cm^−1^ (NH, NH_2_); ^1^H-NMR (DMSO-*d*_6_) δ 1.13 (s, 6H, 2 CH_3_), 2.39 (s, 2H, CH_2_), 2.80 (s, 2H, CH_2_), 3.86–3.89 (m, 4H, H_4_-pyrazoline, OCH_3_), 4.73–4.82 (m, 1H, H_4'_-pyrazoline), 6.92–6.98 (m, 1H, H_5_-pyrazoline), 7.15–7.25 (m, 5H, 4 ArH, CH-pyrrole), 8.01 (bs, 2H, NH_2_; exchangeable with D_2_O), 11.59 (bs, 1H, NH; exchangeable with D_2_O). Anal. Calcd for C_21_H_24_N_4_O_2_S (396.51): C, 63.61; H, 6.10; N, 14.13 Found: C, 63.80; H, 6.24; N, 13.90.

### 3.4. General Procedure for the Preparation of Compounds ***8*** and ***9***

A mixture of 2-(3-aryl-3-oxoprop-1-enyl)-6,6-dimethyl-6,7-dihydro-1*H*-indol-4(5*H*)-one **2** or **3** (0.001 mol), hydrazinum chloride (0.003 mol) and anhydrous sodium acetate (0.003 mol), in ethanol (15 mL) and glacial acetic acid (5 mL) was refluxed for 8 h. The reaction mixture was poured over crushed ice. The solid obtained was filtered, washed with water, dried and recrystallized from ethanol.

*2-(3-(4-Bromophenyl)-4,5-dihydro-1H-pyrazol-5-yl)-6,6-dimethyl-6,7-dihydro-1H-indol-4(5H)-one* (**8**). Brown crystals; yield 0.31 g, 81.3%; m.p. 210–211 °C; IR (KBr): 1641 (C=O), 3260, 3436 cm^−1^ (NH); ^1^H-NMR (DMSO-*d*_6_) δ 0.98 (s, 6H, 2 CH_3_), 2.24 (s, 2H, CH_2_), 2.72 (s, 2H, CH_2_), 3.33–3.75 (m, 2H, H_4_, H_4'_-pyrazoline), 6.50–6.70 (m, 1H, H_5_-pyrazoline), 6.91 (s, 1H, CH-pyrrole), 7.77 (d, 2H, ArH; *J* = 7.7 Hz), 7.93 (d, 2H, ArH; *J* = 7.7 Hz), 11.25 (bs, 1H, NH; exchangeable with D_2_O), 12.08 (bs, 1H, NH; exchangeable with D_2_O). Anal. Calcd for C_19_H_20_BrN_3_O (386.29): C, 59.08; H, 5.22; N, 10.88 Found: C, 59.26; H, 5.45; N, 10.65.

*2-(3-(4-Chlorophenyl)-4,5-dihydro-1H-pyrazol-5-yl)-6,6-dimethyl-6,7-dihydro-1H-indol-4(5H)-one* (**9**). Buff crystals; yield 0.26 g, 78.8%; m.p. 195–196 °C; IR (KBr): 1642 (C=O), 3209, 3423 cm^−1^ (NH); ^1^H-NMR (DMSO-*d*_6_) δ 0.98 (s, 3H, CH_3_), 1.01 (s, 3H, CH_3_), 2.20 (s, 2H, CH_2_), 2.42 (s, 2H, CH_2_), 3.53–3.75 (m, 1H, H_4_-pyrazoline), 3.69–3.75 (m, 1H, H_4'_-pyrazoline), 5.50 (m, 1H, H_5_-pyrazoline), 6.69 (s, 1H, CH-pyrrole), 7.41 (d, 2H, ArH; *J* = 7.7 Hz), 7.78 (d, 2H, ArH; *J* = 7.7 Hz), 11.19 (bs, 1H, NH; exchangeable with D_2_O), 12.07 (bs, 1H, NH; exchangeable with D_2_O). Anal. Calcd for C_19_H_20_ClN_3_O (341.83): C, 66.76; H, 5.90; N, 12.29 Found: C, 66.53; H, 5.69; N, 12.50.

### 3.5. General Procedure for the Preparation of Compounds ***10–12***

A mixture of 2-(3-aryl-3-oxoprop-1-enyl)-6,6-dimethyl-6,7-dihydro-1*H*-indol-4(5*H*)-ones **2**–**4** (0.001 mol), hydroxylamine hydrochloride (0.003 mol) and anhydrous sodium acetate (0.003 mol), in ethanol (15 mL) and glacial acetic acid (5 mL) was refluxed for 8 h. The reaction mixture was poured over crushed ice. The solid obtained was filtered, washed with water, dried and recrystallized from ethanol.

*2-(3-(4-Bromophenyl)-4,5-dihydroisoxazol-5-yl)-6,6-dimethyl-6,7-dihydro-1H-indol-4(5H)-one* (**10**). Yellow crystals; yield 0.34 g, 90.1%; m.p. 115–116 °C; IR (KBr): 1646 (C=O), 3423 cm^−1^ (NH); ^1^H-NMR (DMSO-*d*_6_) δ 0.95 (s, 6H, 2 CH_3_), 2.42 (s, 2H, CH_2_), 2.51 (s, 2H, CH_2_), 3.34–3.46 (m, 2H, H_4_, H_4'_-isoxazoline), 6.50–6.52 (m, 1H, H_5_ isoxazoline), 6.84 (s, 1H, CH-pyrrole), 7.49–7.56 (m, 4H, ArH), 11.32 (bs, 1H, NH; exchangeable with D_2_O). Anal. Calcd for C_19_H_19_BrN_2_O_2_ (387.27): C, 58.93; H, 4.95; N, 7.23 Found: C, 58.75; H, 4.86; N, 7.39.

*2-(3-(4-Chlorophenyl)-4,5-dihydroisoxazol-5-yl)-6,6-dimethyl-6,7-dihydro-1H-indol-4(5H)-one* (**11**). Buff crystals; yield 0.26 g, 78.3%; m.p. 160–161 °C; IR (KBr): 1643 (C=O), 3433 cm^−1^ (NH); ^1^H-NMR (DMSO-*d*_6_) δ 1.01 (s, 6H, 2 CH_3_), 2.35 (s, 2H, CH_2_), 2.59 (s, 2H, CH_2_), 3.50–3.55 (m, 1H, H_4_-isoxazoline), 3.66–3.72 (m, 1H, H_4'_-isoxazoline), 5.66–5.70 (m, 1H, H_5_ isoxazoline), 6.81 (s, 1H, CH-pyrrole), 7.51 (d, 2H, ArH; *J* = 6.7 Hz), 7.70 (d, 2H, ArH; *J* = 6.7 Hz), 11.97 (bs, 1H, NH; exchangeable with D_2_O). ^13^C-NMR (DMSO*-d*_6_) δ: 27.9, 34.4, 36.6, 43.3, 55.0, 76.8, 108.1, 109.8, 128.1, 128.9, 129.4, 131.9, 135.2, 143.6, 156.5, 193.7. Anal. Calcd for C_19_H_19_ClN_2_O_2_ (342.82): C, 66.57; H, 5.59; N, 8.17 Found: C, 66.40; H, 5.35; N, 8.34.

*2-(3-(4-Methoxyphenyl)-4,5-dihydroisoxazol-5-yl)-6,6-dimethyl-6,7-dihydro-1H-indol-4(5H)-one* (**12**). Buff crystals; yield 0.27 g, 81.0%; m.p. 170–171 °C; IR (KBr): 1645 (C=O), 3427 cm^−1^ (NH); ^1^H-NMR (DMSO-*d*_6_) δ 0.98 (s, 6H, 2 CH_3_), 2.46 (s, 4H, 2 CH_2_), 3.34 (s, 3H, OCH_3_), 3.46–3.63 (m, 1H, H_4_-isoxazoline), 4.08–4.22 (m, 1H, H_4'_-isoxazoline), 5.55–5.66 (m, 1H, H_5_ isoxazoline), 6.72 (s, 1H, CH-pyrrole), 6.98 (d, 2H, ArH; *J* = 6.9 Hz), 7.62 (d, 2H, ArH; *J* = 6.9 Hz), 11.16 (bs, 1H, NH; exchangeable with D_2_O). Anal. Calcd for C_20_H_22_N_2_O_3_ (338.40): C, 70.99; H, 6.55; N, 8.28 Found: C, 70.79; H, 6.31; N, 8.47.

### 3.6. General Procedure for the Preparation of Compounds ***13–15***

A mixture of 2-(3-aryl-3-oxoprop-1-enyl)-6,6-dimethyl-6,7-dihydro-1*H*-indol-4(5*H*)-one **2**–**4** (0.001 mol) and 4-hydrazinylbenzenesulfonamide hydrochloride (0.001 mol) in methanol (30 mL) was heated under reflux for 6 h, partially concentrated and cooled. The separated solid product was filtered, washed with ethanol, dried and recrystallized from ethanol.

*4-(3-(4-Bromophenyl)-5-(6,6-dimethyl-4-oxo-4,5,6,7-tetrahydro-1H-indol-2-yl)-4,5-dihydro-1H-pyrazol-1-yl)benzenesulfonamide* (**13**). Brown crystals; yield 0.48 g, 90.2%; mp 180–181 °C; IR (KBr): 1219, 1374 (SO_2_), 1651 (C=O), 3250, 3434 cm^−1^ (NH, NH_2_); ^1^H-NMR (DMSO-*d*_6_) δ 0.93 (s, 3H, CH_3_), 1.01 (s, 3H, CH_3_), 2.50 (s, 2H, CH_2_), 2.66 (s, 2H, CH_2_), 3.15–3.22 (m, 1H, H_4_-pyrazoline), 3.36–3.40 (m, 1H, H_4'_-pyrazoline), 5.53–5.56 (m, 1H, H_5_- pyrazoline), 6.43 (s, 1H, CH-pyrrole), 6.63 (bs, 2H, NH_2_; exchangeable with D_2_O), 7.00–7.26 (m, 2H, ArH), 7.40–7.70 (m, 2H, ArH), 7.73–7.93 (m, 2H, ArH), 8.05–8.18 (m, 2H, ArH), 11.61 (bs, 1H, NH; exchangeable with D_2_O). Anal. Calcd for C_25_H_25_BrN_4_O_3_S (541.46): C, 55.46; H, 4.65; N, 10.35 Found: C, 55.60; H, 4.87; N, 10.15.

*4-(3-(4-Chlorophenyl)-5-(6,6-dimethyl-4-oxo-4,5,6,7-tetrahydro-1H-indol-2-yl)-4,5-dihydro-1H-pyrazol-1-yl)benzenesulfonamide* (**14**). Brown crystals; yield 0.43 g, 87.5%; m.p. 150–151 °C; IR (KBr): 1219, 1374 (SO_2_), 1651 (C=O), 3256, 3438 cm^−1^ (NH, NH_2_); ^1^H-NMR (DMSO-*d*_6_) δ 1.01 (s, 6H, 2 CH_3_), 2.24 (s, 2H, CH_2_), 2.72 (s, 2H, CH_2_), 3.20–3.40 (m, 1H, H_4_-pyrazoline), 3.78–3.84 (dd, 1H, H_4'_-pyrazoline; *J* = 17.4, 12.0 Hz), 5.53–5.55 (m, 1H, H_5_-pyrazoline), 6.85 (s, 1H, CH-pyrrole), 7.13 (d, 2H, ArH; *J* = 9.1 Hz), 7.34 (bs, 2H, NH_2_; exchangeable with D_2_O), 7.43 (d, 2H, ArH; *J* = 9.1 Hz), 7.53 (d, 2H, ArH; *J* = 8.4 Hz), 7.69 (d, 2H, ArH; *J* = 8.4 Hz), 12.18 (bs, 1H, NH; exchangeable with D_2_O). Anal. Calcd for C_25_H_25_ClN_4_O_3_S (497.01): C, 60.41; H, 5.07; N, 11.27 Found: C, 60.66; H, 5.30; N, 11.03.

*4-(5-(6,6-Dimethyl-4-oxo-4,5,6,7-tetrahydro-1H-indol-2-yl)-3-(4-methoxyphenyl)-4,5-dihydro-1H-pyrazol-1-yl)benzenesulfonamide* (**15**). Buff crystals; yield 0.44 g, 91.1%; m.p. 224–225 °C; IR (KBr): 1158, 1307 (SO_2_), 1650 (C=O), 3263, 3345 cm^−1^ (NH, NH_2_); ^1^H-NMR (DMSO-*d*_6_) δ 1.01 (s, 6H, 2 CH_3_), 2.20 (s, 2H, CH_2_), 2.68 (s, 2H, CH_2_), 3.24–3.28 (m, 1H, H_4_-pyrazoline), 3.34 (s, 3H, OCH_3_), 3.76–4.00 (m, 1H, H_4'_-pyrazoline), 4.80–5.53 (m, 1H, H_5_-pyrazoline), 6.46 (s, 1H, CH-pyrrole), 7.00–7.04 (m, 4H, 2 ArH, NH_2_), 7.13 (d, 2H, ArH; *J* = 8.4 Hz), 7.60 (d, 2H, ArH; *J* = 8.4 Hz), 7.70–7.73 (m, 2H, ArH), 11.65 (bs, 1H, NH; exchangeable with D_2_O). ^13^C-NMR (DMSO*-d*_6_) δ: 28.7, 35.1, 40.9, 43.7, 53.6, 55.7, 60.3, 106.4, 114.7, 118.4, 119.9, 125.4, 127.6, 127.9, 130.3, 141.6, 142.3, 153.2, 160.0, 193.8. Anal. Calcd for C_26_H_28_N_4_O_4_S (492.59): C, 63.40; H, 5.73; N, 11.37 Found: C, 63.69; H, 5.90; N, 11.22.

### 3.7. General Procedure for the Preparation of Compounds ***16–18***

Solid 4-(3-aryl-5-(6,6-dimethyl-4-oxo-4,5,6,7-tetrahydro-1*H*-indol-2-yl)-4,5-dihydro-1*H*-pyrazol-1-yl)benzenesulfonamide **13**–**15** (0.001 mol) was dissolved in hot acetic acid, and after cooling bromine (0.3 mL) in acetic acid (10 mL) was added dropwise with shaking, then the reaction mixture was allowed to stand at R.T. overnight. The solid mass obtained was separated by filtration, washed with water, dried and recrystallized from ethanol.

*4-(4-Bromo-3-(4-bromophenyl)-5-(6,6-dimethyl-4-oxo-4,5,6,7-tetrahydro-1H-indol-2-yl)-1H-pyrazol-1-yl)benzenesulfonamide* (**16**). Buff crystals; yield 0.49 g, 79.3%; m.p. 200–201 °C; IR (KBr): 1162, 1335 (SO_2_), 1653 (C=O), 3253, 3433 cm^−1^ (NH, NH_2_); ^1^H-NMR (DMSO-*d*_6_) δ 1.09 (s, 6H, 2 CH_3_), 2.26 (s, 2H, CH_2_), 2.86 (s, 2H, CH_2_), 3.58–3.88 (m, 1H, H_4_-pyrazoline), 4.80–5.00 (m, 1H, H_5_ -pyrazoline), 7.44–7.46 (m, 3H, CH-pyrrole, NH_2_), 7.71 (d, 2H, ArH; *J* = 7.7 Hz), 7.86 (d, 2H, ArH; *J* = 8.4 Hz), 7.92 (d, 2H, ArH; *J* = 8.4 Hz), 8.07 (d, 2H, ArH; *J* = 7.7 Hz), 9.02 (bs, 1H, NH; exchangeable with D_2_O). Anal. Calcd for C_25_H_24_Br_2_N_4_O_3_S (620.36): C, 48.40; H, 3.90; N, 9.03 Found: C, 48.80; H, 3.76; N, 8.87.

*4-(4-Bromo-3-(4-chlorophenyl)-5-(6,6-dimethyl-4-oxo-4,5,6,7-tetrahydro-1H-indol-2-yl)-1H-pyrazol-1-yl)benzenesulfonamide* (**17**). Brown crystals; yield 0.47 g, 82.4%; m.p. 129–130 °C; IR (KBr): 1163, 1315 (SO_2_), 1670 (C=O), 3068, 3385 cm^−1^ (NH, NH_2_); ^1^H-NMR (DMSO-*d*_6_) δ 1.16 (s, 6H, 2 CH_3_), 2.27 (s, 2H, CH_2_), 2.47 (s, 2H, CH_2_), 3.13–3.73 (m, 1H, H_4_-pyrazoline), 3.88–4.16 (m, 1H, H_5_-pyrazoline), 7.56 (s, 1H, H-pyrrole), 7.63 (bs, 2H, NH_2_; exchangeable with D_2_O), 7.80–7.85 (m, 4H, ArH), 8.00–8.10 (m, 4H, ArH), 11.06 (bs, 1H, NH; exchangeable with D_2_O). Anal. Calcd for C_25_H_24_BrClN_4_O_3_S (575.91): C, 52.14; H, 4.20; N, 9.73 Found: C, 52.25; H, 4.00; N, 9.58.

*4-(4-Bromo-5-(6,6-dimethyl-4-oxo-4,5,6,7-tetrahydro-1H-indol-2-yl)-3-(4-methoxy-phenyl)-1H-pyrazol-1-yl)benzenesulfonamide* (**18**). Brown crystals; yield 0.48 g, 84.3%; m.p. 160–161 °C; IR (KBr): 1164, 1340 (SO_2_), 1650 (C=O), 3220, 3435 cm^−1^ (NH, NH_2_); ^1^H-NMR (DMSO-*d*_6_) δ 1.01 (s, 3H, CH_3_), 1.02 (s, 3H, CH_3_), 2.26 (s, 2H, CH_2_), 2.70 (s, 2H, CH_2_), 3.34 (s, 3H, OCH3), 3.80–3.89 (m, 2H, H_4_, H_5_-pyrazoline), 6.93 (d, 1H, ArH; *J* = *8.4* Hz), 7.03–7.07 (m, 2H, ArH, CH-pyrrole), 7.42 (d, 2H, ArH; *J* = 9.1 Hz), 7.82 (d, 2H, ArH; *J* = 9.1 Hz), 7.87–7.93 (m, 4H, 2ArH, NH_2_), 12.25 (bs, 1H, NH; exchangeable with D_2_O). Anal. Calcd for C_26_H_27_BrN_4_O_4_S (571.49): C, 54.64; H, 4.76; N, 9.80 Found: C, 55.01; H, 4.65; N, 9.68.

### 3.8. 4-(4-Bromo-3-aryl-5-(6,6-dimethyl-4-oxo-4,5,6,7-tetrahydro-1H-indol-2-yl)-1H-pyrazol-1-yl)-N-(phenylcarbamothioyl)benzenesulfonamide ***(19)***

A mixture of 4-(4-bromo-3-(4-chlorophenyl)-5-(6,6-dimethyl-4-oxo-4,5,6,7-tetrahydro-1H-indol-2-yl)-1H-pyrazol-1-yl) benzenesulfonamide (**17**, 0.005 mol) and anhydrous potassium carbonate (0.01 mol) in dry acetone (100 mL) was stirred under reflux for 15 h. A solution of phenyl isothiocyanate (0.007 mol) in dry acetone was added drop by drop at this temperature, and refluxing was continued for 12 h more. The acetone was distilled under reduced pressure and the solid residue was dissolved in water, the product was isolated after acidification with 2 N HCl. The solid mass obtained was separated by filtration, washed with water, dried and recrystallized from ethanol. The product was obtained as buff crystals; yield 2.73 g, 76.8%; m.p. 92–93 °C; IR (KBr): 1092 (C=S), 1160, 1345 (SO_2_), 1648 (C=O), 3435 cm^−1^ (NH); ^1^H-NMR (DMSO-d_6_) δ 1.19 (s, 6H, 2 CH_3_), 1.99 (s, 2H, CH_2_), 2.61 (s, 2H, CH_2_), 3.87–4.03 (m, 1H, H_4_-pyrazoline), 6.90–6.92 (m, 1H, H_5_-pyrazoline), 7.09 (s, 1H, CH-pyrrole), 7.22–7.27 (m, 2H, ArH), 7.29–7.32 (m, 3H, ArH), 7.40–7.45 (m, 4H, ArH), 7.85 (d, 2H, ArH; J = 8.4 Hz), 8.06 (d, 2H, ArH; J = 8.4 Hz), 8.62 (bs, 1H, NH; exchangeable with D_2_O), 9.76 (bs, 1H, NH; exchangeable with D_2_O), 11.11 (bs, 1H, NH; exchangeable with D_2_O). Anal. Calcd for C_32_H_29_BrClN_5_O_3_S_2_(711.09): C, 54.05; H, 4.11; N, 9.85 Found: C, 54.00; H, 3.96; N, 10.04.

### 3.9. General Procedure for the Preparation of Compounds ***20*** and ***21***

A mixture of 4-(3-aryl-5-(6,6-dimethyl-4-oxo-4,5,6,7-tetrahydro-1*H*-indol-2-yl)-4,5-dihydro-1*H*-pyrazol-1-yl)benzenesulfonamide **13** and **14** (0.005 mol) and anhydrous potassium carbonate (0.01 mol) in dry acetone (100 mL) was stirred with refluxing for 15 h. A solution of phenyl isothiocyanate (0.007 mol) in dry acetone was added drop by drop at this temperature, and refluxing was continued for 12 h. The acetone was distilled under reduced pressure and the solid residue was dissolved in water, the product was isolated after acidification with 2 N HCl. The solid mass obtained was separated by filtration, washed with water, dried and crystallized from ethanol.

*4-(3-(4-Bromophenyl)-5-(6,6-dimethyl-4-oxo-4,5,6,7-tetrahydro-1H-indol-2-yl)-4,5-dihydro-1H-pyrazol-1-yl)-N-(phenylcarbamothioyl)benzenesulfonamide* (**20**). the product was obtained as brown crystals; Yield 2.86 g, 84.6%; m.p. 210–211 °C; IR (KBr): 1089 (C=S), 1160, 1355 (SO_2_), 1646 (C=O), 3436 cm^−1^ (NH); ^1^H-NMR (DMSO-*d*_6_) δ 1.19 (s, 6H, 2 CH_3_), 2.69 (s, 2H, CH_2_), 2.85 (s, 2H, CH_2_), 3.50–3.60 (m, 1H, H_4_-pyrazoline), 4.08–4.15 (m, 1H, H_4'_-pyrazoline), 4.40–4.55 (m, 1H, H_5_-pyrazoline), 6.57 (s, 1H, CH-pyrrole), 7.10–7.18 (m, 2H, ArH), 7.20–7.33 (m, 2H, ArH), 7.40–7.55 (m, 1H, ArH), 7.60–7.72 (m, 2H, ArH), 7.80–7.93(m, 2H, ArH), 7.95–8.18 (m, 2H, ArH), 8.61–8.68 (m, 2H, ArH), 9.15 (bs, 1H, NH; exchangeable with D_2_O), 10.54 (bs, 1H, NH; exchangeable with D_2_O), 11.03 (bs, 1H, NH; exchangeable with D_2_O). Anal. Calcd for C_32_H_30_BrN_5_O_3_S_2_ (676.65): C, 56.80; H, 4.47; N, 10.35 Found: C, 56.62; H, 4.26; N, 10.54.

*4-(3-(4-Chlorophenyl)-5-(6,6-dimethyl-4-oxo-4,5,6,7-tetrahydro-1H-indol-2-yl)-4,5-dihydro-1H-pyrazol-1-yl)-N-(phenylcarbamothioyl)benzenesulfonamide* (**21**). Brown crystals; yield 2.49 g, 78.9%; m.p. 168–169 °C; IR (KBr): 1086 (C=S), 1160, 1355 (SO_2_), 1653 (C=O), 3434 cm^−1^ (NH); ^1^H-NMR (DMSO-*d*_6_) δ 1.16 (s, 6H, 2 CH_3_), 2.71 (s, 2H, CH_2_), 2.87 (s, 2H, CH_2_), 3.89–4.07 (m, 1H, H_4_-pyrazoline), 4.40–4.50 (m, 1H, H_4'_-pyrazoline), 6.90–6.94 (m, 1H, H_5_-pyrazoline), 7.02 (s, 1H, CH-pyrrole), 7.11–7.23 (m, 3H, ArH), 7.40–7.43 (m, 4H, ArH), 7.90–7.94 (m, 4H, ArH), 8.66–8.73 (m, 2H, ArH), 12.41 (bs, 2H, 2NH; exchangeable with D_2_O), 12.73 (bs, 1H, NH; exchangeable with D_2_O). Anal. Calcd for C_32_H_30_ClN_5_O_3_S_2_ (632.20): C, 60.79; H, 4.78; N, 11.08 Found: C, 60.56; H, 4.62; N, 10.87.

### 3.10. 5H-[1,2,4]Triazino[5,6-b]indole-3-thiol ***(22)***

A mixture of indoline-2,3-dione [69,70] (**1**'', 0.1 mol), thiosemicarbazide (0.11 mol) and anhydrous potassium carbonate (0.15 mol) [71] were stirred in water (500 mL) for 2 h at R.T., and then refluxed for 5 h. The mixture was cooled, filtered, and the filtrate was acidified with acetic acid. The solid mass obtained was separated by filtration, washed with water and dried. The product was recrystallized from ethanol. The product was obtained as yellow crystals; yield 19.29 g, 95.4%; m.p. 334–335 °C; IR (KBr): 3423 cm^−1^ (NH); ^1^H-NMR (DMSO-*d*_6_) δ 7.27 (t, 1H, ArH; *J* = 7.6 Hz), 7.37 (d, 1H, ArH; *J* = 7.6 Hz), 7.55 (t, 1H, ArH; *J* = 7.6 Hz), 7.92 (d, 1H, ArH; *J* = 7.6 Hz), 12.43 (bs, 1H, NH; exchangeable with D_2_O), 14.54 (bs, 1H, NH; exchangeable with D_2_O); ^13^C-NMR (DMSO*-d*_6_) δ:113.5, 118.1, 122.3, 123.3, 132.3, 136.1, 143.5, 149.6, 179.5. Anal. Calcd for C_9_H_6_N_4_S (202.24): C, 53.45; H, 2.99; N, 27.70 Found: C, 53.60; H, 3.23; N, 27.58.

### 3.11. 5,10-Dihydro-[1,2,4]triazino[5,6-b]quinoxaline-3-thiol ***(23)***

A mixture of quinoxaline-2,3(1*H*,4*H*)-dione (**1**''', 0.01 mol), thiosemicarbazide (0.011 mol) and anhydrous potassium carbonate (0.015 mol) [71] was stirred in water (500 mL) for 2 h at R.T., and then refluxed for 5 h. The mixture was cooled, filtered, and the filtrate was acidified with acetic acid. The solid mass obtained was separated by filtration, washed with water and dried. The product was recrystallized from ethanol. The product was obtained as off white crystals; yield 1.81 g, 83.6%; m.p. >300 °C; IR (KBr): 3160, 3440 cm^−1^ (NH); ^1^H-NMR (DMSO-*d*_6_) δ 7.03 (d, 2H, ArH; *J* = 7.6 Hz), 7.08 (t, 2H, ArH; *J* = 7.6 Hz), 11.88 (bs, 2H, 2NH; exchangeable with D_2_O), 14.50 (bs, 1H, NH; exchangeable with D_2_O). Anal. Calcd for C_9_H_7_N_5_S (217.25): C, 49.76; H, 3.25; N, 32.24 Found: C, 49.90; H, 3.40; N, 32.00.

### 3.12. 3-Hydrazinyl-5H-[1,2,4]triazino[5,6-b]indole ***(24)***

A mixture of 5*H*-[1,2,4]triazino[5,6-b] indole-3-thiol (**22**, 0.01 mol) [71] and hydrazine hydrate (10 mL, 98%) was heated on water bath for 5 h. The product was collected, washed with ethanol and dried. It was recrystallized from ethanol. The product was obtained as yellow crystals; yield 1.85 g, 92.6%; m.p. 260–261 °C; IR (KBr): 3176, 3242, 3300, 3404 cm^−1^ (NH, NH_2_); ^1^H-NMR (DMSO-*d*_6_) δ 4.31 (bs, 2H, NH_2_; exchangeable with D_2_O), 7.24 (t, 1H, ArH; *J* = 7.9 Hz), 7.38 (d, 1H, ArH; *J* = 7.9 Hz), 7.44 (t, 1H, ArH; *J* = 7.9 Hz), 8.07 (d, 1H, ArH; *J *= 7.9 Hz), 8.54 (bs, 1H, NH; exchangeable with D_2_O), 11.82 (bs, 1H, NH; exchangeable with D_2_O). Anal. Calcd for C_9_H_8_N_6_ (200.20): C, 53.99; H, 4.03 N, 41.98 Found: C, 54.10; H, 4.22; N, 41.80.

### 3.13. 3-Hydrazinyl-5,10-dihydro-[1,2,4]triazino[5,6-b]quinoxaline ***(25)***

A mixture of 5,10-dihydro-[1,2,4]triazino[5,6-*b*]quinoxaline-3-thiol (**23**, 0.01 mol) and hydrazine hydrate (10 mL, 98%) [71] was heated on water bath for 5 h. The product was collected, washed with ethanol, dried and recrystallized from ethanol. The product was obtained as buff crystals; yield 1.83 g, 85.2%; m.p. 255–256 °C; IR (KBr): 3176, 3237, 3271, 3300, 3402 cm^−1^ (NH, NH_2_); ^1^H-NMR (DMSO-*d*_6_) δ 4.55 (bs, 2H, NH_2_; exchangeable with D_2_O), 7.07 (t, 2H, ArH; *J* = 7.6 Hz), 7.29 (d, 2H, ArH; *J* = 7.6 Hz), 8.90 (bs, 1H, NH; exchangeable with D_2_O),11.89 (bs, 2H, 2 NH; exchangeable with D_2_O). Anal. Calcd for C_9_H_9_N_7_ (215.21): C, 50.23; H, 4.22; N, 45.56 Found: C, 50.40; H, 4.45; N, 45.39.

### 3.14. Ethyl [1,2,4]triazolo[3,4-c][1,2,4]triazino[5,6-b]-5H-indole-5-ethanoate ***(26)***

A mixture of 3-hydrazinyl-5*H*-[1,2,4]triazino[5,6-b]indole (**24**, 0.01 mol) [70] and diethyl malonate (20 mL) [72] was heated 10 h. The product was filtered off, washed with ethanol and dried. It was recrystallized from ethanol. The product was obtained as buff crystals; yield 2.55g, 86.3%; m.p. 283–284 °C; IR (KBr): 1737 (C=O), 3408 cm^−1^ (NH); ^1^H-NMR (DMSO-*d*_6_) δ 1.16 (t, 3H, CH_3_-ester; *J* = 6.7 Hz), 4.12 (s, 2H, CH_2_), 4.31 (q, 2H, CH_2_-ester; *J* = 6.7 Hz), 7.20–7.26 (m, 1H, ArH), 7.36–7.38 (m, 1H, ArH), 7.60–7.65 (m, 1H, ArH), 8.00–8.10 (m, 1H, ArH), 12.18 (bs, 1H, NH; exchangeable with D_2_O). Anal. Calcd for C_14_H_12_N_6_O_2_ (296.28): C, 56.75; H, 4.08; N, 28.36 Found: C, 56.90; H, 4.24; N, 28.21.

### 3.15. Ethyl [1,2,4]triazolo[3,4-c][1,2,4]triazino[5,6-b]-5,10-dihydroquinoxaline-5-ethanoate ***(27)***

A mixture of 3-hydrazinyl-5,10-dihydro-[1,2,4]triazino[5,6-b]quinoxaline (**25**, 0.01 mol) and diethyl malonate (20 mL) [72] was heated 10 h. The product was filtered off, washed with ethanol, dried and recrystallized from ethanol. The product was obtained as brown crystals; yield 2.77 g, 89.0%; m.p. 280–281 °C; IR (KBr): 1713 (C=O), 3150, 3430 cm^− ^(NH); ^1^H-NMR (DMSO-d_6_) δ 1.09 (t, 3H, CH_3_; J = 6.9 Hz), 4.09 (q, 2H, CH_2_; J = 6.9 Hz), 4.66 (s, 2H, CH_2_), 7.24 (d, 1H, ArH; J = 8.4 Hz), 7.37–7.41 (m, 2H, ArH), 7.74 (d, 1H, ArH; J = 8.4 Hz), 12.10 (bs, 2H, 2 NH; exchangeable with D_2_O). ^13^C-NMR (DMSO-d_6_) δ: 14.4, 34.5, 62.0, 123.7, 128.5, 129.7, 145.2, 147.0, 152.1, 168.4. Anal. Calcd for C_14_H_13_N_7_O_2_ (311.30): C, 54.02; H, 4.21; N, 31.50 Found: C, 54.25; H, 4.41; N, 31.38.

### 3.16. [1,2,4]Triazolo[3,4-c][1,2,4]triazino[5,6-b]-5H-indole-5-ethanoic acid hydrazide ***(28)***

A solution of ester **26** (0.01 mol) in ethanol (30 mL) and hydrazine hydrate 98% (10 mL) [72] was refluxed for 6 h. The product was collected, washed with ethanol and dried. It was recrystallized from ethanol. The product was obtained as buff crystals; yield 2.15 g, 76.5%; m.p. >300 °C, IR (KBr): 1657 (C=O), 3176, 3242, 3308, 3434 cm^−1^ (NH, NH_2_); ^1^H-NMR (DMSO-*d*_6_) δ 4.24 (s, 2H, CH_2_), 7.24–7.30 (m, 1H, ArH), 7.36–7.41 (m, 1H, ArH), 7.62–7.68 (m, 1H, ArH), 8.03–8.10 (m, 1H, ArH), 9.20 (bs, 1H, NH), 9.89 (bs, 2H, NH_2_; exchangeable with D_2_O), 12.18 (bs, 1H, NH; exchangeable with D_2_O). Anal. Calcd for C_12_H_10_N_8_O (282.26): C, 51.06; H, 3.57; N, 39.70 Found: C, 51.20; H, 4.11; N, 39.48.

### 3.17. [1,2,4]Triazolo[3,4-c][1,2,4]triazino[5,6-b]-5,10-dihydroquinoxaline-5-ethanoic acid hydrazide ***(29)***

A solution of ester **27** (0.01 mol) in ethanol (30 mL) and hydrazine hydrate 98% (10 mL) [72] was refluxed for 6 h. The product was collected, washed with ethanol, dried and recrystallized from ethanol. The product was obtained as orange crystals; yield 2.36 g, 79.6%; m.p. 250–251 °C; IR (KBr): 1675 (C=O), 3190, 3306, 3434 cm^−(^ (NH, NH_2_); ^1^H-NMR (DMSO-*d*_6_) δ 4.34 (s, 2H, CH_2_), 7.24 (t, 1H, ArH; *J* = 7.6 Hz), 7.38 (d, 1H, ArH; *J* = 7.6 Hz), 7.44 (t, 1H, ArH; *J* = 7.6 Hz), 7.87 (d, 1H, ArH; *J* = 7.6 Hz), 8.90 (bs, 1H, NH; exchangeable with D_2_O), 9.61 (bs, 2H, NH_2_; exchangeable with D_2_O), 12.08 (bs, 2H, 2 NH; exchangeable with D_2_O). Anal. Calcd for C_12_H_11_N_9_O (297.28): C, 48.48; H, 3.73; N, 42.41 Found: C, 48.63; H, 3.90; N, 42.30.

### 3.18. [1,2,4]Triazolo[3,4-c][1,2,4]triazino[5,6-b]-5H-indole-5-N-(phenylcarbamothioyl) ethanoic acid hydrazide ***(30)***

A mixture of acid hydrazide **28** (0.01 mol) and anhydrous potassium carbonate (0.05 mol) [71] in absolute ethanol (25 mL) was treated by drop wise addition of phenyl isothiocyanate (0.2 mol) in dry acetone (10 mL) .The reaction mixture was heated under reflux for 12 h. The product was collected, washed with ethanol and dried. The acetone was distilled under reduced pressure and the solid residue was dissolved in water, the product was isolated after acidification with 2 N HCl. The solid mass obtained was separated by filtration, washed with water, dried and recrystallized from ethanol The product was obtained as orange crystals; yield 3.34 g, 80.1%; m.p. 270–271 °C; IR (KBr): 1105 (C=S), 1651 (C=O), 3236, 3433 cm^−1^ (NH); ^1^H-NMR (DMSO-*d*_6_) δ 2.06 (s, 2H, CH_2_), 6.89–6.92 (m, 2H, ArH, NH), 7.24–7.28 (m, 4H, ArH), 7.50–7.53 (m, 4H, ArH), 9.88 (bs, 2H, NH; exchangeable with D_2_O), 11.82 (bs, 1H, NH; exchangeable with D_2_O); ^13^C-NMR (DMSO*-d*_6_) δ: 31.4, 117.5, 117.5, 117.6, 117.6, 121.7, 121.7, 122.1, 123.6, 127.2, 129.6, 129.7, 141.9, 155.0, 156.7, 178.7, 189.4. Anal. Calcd for C_19_H_15_N_9_OS (417.45): C, 54.67; H, 3.62; N, 30.20 Found: C, 54.40; H, 3.43; N, 30.42.

### 3.19. [1,2,4]Triazolo[3,4-c][1,2,4]triazino[5,6-b]-5,10-dihydroquinoxaline-5-N-(phenylcarbamothioyl)-ethanoic acid hydrazide ***(31)***

A mixture of acid hydrazide **35** (0.001 mol) and anhydrous potassium carbonate (0.005 mol) in absolute ethanol (25 mL) was treated by dropwise addition of phenyl isothiocyanate (0.02 mol) [72] in dry acetone (10 mL) .The reaction mixture was heated under reflux for 12 h. The product was collected, washed with ethanol and dried. It was recrystallized from ethanol. The acetone was distilled under reduced pressure and the solid residue was dissolved in water, the product was isolated after acidification with 2 N HCl. The solid mass obtained was separated by filtration, washed with water, dried and recrystallized from ethanol. The product was obtained as buff crystals; yield 0.34 g, 80.0%; m.p. 275–276 °C; IR (KBr): 1109 (C=S), 1651 (C=O), 3236, 3433 cm^−1^ (NH); ^1^H-NMR (DMSO-*d*_6_) δ 4.25 (s, 2H, CH_2_), 7.00–7.54 (m, 5H, 4ArH, NH), 7.56–8.00 (m, 7H, 5ArH, 2NH), 12.05 (m, 2H, 2NH; exchangeable with D_2_O). Anal. Calcd for C_19_H_16_N_10_OS (432.46): C, 52.77; H, 3.73; N, 32.39 Found: C, 52.49; H, 3.53; N, 32.52.

### 3.20. 1-(5H-[1,2,4]Triazino[5,6-b]indol-3-yl)-3-methyl-1H-pyrazol-5(4H)-one ***(32)***

A mixture of 3-hydrazinyl-5*H*-[1,2,4]triazino[5,6-b]indole (**24**, 0.01 mol) and ethyl acetoacetate (0.011 mol) [73] in acetic acid (20 mL) was stirred with refluxing for 4 h. The reaction mixture was evaporated till dryness, the product was collected, washed with ethanol and dried. It was recrystallized from ethanol. The product was obtained as buff crystals; yield 2.47 g, 93.0%; m.p. 330–331 °C; IR (KBr): 1712 (C=O), 3128, 3438 cm^−1^ (NH); ^1^H-NMR (DMSO-*d*_6_) δ 1.80 (s, 3H, CH_3_), 2.43 (s, 2H, CH_2_), 7.45 (t, 1H, ArH; *J* = 6.7 Hz), 7.61 (d, 1H, ArH; *J* = 6.7 Hz), 7.72 (t, 1H, ArH; *J* = 6.7 Hz), 8.36 (d, 1H, ArH; *J* = 6.7 Hz), 12.00 (bs, 1H, NH; exchangeable with D_2_O). Anal. Calcd for C_13_H_10_N_6_O (266.26): C, 58.64; H, 3.79; N, 31.56 Found: C, 58.80; H, 3.90; N, 31.43.

### 3.21. 1-(5,10-Dihydro-[1,2,4]triazino[5,6-b]quinoxalin-3-yl)-3-methyl-1H-pyrazol-5(4H)-one ***(33)***

A mixture of 3-hydrazinyl-5,10-dihydro-[1,2,4]triazino[5,6-*b*]quinoxaline **25** **(** 0.01 mol) and ethyl acetoacetate (0.011 mol) [73] in acetic acid (20 mL) was stirred with refluxing for 4 h. Evaporation till dryness, the product was collected, washed with ethanol and dried. It was recrystallized from ethanol. The product was obtained as buff crystals; yield 2.45 g, 87.2%; m.p. 239–240 °C; IR (KBr): 1693 (C=O), 3161, 3438 cm^−1^ (NH); ^1^H-NMR (DMSO-*d*_6_) δ 2.29 (s, 3H, CH_3_), 2.92 (s, 2H, CH_2_), 7.23 (t, 1H, ArH; *J* = 7.6 Hz), 7.33 (d, 1H, ArH; *J* = 7.6 Hz), 7.39 (t, 1H, ArH; *J* = 7.6 Hz), 7.98 (d, 1H, ArH; *J* = 7.6 Hz), 10.99 (bs, 1H, NH; exchangeable with D_2_O), 11.68 (bs, 1H, NH; exchangeable with D_2_O). ^13^C-NMR (DMSO*-d*_6_) δ: 15.0, 43.8, 127.9, 130.7, 144.5, 152.9, 155.7, 161.8, 170.0. Anal. Calcd for C_13_H_11_N_7_O (281.27): C, 55.51; H, 3.94; N, 34.86 Found: C, 55.68; H, 4.03; N, 34.70.

### 3.22. 1-(5H-[1,2,4]Triazino[5,6-b]indol-3-yl)-3-methyl-4-(propan-2-ylidene)-1H-pyrazol-5(4H)-one ***(34)***

A solution of 1-(5*H*-[1,2,4]triazino[5,6-*b*]indol-3-yl)-3-methyl-1*H*-pyrazol-5(4*H*)-one (**32**, 0.01 mol) in acetone (25 mL) [73] was stirred with refluxing for 20 h. The reaction mixture was evaporated till dryness, cooled. 20 mL of water was added in order to precipitate the product. The crude product was filtered, washed with ethanol, dried, and recrystallized from ethanol. The product was obtained as off white crystals; yield 2.47 g, 80.7%; m.p. 360–361 °C; IR (KBr): 1713 (C=O), 3439 cm^−1^ (NH); ^1^H-NMR (DMSO-*d*_6_) δ 1.87 (s, 6H, 2 CH_3_), 2.49 (s, 3H, CH_3_), 7.44 (t, 1H, ArH; *J* = 7.6 Hz), 7.62 (d, 1H, ArH; *J* = 7.6 Hz), 7.71 (t, 1H, ArH; *J* = 7.6 Hz), 8.36 (d, 1H, ArH; *J* = 7.6 Hz), 13.08 (bs, 1H, NH; exchangeable with D_2_O). Anal. Calcd for C_16_H_14_N_6_O (306.32): C, 62.74; H, 4.61; N, 27.44 Found: C, 62.97; H, 5.03; N, 27.70.

### 3.23. 1-(5,10-Dihydro-[1,2,4]triazino[5,6-b]quinoxalin-3-yl)-3-methyl-4-(propan-2-ylidene)-1H-pyrazol-5(4H)-one ***(35)***

A solution of 1-(5,10-dihyro-[1,2,4]triazino[5,6-*b*]quinoxalin-3-yl)-3-methyl-1*H*-pyrazol-5(4*H*)-one (**33**, 0.01 mol) in acetone (25 mL) [73] was stirred with refluxing for 20 h. Evaporation till dryness, cooled, water was added, filter the product was collected, washed with ethanol and dried. It was recrystallized from ethanol. The product was obtained as buff crystals; yield 2.66 g, 82.9%; crystals; m.p. 263–264 °C; IR (KBr): 1701 (C=O), 3202, 3433 cm^−1^ (NH); ^1^H-NMR (DMSO-*d*_6_) δ 2.30 (s, 3H, CH_3_), 2.42 (s, 3H, CH_3_), 2.93 (s, 3H, CH_3_), 7.00–7.05 (m, 2H, ArH), 7.35–7.39 (m, 2H, ArH), 11.88 (bs, 2H, 2 NH; exchangeable with D_2_O); ^13^C-NMR (DMSO-*d*_6_) δ:14.6, 19.2, 107.3, 115.6, 123.5, 126.1, 137.4, 153.0, 155.7, 159.5, 161.8. Anal. Calcd for C_16_H_15_N_7_O (321.34): C, 59.80; H, 4.71; N, 30.51 Found: C, 60.03; H, 4.93; N, 30.37.

### 3.24. 2-((2-(5H-[1,2,4]Triazino[5,6-b]indol-3-yl)hydrazono)methyl)-6,6-dimethyl-6,7-dihydro-1H-indol-4(5H)-one ***(36)***

A mixture of 3-hydrazinyl-5*H*-[1,2,4]triazino[5,6-*b*]indole (**24**, 0.001 mol) and 6,6-dimethyl-4-oxo-4,5,6,7-tetrahydro-1*H*-indole-2-carbaldehyde (**1**', 0.0011 mol) was refluxed in ethanol (30 mL) for 6 h. The product was collected, washed with ethanol and dried, it was recrystallized from ethanol. The product was obtained as brown crystals; yield 0.32 g, 87.6%; m.p. 290–291 °C; IR (KBr): IR (KBr): 1652 (C=O), 3234, 3439 cm^−1^ (NH); ^1^H-NMR (DMSO-*d*_6_) δ 1.00 (s, 6H, 2 CH_3_), 2.21 (s, 2H, CH_2_), 2.68 (s, 2H, CH_2_), 6.53 (s, 1H, CH-pyrrole), 7.30 (t, 1H, ArH; *J* = 7.9 Hz), 7.46 (d, 1H, ArH; *J* = 7.9 Hz ), 7.51 (t, 1H, ArH; *J* = 7.9 Hz), 8.03 (s, 1H, CH=N), 8.13 (d, 1H, ArH; *J* = 7.9 Hz), 8.34 (bs, 1H, NH; exchangeable with D_2_O), 11.52 (bs, 1H, NH; exchangeable with D_2_O), 11.89 (bs, 1H, NH; exchangeable with D_2_O). ^13^C-NMR (DMSO*-d*_6_) δ: 28.7, 35.7, 36.6, 52.2, 108.2, 112.7, 119.4, 119.9, 120.6, 122.5, 129.4, 129.6, 136.1, 138.6, 140.3, 146.3, 148.8, 158.7, 192.6. Anal. Calcd for C_20_H_19_N_7_O (373.41): C, 64.33; H, 5.13; N, 26.26 Found: C, 64.63; H, 4.90; N, 26.30.

### 3.25. 2-((2-(5,10-Dihydro-[1,2,4]triazino[5,6-b]quinoxalin-3-yl)hydrazono)methyl)-6,6-dimethyl-6,7-dihydro-1H-indol-4(5H)-one ***(37)***

A mixture of 3-hydrazinyl-5,10-dihydro-[1,2,4]triazino[5,6-*b*] quinoxaline (**25**, 0.001 mol) and 6,6-dimethyl-4-*oxo*-4,5,6,7-tetrahydro-1*H*-indole-2-carbaldehyde (**1a**, 0.0011 mol) was refluxed in ethanol (30 mL) for 6 h. The product was collected, washed with ethanol, dried and recrystallized from ethanol. The product was obtained as yellow crystals; yield 0.33 g, 86.6%; m.p. 155–156 °C; IR (KBr): 1651 (C=O), 3236, 3433 cm^−1^ (NH); ^1^H-NMR (DMSO-*d*_6_) δ 0.97 (s, 3H, CH_3_), 1.00 (s, 3H, CH_3_), 2.21 (s, 2H, CH_2_), 2.69 (s, 2H, CH_2_), 6.82 (s, 1H, CH-pyrrole), 7.03–7.06 (m, 2H, ArH), 7.07–7.10 (m, 2H, ArH), 9.45 (s, 1H, CH=N), 10.34 (bs, 1H, NH; exchangeable with D_2_O), 11.16 (bs, 1H, NH; exchangeable with D_2_O), 11.91 (bs, 1H, NH; exchangeable with D_2_O), 12.11 (bs, 1H, NH; exchangeable with D_2_O). Anal. Calcd for C_20_H_20_N_8_O (388.43): C, 61.84; H, 5.19; N, 28.85 Found: C, 62.00; H, 5.31; N, 28.70.

### 3.26. 3-(5H-[1,2,4]Triazino[5,6-b]indol-3-yl)-2-(6,6-dimethyl-4-oxo-4,5,6,7-tetrahydro-1H-indol-2-yl)thiazolidin-4-one ***(38)***

A mixture of 2-((2-(5*H*-[1,2,4]triazino[5,6-*b*]indol-3-yl)hydrazono)methyl)-6,6-dimethyl-6,7-dihydro-1*H*-indol-4(5*H*)-one (**36**, 0.001 mol) and thioglycolic acid (0.0012 mol) was refluxed in dry benzene (30 mL) on water bath for 10 h, cooled and poured onto water. The reaction mixture was extracted with benzene three times, washed with sodium bicarbonate, water, dried over anhydrous sodium sulfate, then concentrated to half its volume. The separated solid product was filtered, washed ethanol, dried and recrystallized from ethanol. The product was obtained as buff crystals; yield 0.33 g, 77.2%; m.p. 215–216 °C; IR (KBr): 1650, 1710 (C=O), 3427 cm^−1^ (NH); ^1^H-NMR (DMSO-*d*_6_) δ 1.00 (s, 6H, 2 CH_3_), 2.20 (s, 2H, CH_2_), 2.69 (s, 2H, CH_2_), 3.35–3.49 (m, 2H, CH_2_ –thiazol.), 6.52 (s, 1H, CH-pyrrole), 7.24 (t, 1H, ArH; *J* = 7.7 Hz), 7.43–7.49 (m, 2H, ArH), 7.93 (s, 1H, H-thiazol.), 8.08 (d, 1H, ArH; *J* = 7.7 Hz), 11.46 (bs, 1H, NH; exchangeable with D_2_O), 12.03 (bs, 1H, NH; exchangeable with D_2_O). Anal. Calcd for C_22_H_20_N_6_O_2_S (432.50): C, 61.10; H, 4.66; N, 19.43 Found: C, 61.36; H, 4.86; N, 19.21.

### 3.27. N'-2-((2-(5,10-Dihydro-[1,2,4]triazino[5,6-b]quinoxalin-3-yl)hydrazono)methyl)-6,6-dimethyl-6,7-dihydro-1H-indol-4(5H)-ylidene)benzohydrazide ***(39)***

To a solution of 2-((2-(5,10-dihydro-[1,2,4]triazino[5,6-*b*]quinoxalin-3-yl)hydrazono)methyl)-6,6-dimethyl-6,7-dihydro-1*H*-indol-4(5*H*)-one (**37**, 0.001 mol) in ethanol (30 mL) was added benzoyl hydrazine (0.163 g, 0.0012 mol) and two drops of acetic acid. The reaction mixture was heated under reflux for 6 h, partially concentrated and cooled. The separated solid product was filtered, washed with ethanol, dried and recrystallized from ethanol. The product was obtained as yellow crystals; yield 0.40 g, 79.7%; m.p. 175–176 °C; IR (KBr): 1644 (C=O), 3236, 3426 cm^−1^ (NH); ^1^H-NMR (DMSO-*d*_6_) δ 1.01 (s, 6H, 2 CH_3_), 2.22 (s, 2H, CH_2_), 2.69 (s, 2H, CH_2_), 6.66 (s, 1H, CH-pyrrole), 7.17 (bs, 1H, NH; exchangeable with D_2_O), 7.49–7.51 (m, 5H, ArH), 7.87–7.90 (m, 4H, ArH), 8.25 (s, 1H, CH=N), 10.56 (s, 1H, NH; exchangeable with D_2_O), 11.55 (s, 1H, NH; exchangeable with D_2_O), 11.63 (s, 1H, NH; exchangeable with D_2_O), 12.00 (s, 1H, NH; exchangeable with D_2_O); ^13^C-NMR (DMSO-*d*_6_) δ: 28.8, 29.0, 35.8, 36.7, 110.6, 112.3, 120.2, 128.2, 128.3, 128.3, 128.3, 129.2, 129.2, 132.3, 132.6, 140.2, 146.6, 148. 3, 158.4, 172.7. Anal. Calcd for C_27_H_26_N_10_O (506.56): C, 64.02; H, 5.17; N, 27.65 Found: C, 63.89; H, 4.92; N, 27.80.

### 3.28. 3-(6,6-Dimethyl-6,7-dihydro-1H-indol-4(5H)-one)-1,5-dihydro-[1,2,4]triazolo[3,4-c]-5,10-dihydro-[1,2,4]triazino[5,6-b]quinoxaline ***(40)***

A mixture of 2-((2-(5,10-dihydro-[1,2,4]triazino*[5,6-b]*quinoxalin-3-yl)hydrazono)methyl)-6,6-dimethyl-6,7-dihydro-*1H*-indol-4(5*H*)-one (**37**, 0.001 mol) and acetic anhydride (15 mL) was heated on a boiling water bath for 10 h. The reaction mixture was poured onto crushed ice, the precipitated product was filtered, washed with water, dried and recrystallized from ethanol/chloroform. The product was obtained as brown crystals; yield 0.28 g, 73.5%; m.p. 280–281 °C; IR (KBr): 1662 (C=O), 3234, 3429 cm^−1^ (NH); ^1^H-NMR (DMSO-*d*_6_) δ 1.08 (s, 6H, 2 CH_3_), 2.32 (s, 2H, CH_2_), 2.75 (s, 2H, CH_2_), 6.79 (s, 1H, CH-pyrrole), 7.09–7.12 (m, 1H, ArH), 7.31 (d, 1H, ArH; *J* = 7.6 Hz), 7.38–7.42 (m, 2H, ArH), 12.23 (bs, 3H, 3NH; exchangeable with D_2_O). Anal. Calcd for C_20_H_18_N_8_O(386.41): C, 62.17; H, 4.70; N, 29.00 Found: C, 61.92; H, 4.62; N, 29.19.

### 3.29. 2-(1-(5H-[1,2,4]Triazino[5,6-b]indol-3-yl)-3-(4-bromophenyl)-1H-pyrazol-5-yl)-(6,6-dimethyl-6,7-dihydro-1H-indol-4(5H)-one ***(41)***

To a solution of 2-(3-(4-bromophenyl)-3-oxoprop-1-enyl)-6,6-dimethyl-6,7-dihydro-1*H*-indol-4(5*H*)-one (**2**, 0.001 mol) in ethanol (15 mL) was added 3-hydrazinyl-5*H*-[1,2,4]triazino[5,6-*b*]indole (**24**, 0.0012 mol) and acetic acid (5 mL). The reaction mixture was heated under reflux for 7 h, concentrated till dryness, poured onto H_2_O (50 mL), 10% bromine water (5 mL) was added, the mixture stirred overnight, and poured onto crushed ice. The separated solid product was filtered, washed with ethanol, dried and recrystallized from ethanol. The product was obtained as brown crystals; yield 0.44 g, 80.8%; m.p. 150–151 °C; IR (KBr): 1649 (C=O), 3439 cm^−1^ (NH); ^1^H-NMR (DMSO-*d*_6_) δ 1.01 (s, 6H, 2 CH_3_), 2.35 (s, 2H, CH_2_), 2.67 (s, 2H, CH_2_), 6.12 (s, 1H, CH-pyrazole), 6.70 (s, 1H, CH-pyrrole), 7.30–7.37 (m, 2H, ArH), 7.50–7.54 (m, 4H, ArH), 7.73 (d, 2H, ArH; *J* = 7.7 Hz), 11.35 (s, 1H, NH; exchangeable with D_2_O), 11.78 (s, 1H, NH; exchangeable with D_2_O); ^13^C-NMR (DMSO-*d*_6_) δ: 28.9, 33.2, 45.4, 57.8, 108.2, 111.3, 113.6, 120.8, 121.8, 122.6, 124.7, 124.9, 125.2, 125.7, 126.9, 127.6, 128.9, 132.3, 142.2, 146.6, 148.5, 150.4, 157.6, 190.8. Anal. Calcd for C_28_H_22_BrN_7_O (552.42): C, 60.88; H, 4.01; N, 17.75 Found: C, 61.00; H, 4.20; N, 17.58.

### 3.30. General Procedure for the Preparation of Compounds ***42–44***

To a solution of 2-(3-aryl-3-oxoprop-1-enyl)-6,6-dimethyl-6,7-dihydro-1*H*-indol-4(5*H*)-one **2**–**4** (0.001 mol) in ethanol (15 mL) was added 3-hydrazinyl-5,10-dihydro-[1,2,4]triazino[*5,6-b*] quinoxaline (**25**, 0.0012 mol) and acetic acid (5 mL). The reaction mixture was heated under reflux for 7 h, concentrated till dryness, poured onto H_2_O (50 mL), 10% bromine water (5 mL) was added, the mixture stirred overnight, and poured onto crushed ice. The separated solid product was filtered, washed with ethanol, dried and recrystallized from ethanol.

*2-(3-(4-Bromophenyl)-1-(5,10-dihydro-[1,2,4]triazino[5,6-b]quinoxalin-3-yl)-1H-pyrazol-5-yl)-6,6-dimethyl-6,7-dihydro-1H-indol-4(5H)-one* (**42**). Orange crystals; yield 0.49 g, 88.0%; m.p. 289–290 °C; IR (KBr): 1680 (C=O), 3232, 3454 cm^−1^ (NH); ^1^H-NMR (DMSO-*d*_6_) δ 1.01 (s, 6H, 2 CH_3_), 2.23 (s, 2H, CH_2_), 2.71 (s, 2H, CH_2_), 6.80 (s, 1H, CH-pyrazole), 6.91 (s, 1H, CH-pyrrole), 7.50–7.60 (m, 4H, ArH), 7.76 (d, 2H, ArH; *J* = 8.4 Hz), 7.92 (d, 2H, ArH; *J* = 8.4 Hz), 12.06 (m, 3H, 3NH; exchangeable with D_2_O); ^13^C-NMR (DMSO-*d*_6_) δ:.28.8, 34.5, 48.6, 49.7, 51.5, 99.1, 115.6, 122.4, 124, 125.8, 129.0, 129.8, 131.8, 138.0, 140.1, 1418, 143.9, 146.6, 151.7, 155.6, 193.3. Anal. Calcd for C_28_H_23_BrN_8_O (567.44): C, 59.27; H, 4.09; N, 19.75 Found: C, 59.15; H, 4.21; N, 19.50.

*2-(3-(4-Chlorophenyl)-1-(5,10-Dihydro-[1,2,4]triazino[5,6-b]quinoxalin-3-yl)-1H-pyrazol-5-yl)-6,6-dimethyl-6,7-dihydro-1H-indol-4(5H)-one* (**43**). Brown crystals; yield 0.48 g, 82.3%; m.p. 229–230 °C; IR (KBr): 1681 (C=O), 3250, 3405 cm^−1^ (NH); ^1^H-NMR (DMSO-*d*_6_) δ 1.00 (s, 6H, 2 CH_3_), 2.23 (s, 2H, CH_2_), 2.71 (s, 2H, CH_2_), 6.90 (s, 1H, CH-pyrazole), 7.27 (s, 1H, CH-pyrrole), 7.50–7.55 (m, 2H, ArH), 7.69–7.72 (m, 4H, ArH), 8.00–8.12 (m, 2H, ArH), 12.18 (bs, 3H, 3NH; exchangeable with D_2_O); ^13^C-NMR (DMSO*-d*_6_) δ:.28.8, 39.8, 40.2, 40.6, 54.2, 64.1, 109.6, 115.8, 126.3, 129.5, 129.6, 129.6, 130.6 130.8, 137.4, 143.2, 143.7, 150.2, 155.9, 169.5, 192.8. Anal. Calcd for C_28_H_23_ClN_8_O (522.99): C, 64.30; H, 4.43; N, 21.43 Found: C, 64.26; H, 4.20; N, 21.66.

*2-(1-(5,10-Dihydro-[1,2,4]triazino[5,6-b]quinoxalin-3-yl)-3-(4-methoxyphenyl)-1H-pyrazol-5-yl)-6,6-dimethyl-6,7-dihydro-1H-indol-4(5H)-one* (**44**). Brown crystals; yield 0.45 g, 86.8%; m.p. 280–281 °C; IR (KBr): 1681 (C=O), 3151, 3436 cm^−1^ (NH); ^1^H-NMR (DMSO*-d*_6_) δ 1.02 (s, 6H, 2 CH_3_), 2.24 (s, 2H, CH_2_), 2.72 (s, 2H, CH_2_), 2.94 (s, 3H, OCH_3_), 6.75–6.78 (m, 1H, CH-pyrazole), 6.85 (s, 1H, CH-pyrrole), 7.06 (d, 2H, ArH; *J* = 8.4 Hz), 7.27–7.36 (m, 2H, ArH), 7.40 (d, 2H, ArH; *J* = 8.4 Hz), 8.01–8.09 (m, 2H, ArH), 11.95 (bs, 2H, 2NH; exchangeable with D_2_O), 12.05 (bs, 1H, NH; exchangeable with D_2_O). ^13^C-NMR (DMSO*-d*_6_) δ: 28.6, 35.0, 43.1, 53.8, 56.1, 100.1, 115.6, 117.1, 117.3, 121.5, 123.5, 123.8, 126.1, 129.5, 135.2, 140.1, 144.7, 149.7, 152.3, 155.7, 160.8, 193.0. Anal. Calcd for C_29_H_26_N_8_O_2_ (518.57): C, 67.17; H, 5.05; N, 21.61 Found: C, 67.30; H, 5.18; N, 21.41.

### 3.31. Biological Activity Assay

#### 3.31.1. Inhibition Zone Measurement (IZ)

Compounds **2**–**44** were evaluated *in vitro* for antimicrobial activity against the following four organisms: *Escherichia coli* ATCC8739, *Pseudomonas aeruginosa* ATCC 9027 as examples of Gram-negative bacteria, *Staphylococcus aureus* ATCC 6583P as an example of Gram-positive bacteria, and *Candida albicans* ATCC 2091 as an example of a yeast-like fungus have been studied by using the Nutrient Agar (NA) and Sabouraud Dextrose Agar (SDA) diffusion methods [74], respectively, in *N,N*-dmethylformamide as solvent. The bacteria were subcultured on Nutrient Agar medium (NA), whereas, fungi were subcultured on Sabouraud Dextrose Agar (SDA). Petri plates (150 mm × 15 mm) were prepared by pouring 60 mL of NA or SDA and allowing it to solidify. Plates were dried and 1 mL of each standardized inoculums suspension was poured and uniformly spread. The excess inoculums was drained and the inoculums was allowed to dry for 15 min. Eight equidistant wells were made in the medium using a sterile cork borer (6 mm in diameter and 75 μL of the test chemicals (1 mg/mL) diluted in DMF were placed into the wells. The plates containing bacterial and fungi species were incubated at 37 °C for 24 h. The tests were carried in triplicate. Ampicillin trihydrate (10.0 µg/disc), ciprofloxacin (5.0 µg/disc), impenam (10.0 µg/disc), and clotrimazole (100.0 µg/disc) were used as standard antibacterial and antifungal agents, respectively. (DMF) alone showed no inhibition zone. The plates were incubated at 37 °C for 24 h. The results were recorded for each tested compound as the average diameter of inhibition zones of bacterial growth around the disks in mm.

#### 3.31.2. Minimal inhibitory concentration (MIC)

MIC measurements [75] were carried out for compounds that showed significant inhibition zones using the twofold serial dilution technique. The compounds **2**–**44** were prepared in a concentration range of 200, 100, 50, 25, and 12.5 µg/mL. The microdilution susceptibility test in Muller-Hinton broth (oxoid) and Sabouraud Liquid Medium (oxoid) were used for the determination of antibacterial and antifungal activity. The microorganism suspensions at 106 CFU/mL (colony forming unit/mL) were used to inoculate the prepared test compounds in the above mentioned serial dilution broth. The culture tubes were incubated at 37 °C for 24–48 h. At the end of the incubation period the growth of bacteria was observed by turbidity measurements [75]. The MIC is defined as the lowest concentration that showed no bacterial growth.

## 4. Conclusions

The objective of the present study was to synthesize and investigate the antimicrobial and antifungal activity of a new series of pyrazolines and pyrazoles in the hope of discovering new structural leads serving as antimicrobial agents. Some new pyrazoline and pyrazole derivatives have been prepared, and their physical properties were characterized. The biological activity of the compounds **2**–**44** was evaluated by the agar diffusion method against *Escherichia coli*, *Pseudomonas aeruginosa*, *Staphylococcus aurous* and *Candida albicans*. None of the investigated compounds not showed any activity against the test organisms *Escherichia coli* and *Pseudomonas aeruginosa*. Compound **16** has good antimicrobial activity against *Staphylococcus aureus*, comparable to that of ampicillin and ciprofloxacin, while compound **17** has remarkable antimicrobial activity against *Staphylococcus aureus*, exceeding that of ampicillin, ciprofloxacin and imipenam. In addition, compound **17** has comparable IZ against *Candida albicans* comparable to that of clotrimazole. On the other hand, the minimal inhibitory concentration (MIC) of compounds **19**, **20** and **31** against *Candida albicans* indicate good antifungal activity, comparable to that of clotrimazole. Based on the preliminary results, it can be seen that all four compounds **16**, **17**, **19** and **20** showing good antimicrobial and antifungal activity have benzenesulfonamide substituents as a common structural feature.

## Supplementary Materials

Supplementary materials can be accessed at: http://www.mdpi.com/1420-3049/18/3/2683/s1.
