# Supplementary material for: Synthesis, Antibacterial and Antifungal Activity of Some New Pyrazoline and Pyrazole Derivatives
Source: Molecules. 2013 Feb 28;18(3):2683–711. doi: 10.3390/molecules18032683 (PMC6270532; doi:10.3390/molecules18032683)

Supplementary Materials

Figure 1. ^{1}H-NMR of compound **2**
Figure 2. ^{1}H-NMR of compound **3**
Figure 3. ^{1}H-NMR of compound **4**
Figure 4. ^{1}H-NMR of compound **11**
Figure 5. ^{1}H-NMR of compound **18**
Figure 6. ^{1}H-NMR of compound **22**
Figure 7. ^{1}H-NMR of compound **24**
Figure 8. ^{1}H-NMR of compound **27**
Figure 9. ^{1}H-NMR of compound **33**
Figure 10. ^{1}H-NMR of compound **34**
Figure 11. ^{1}H-NMR of compound **36**
Figure 12. ^{1}H-NMR of compound **38**
Figure 13. ^{1}H-NMR of compound **40**
Figure 14. ^{13}C-NMR of compound **2**
Figure 15. ^{13}C-NMR of compound **27**
Figure 16. ^{13}C-NMR of compound **35**
Figure 17. ^{13}C-NMR of compound **36**
Figure 18. ^{13}C-NMR of compound **22**

Figure 1. ^{1}H-NMR of compound **2**.

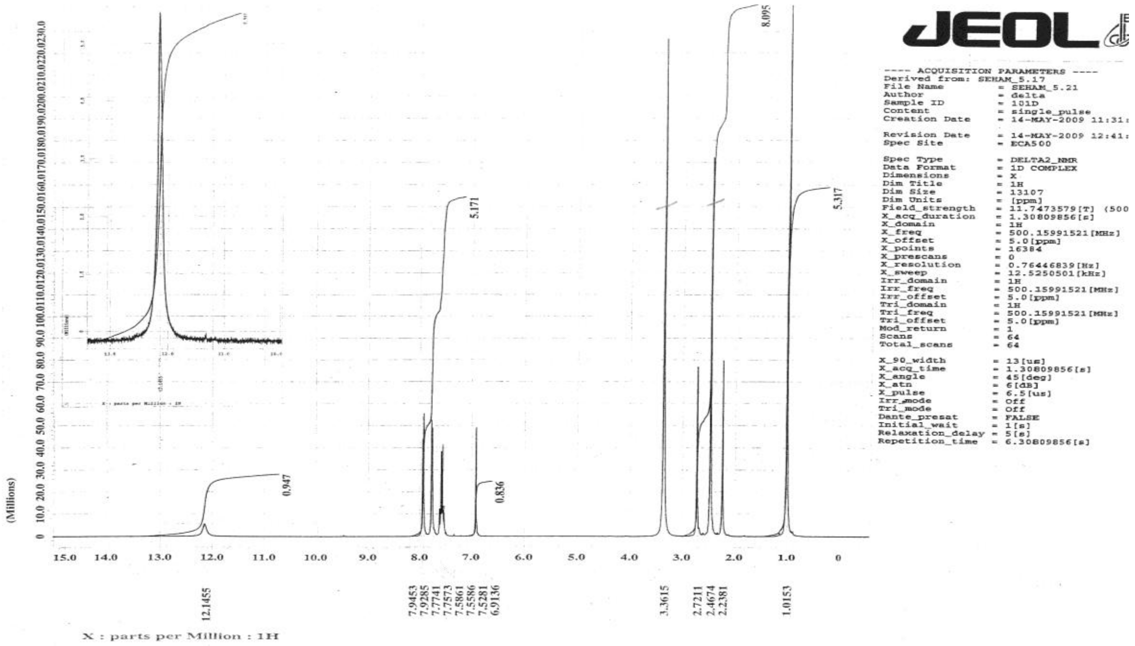

Figure 2. ^{1}H-NMR of compound **3**.

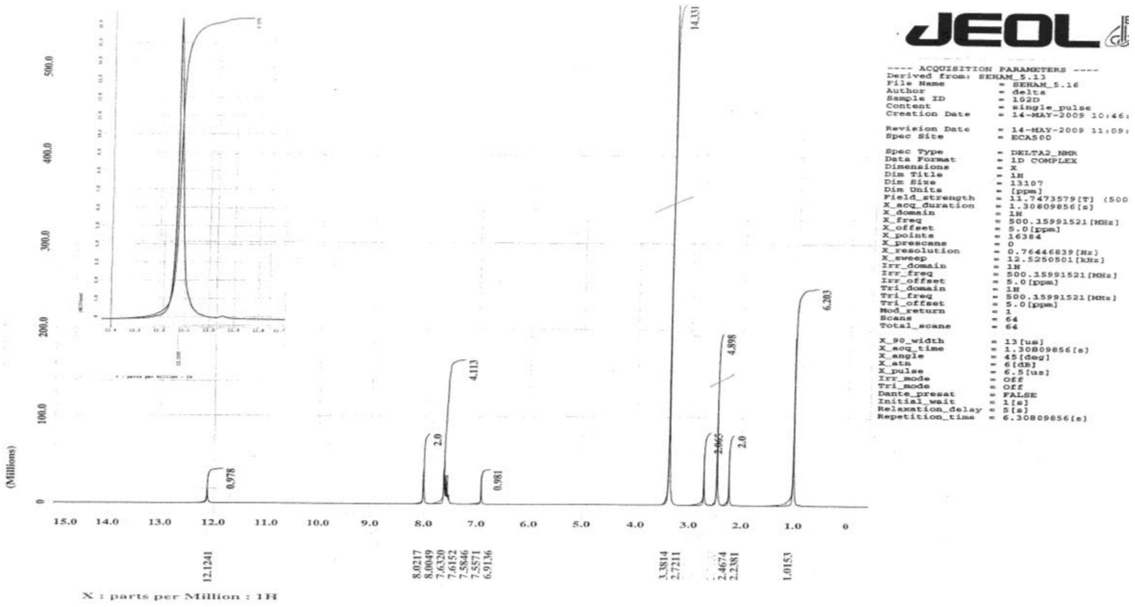

Figure 2. *Cont*.

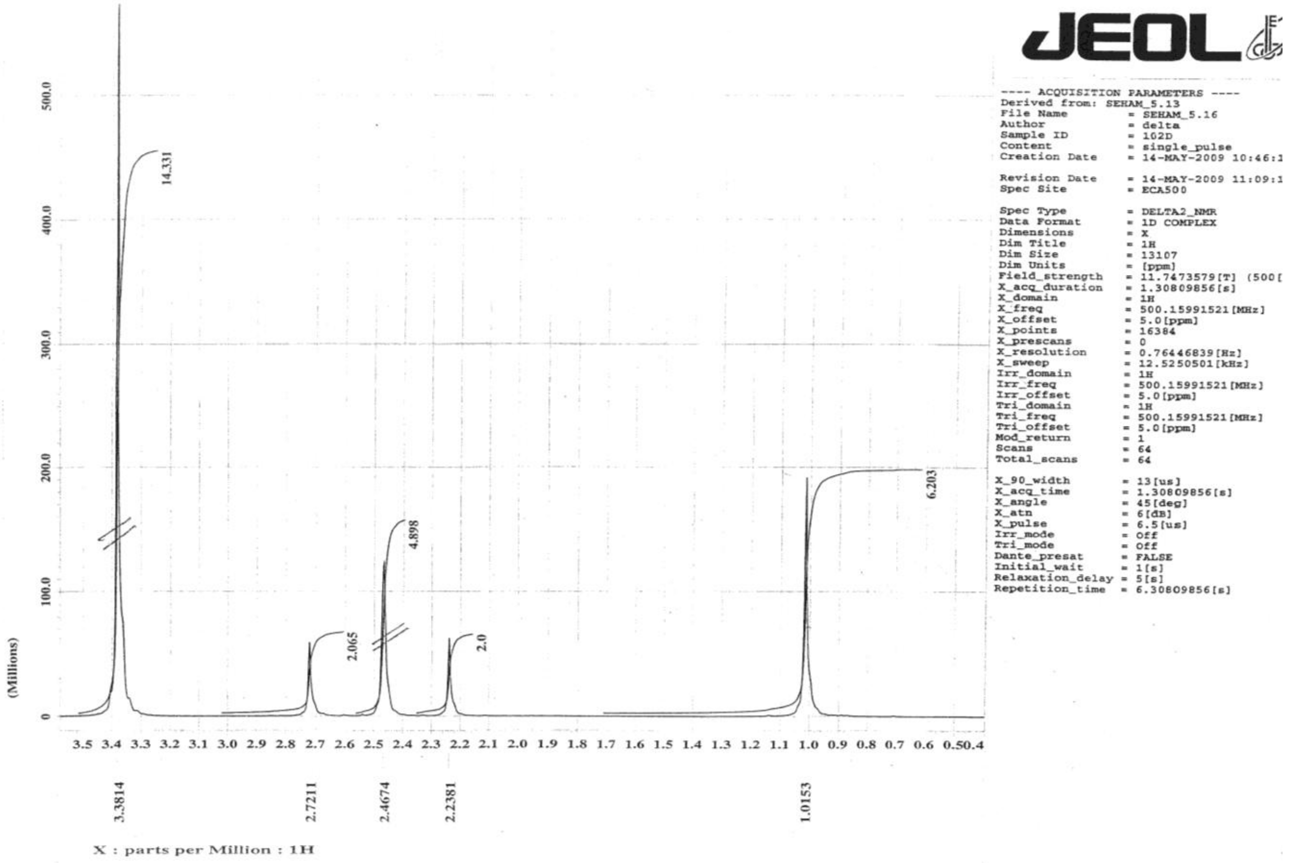

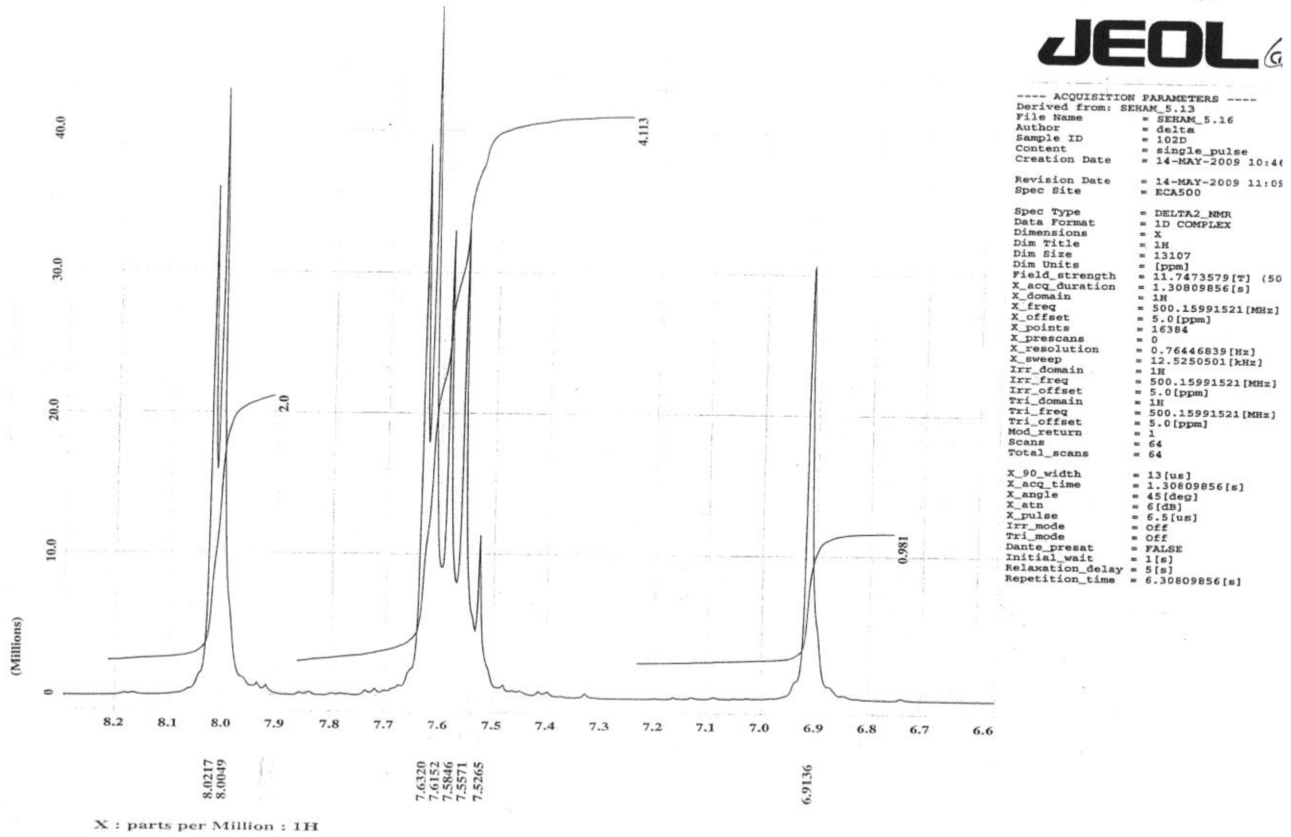

Figure 3. ^{1}H-NMR of compound **4**.

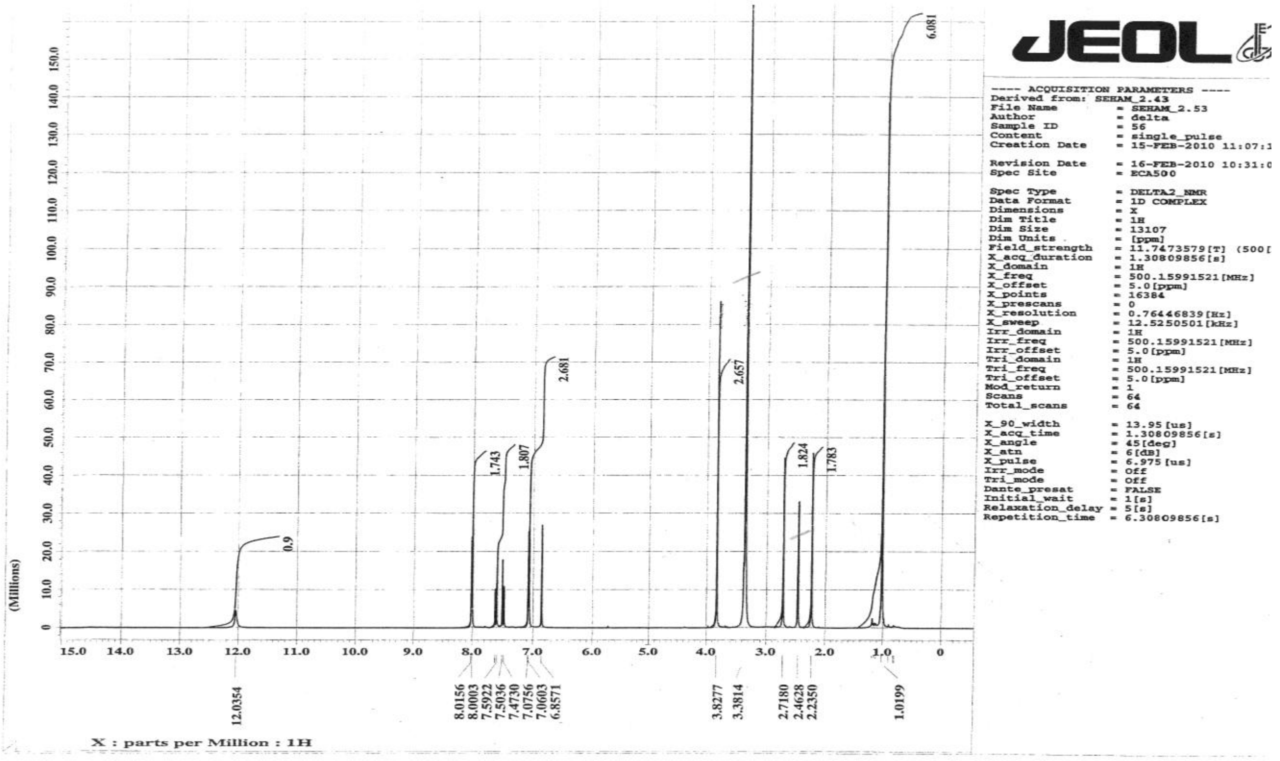

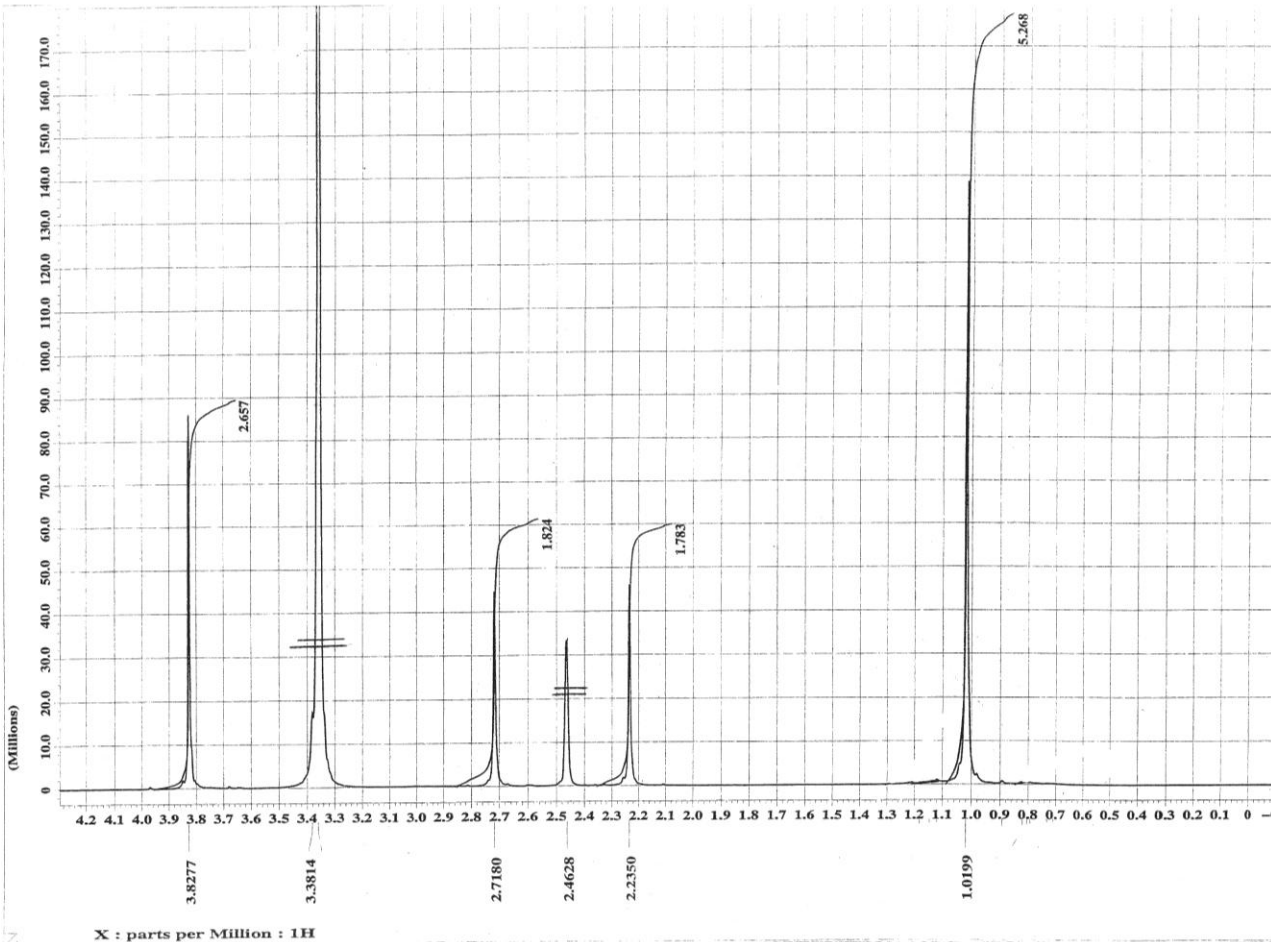

Figure 3. *Cont.*

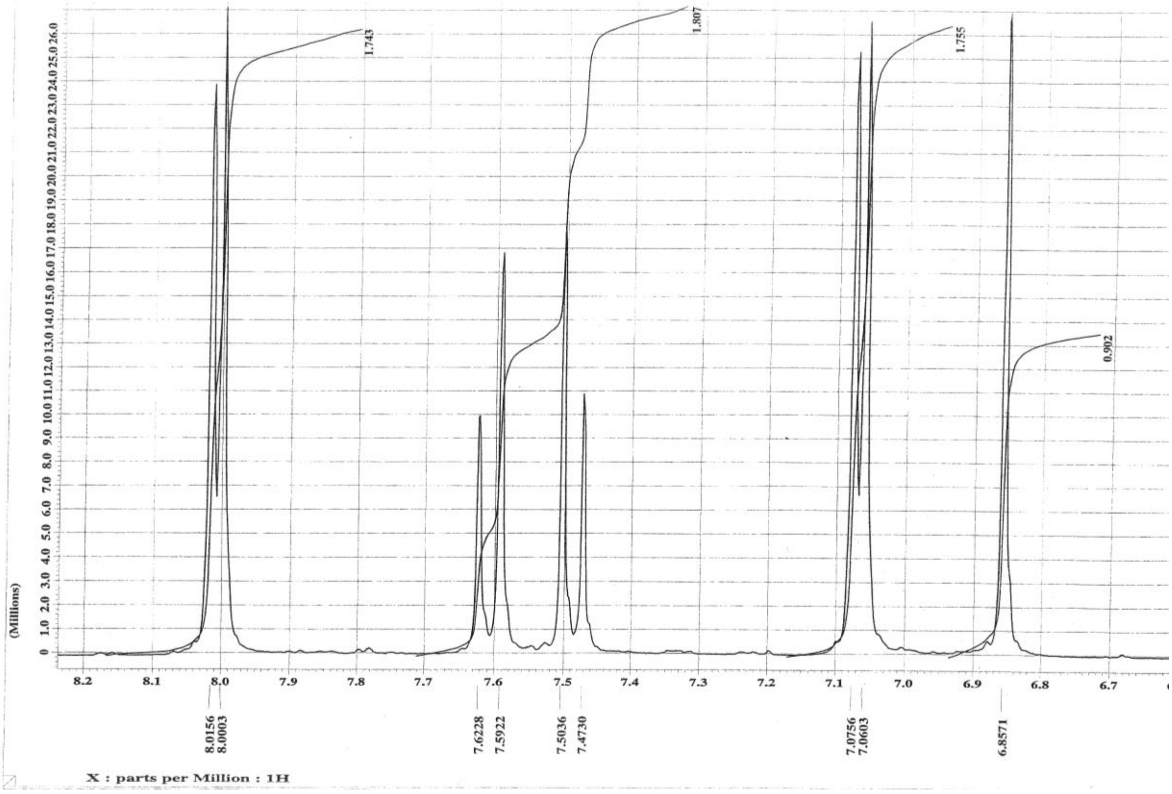

Figure 4. ^{1}H-NMR of compound **11**.

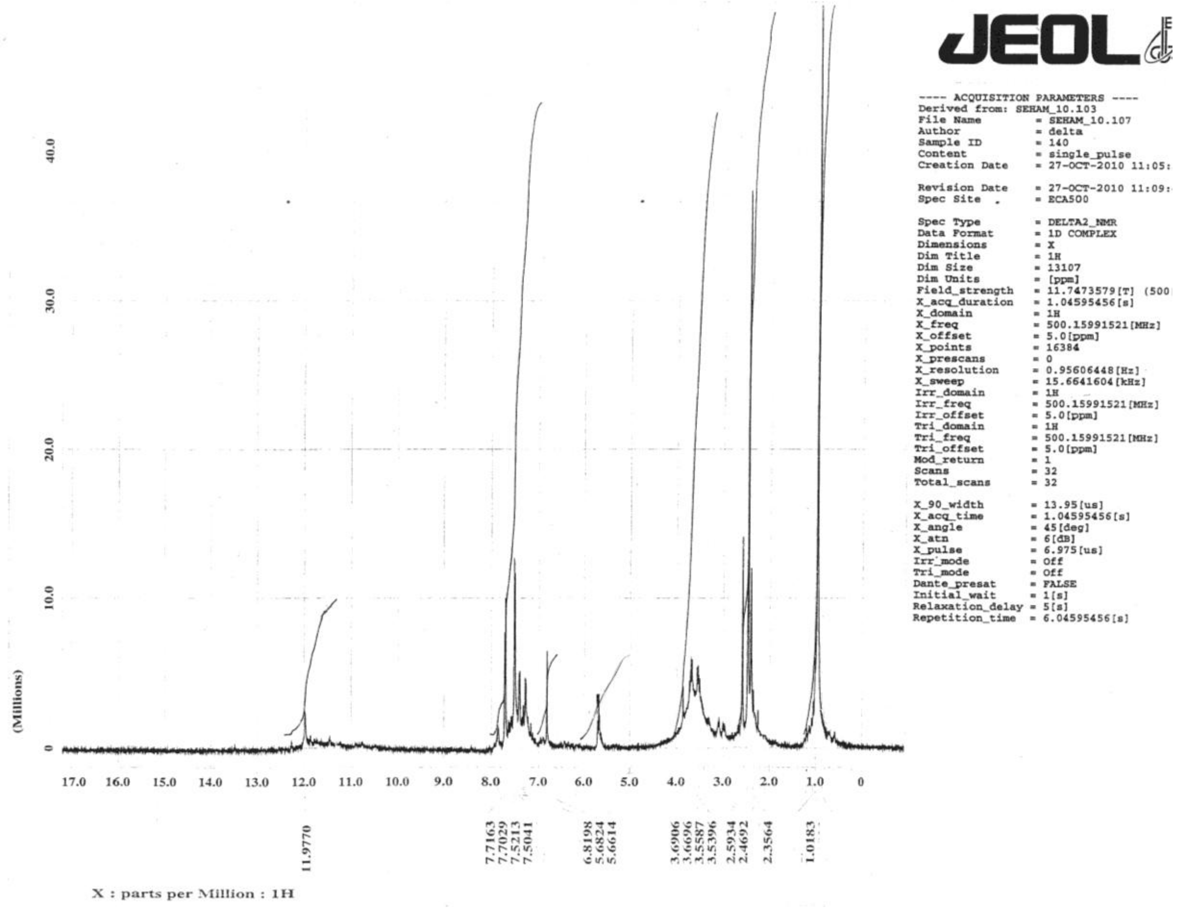

Figure 4. *Cont.*

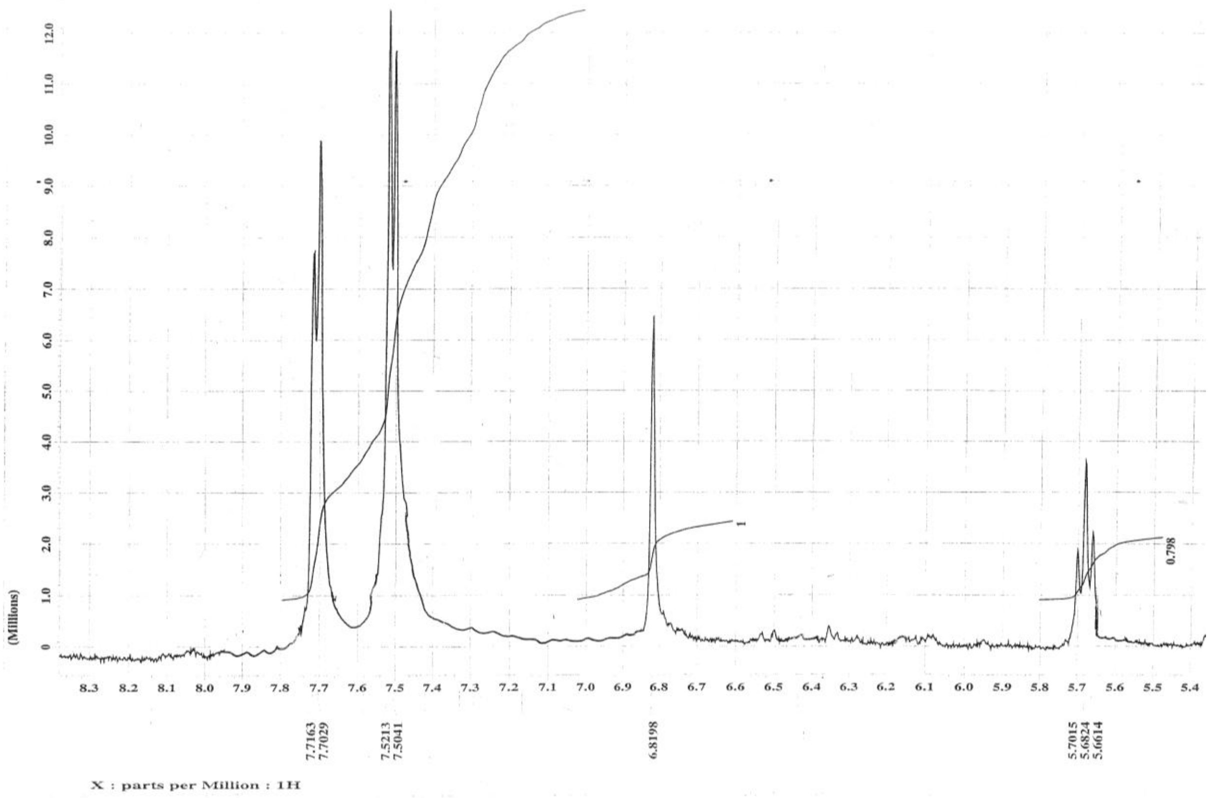

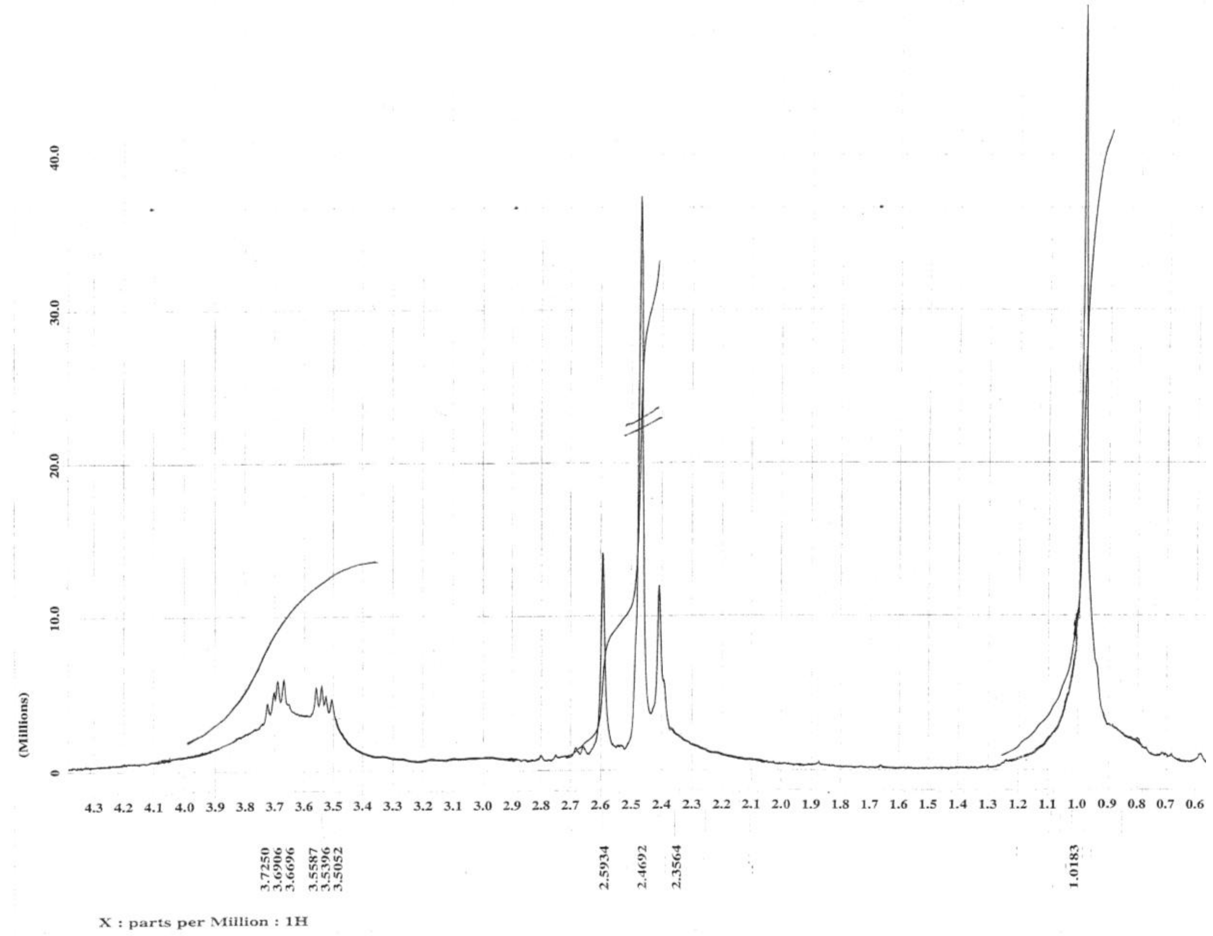

Figure 5. ^{1}H-NMR of compound **18**.

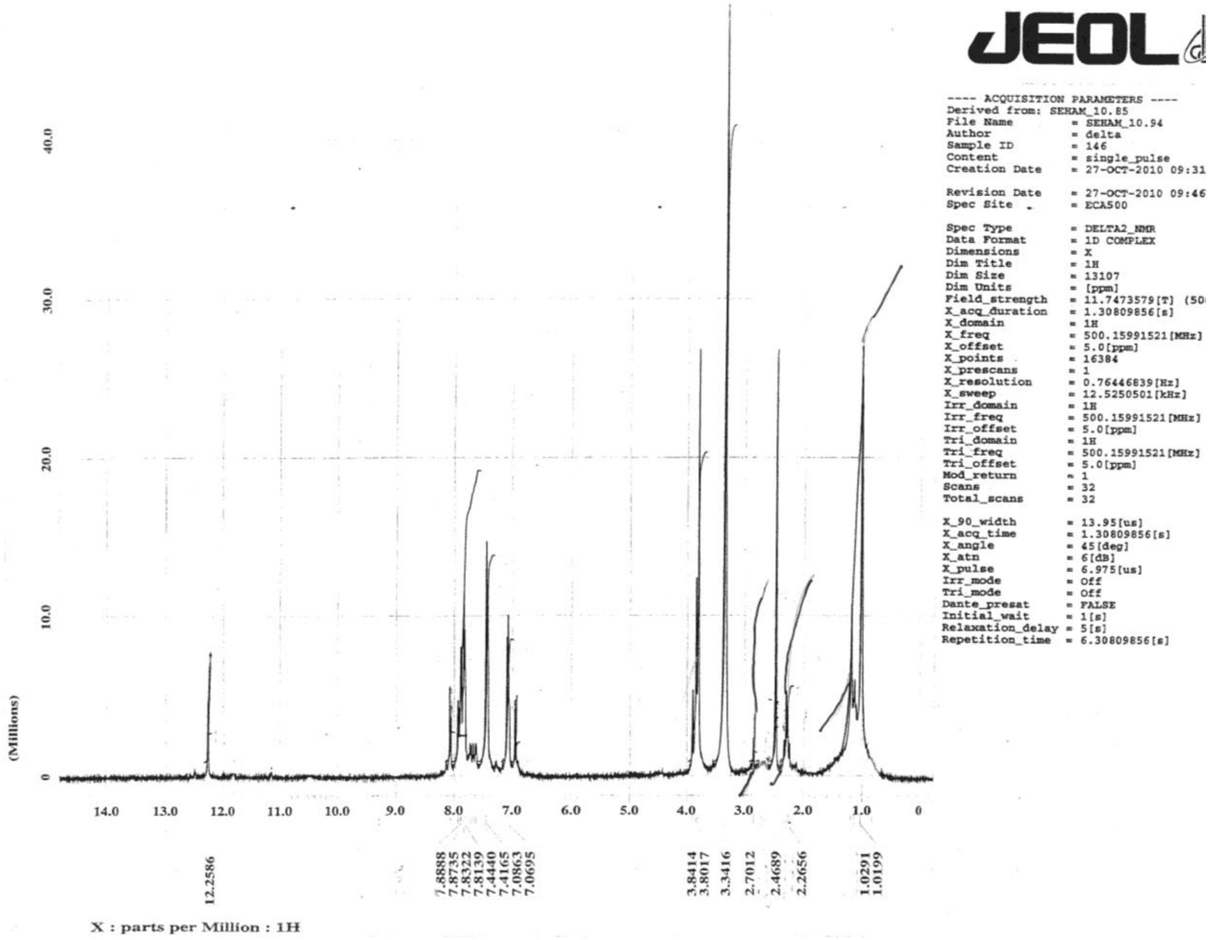

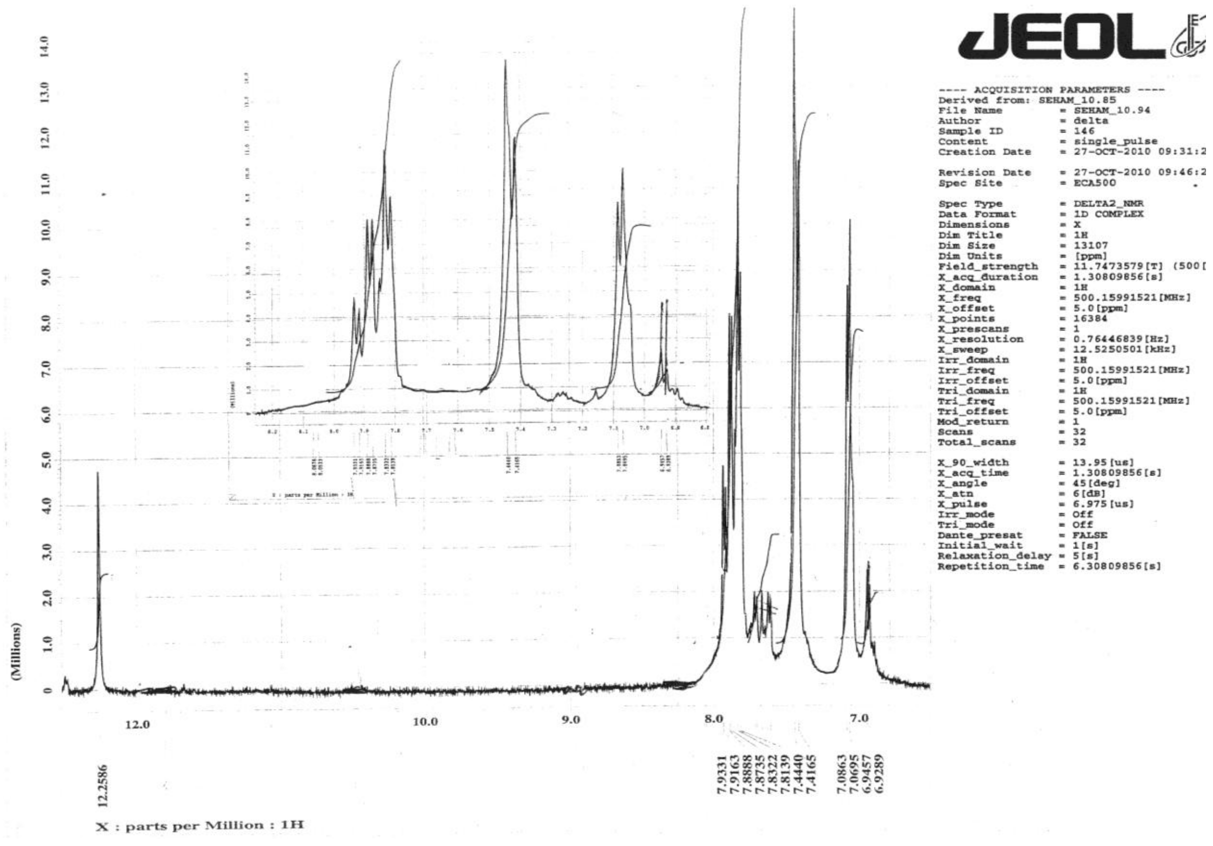

Figure 5. *Cont*.

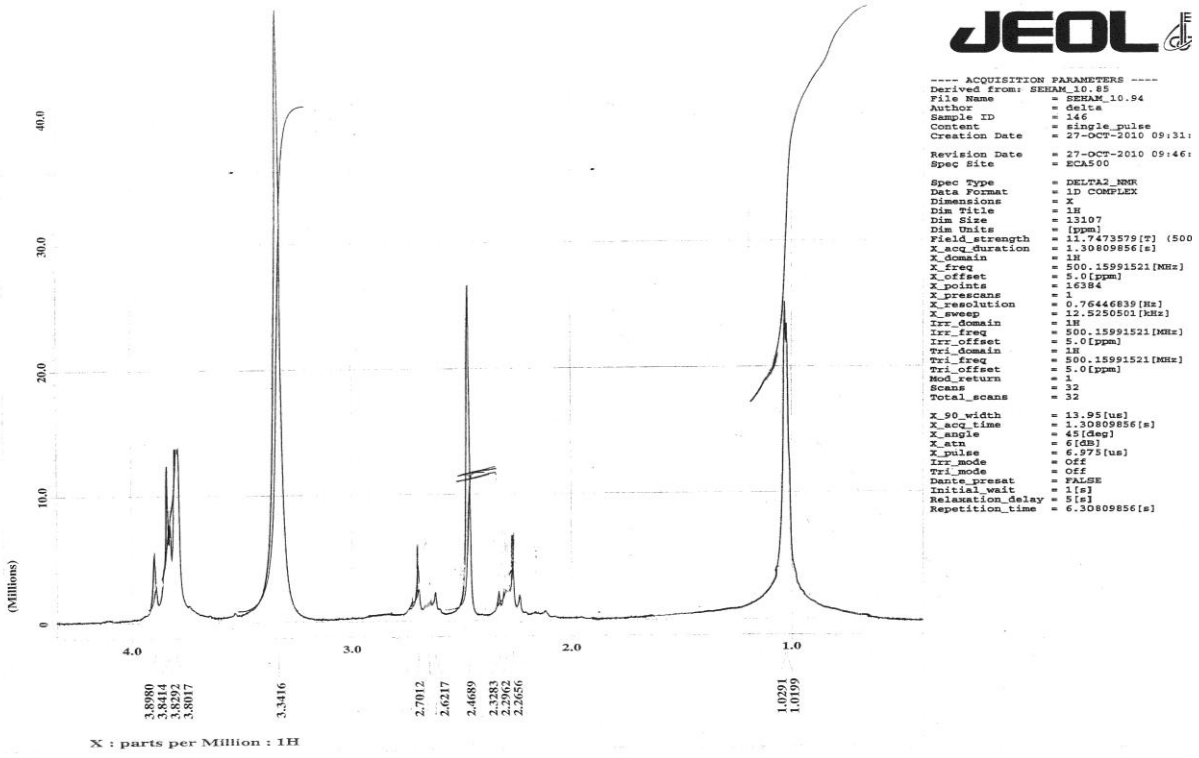

Figure 6. ^{1}H-NMR of compound **22**.

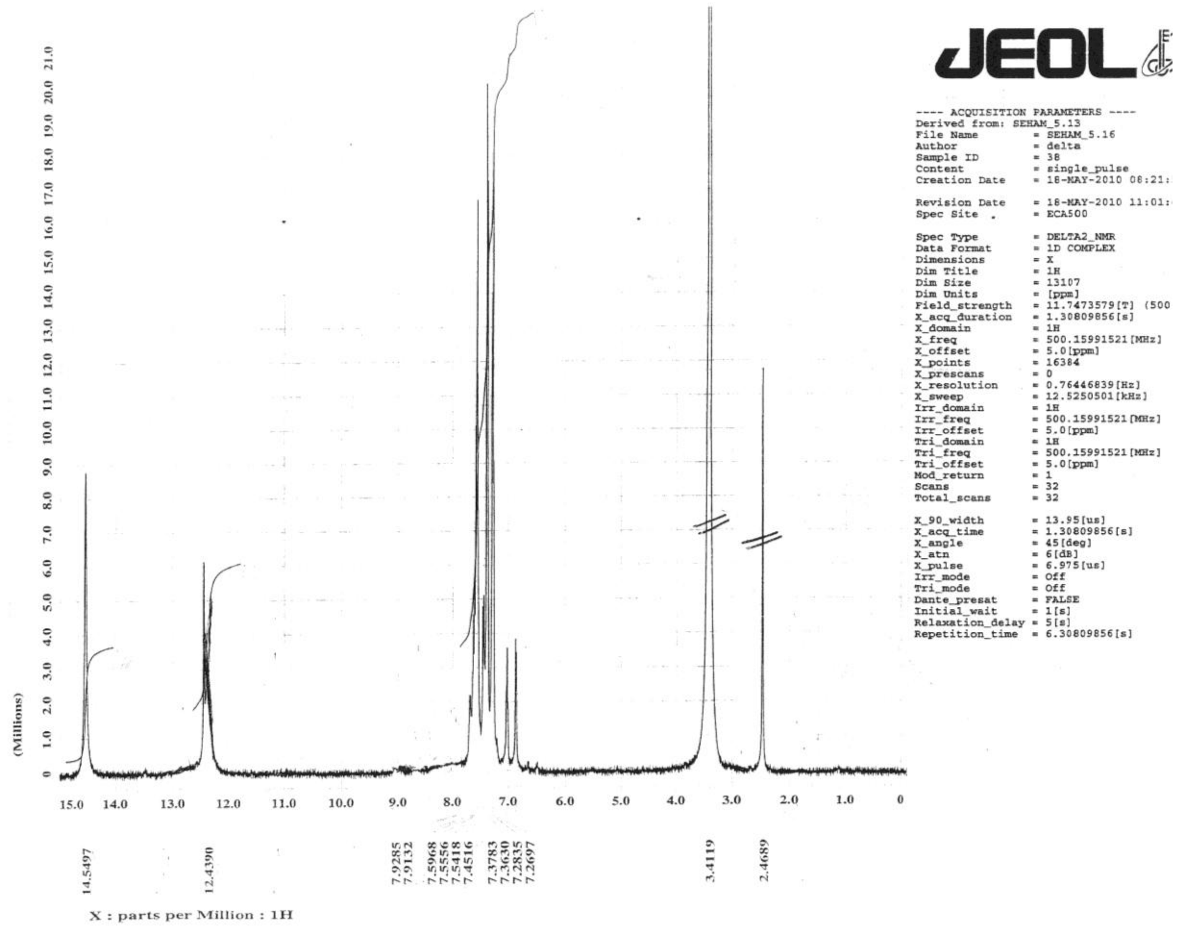

Figure 6. *Cont.*

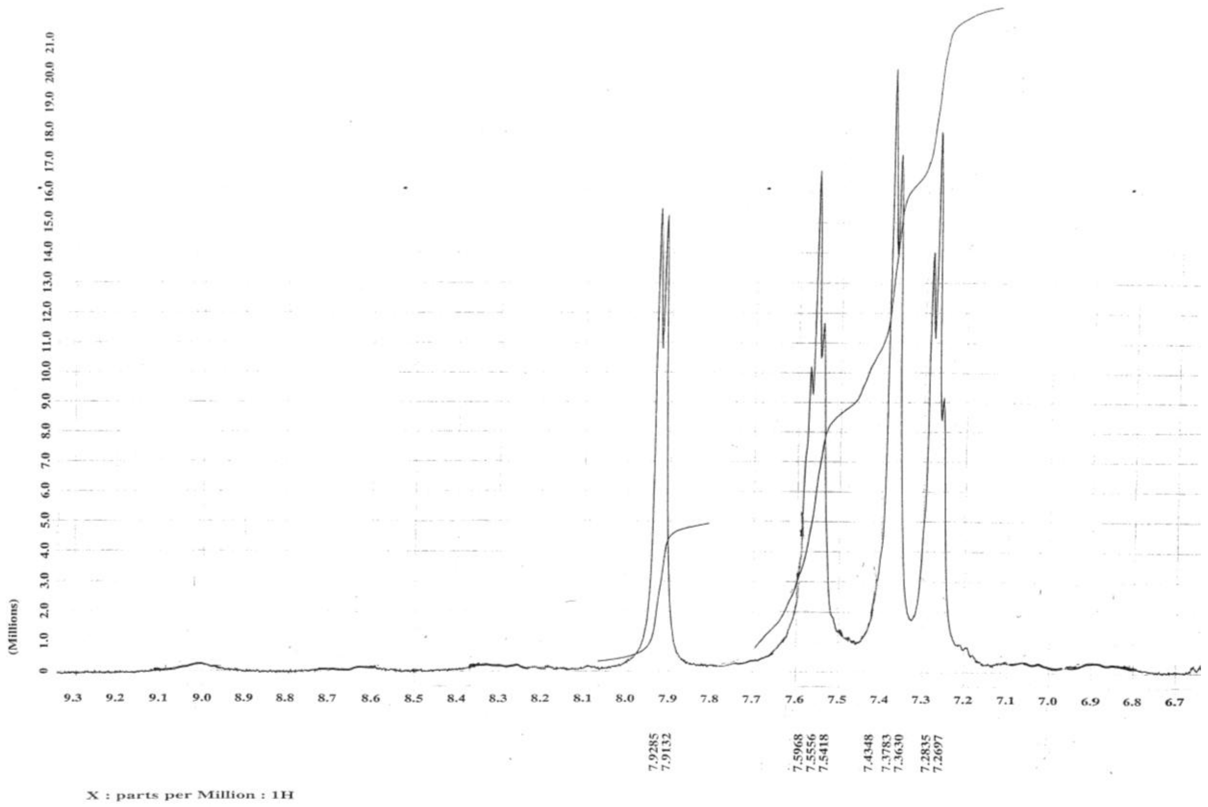

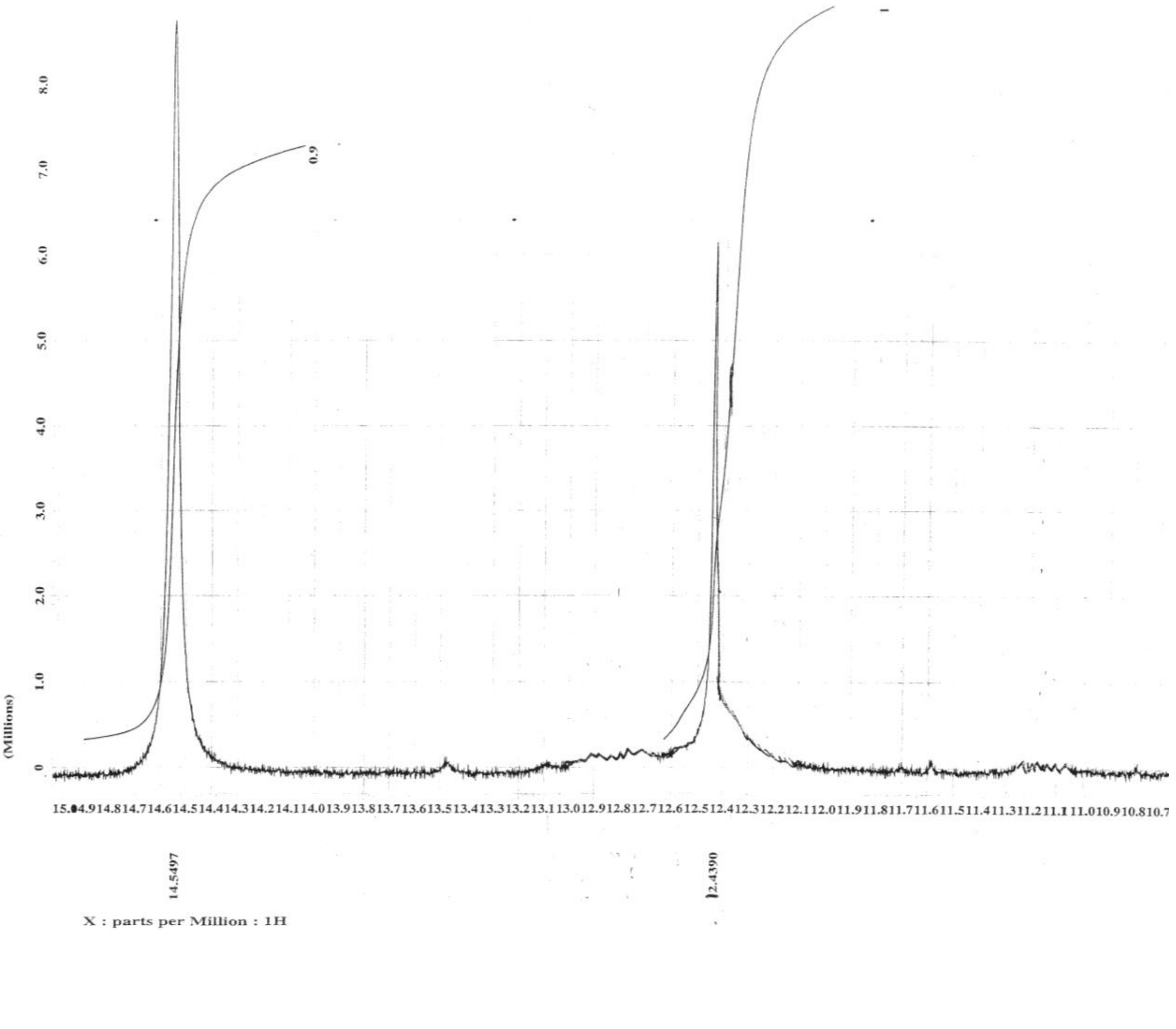

Figure 7. ^{1}H-NMR of compound **24**.

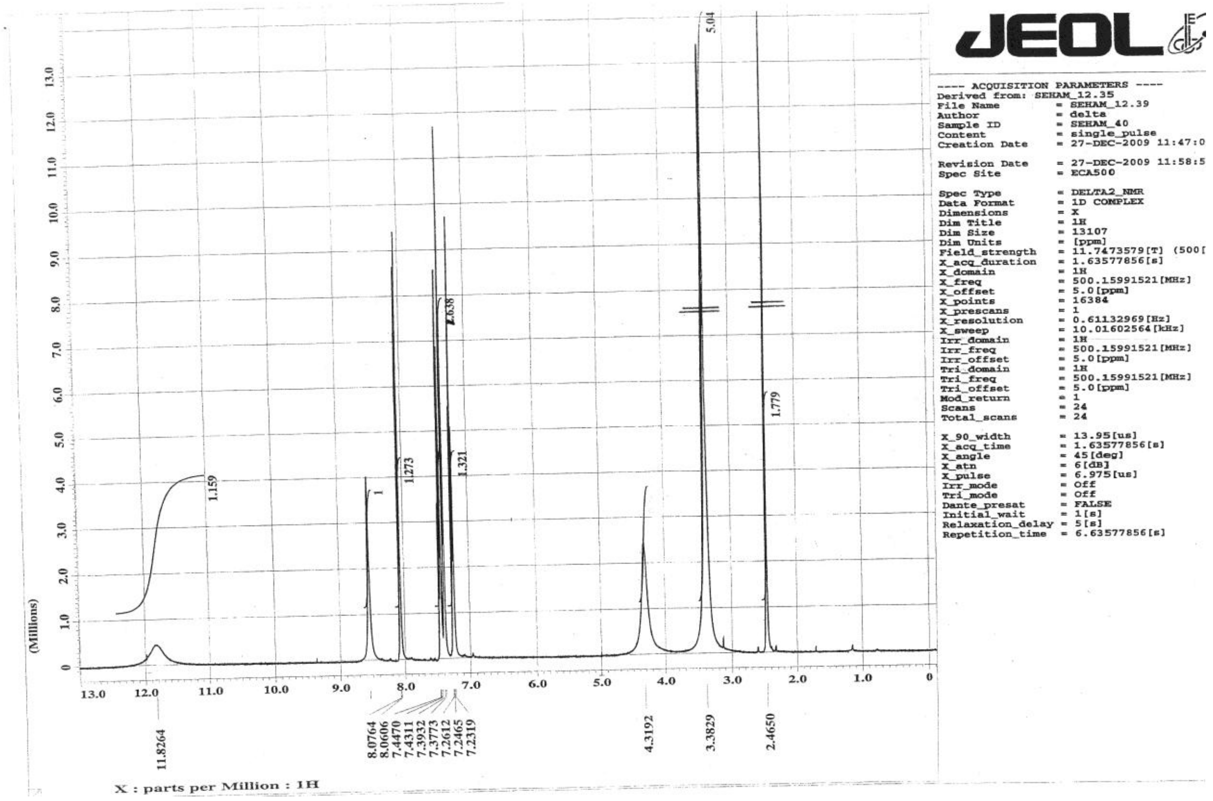

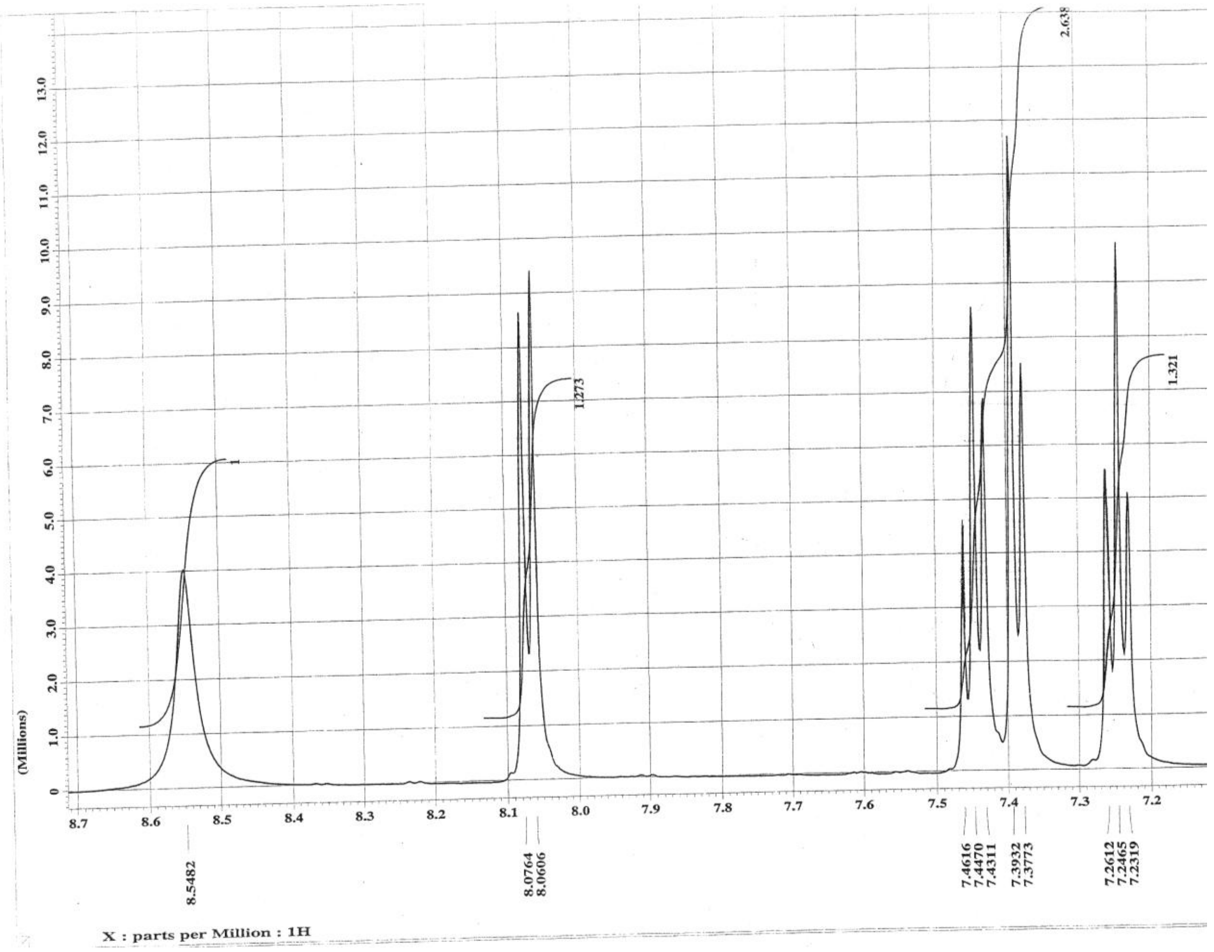

Figure 8. ^{1}H-NMR of compound **27**.

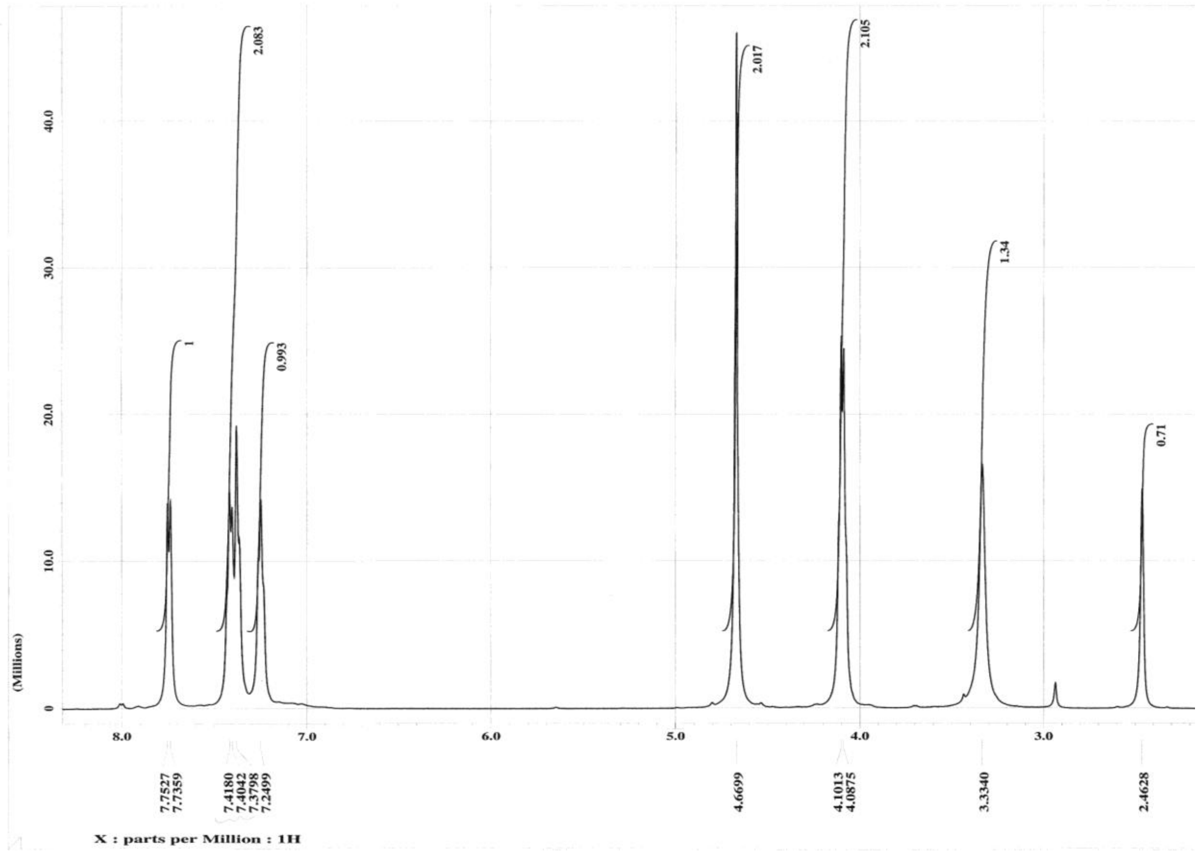

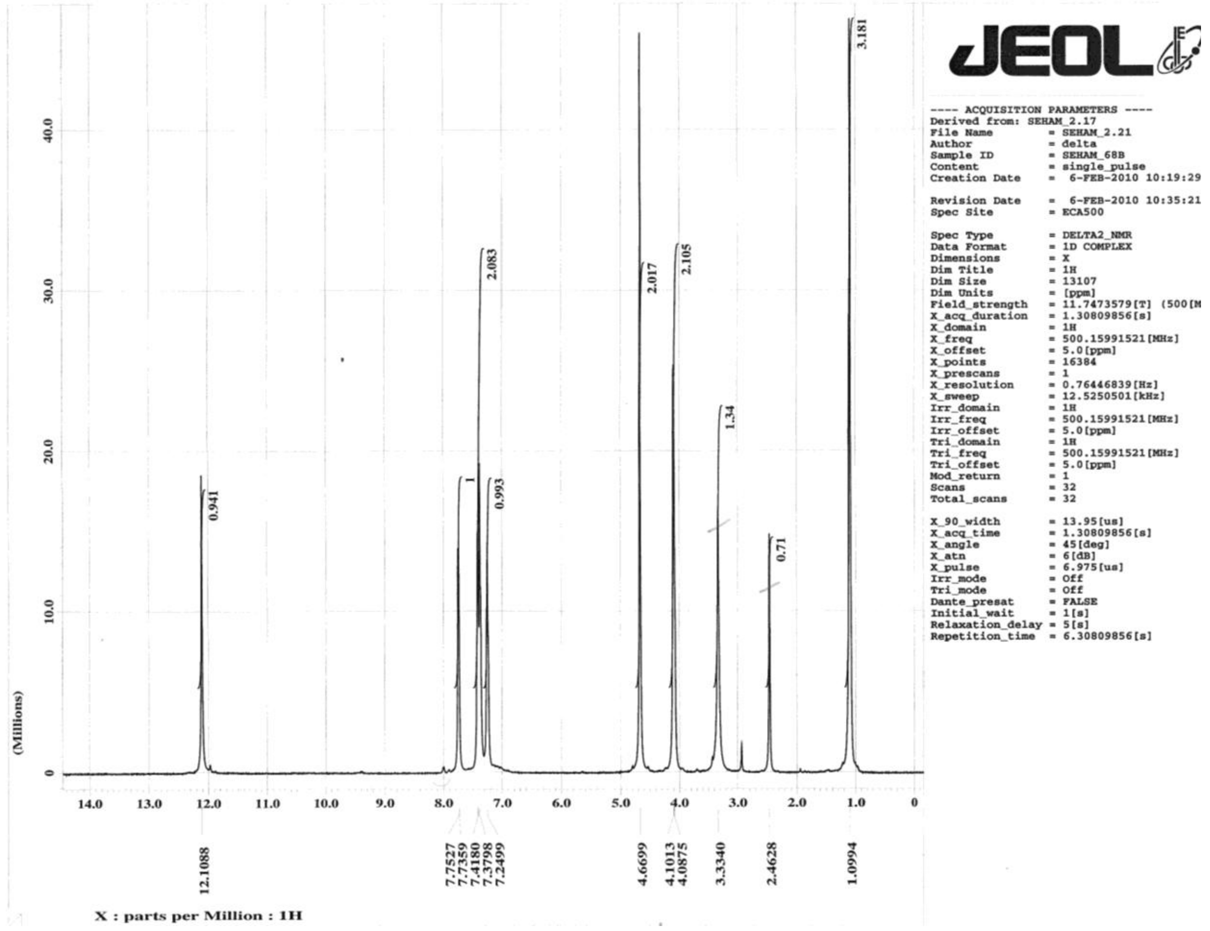

Figure 9. ^{1}H-NMR of compound **33**.

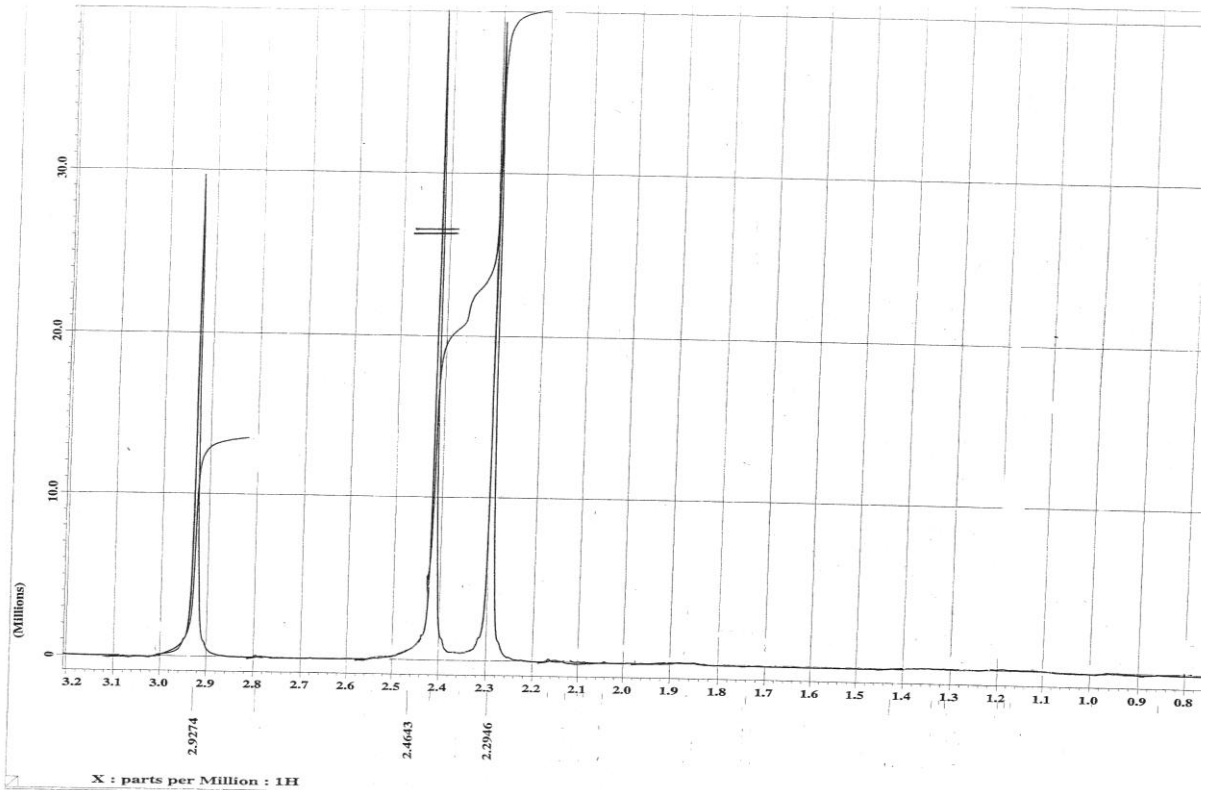

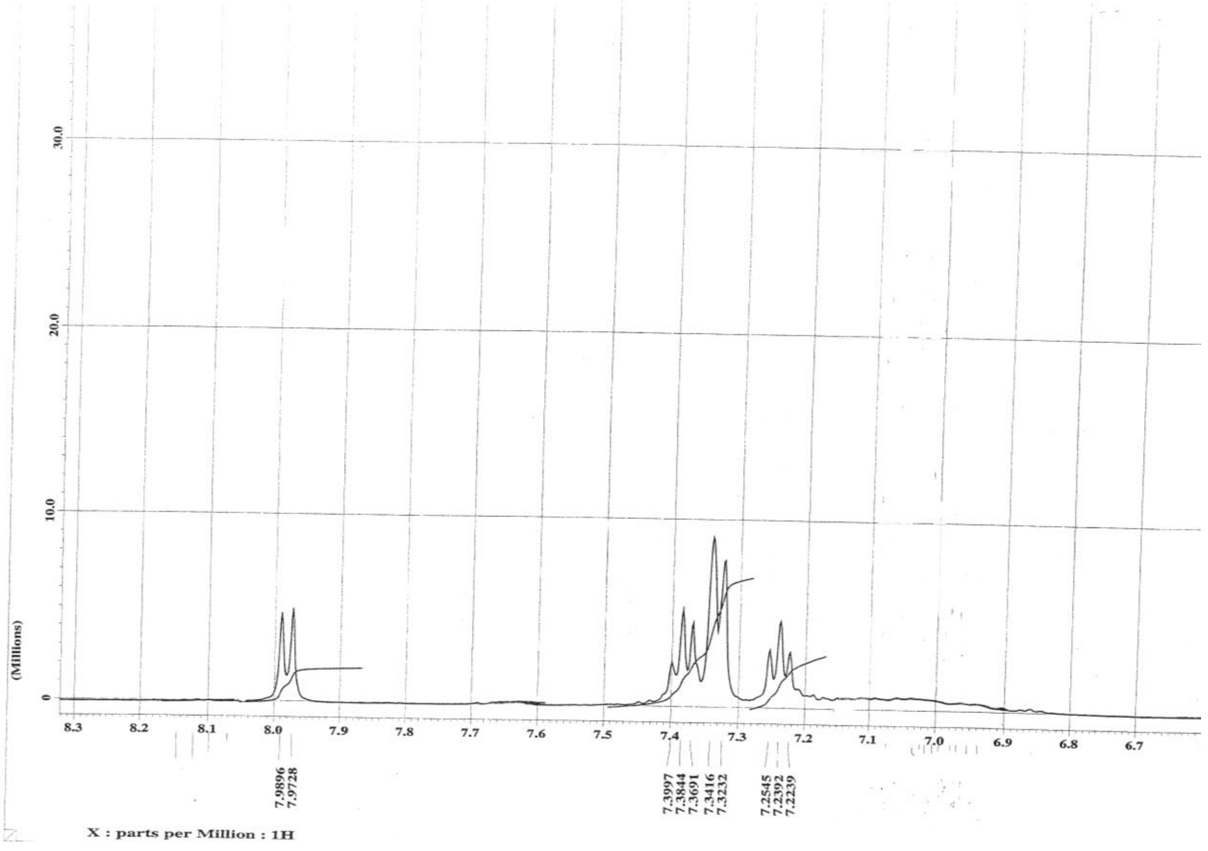

Figure 9. *Cont*.

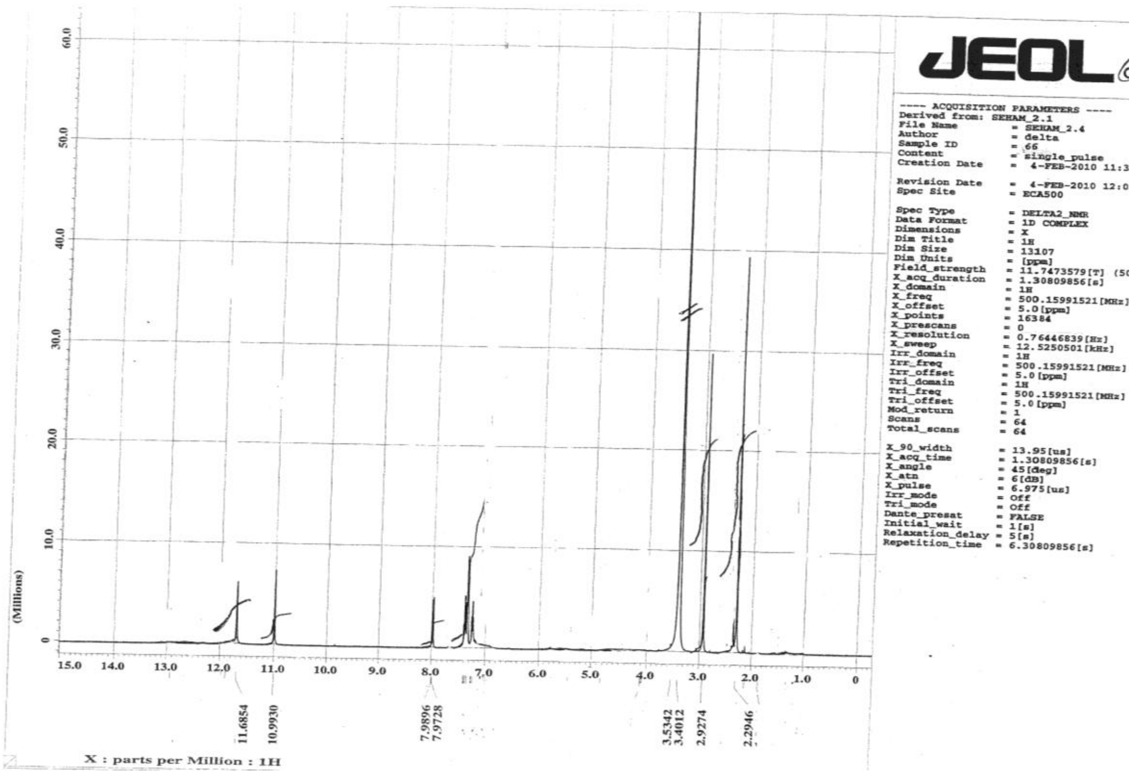

Figure 10. ^{1}H-NMR of compound **34**.

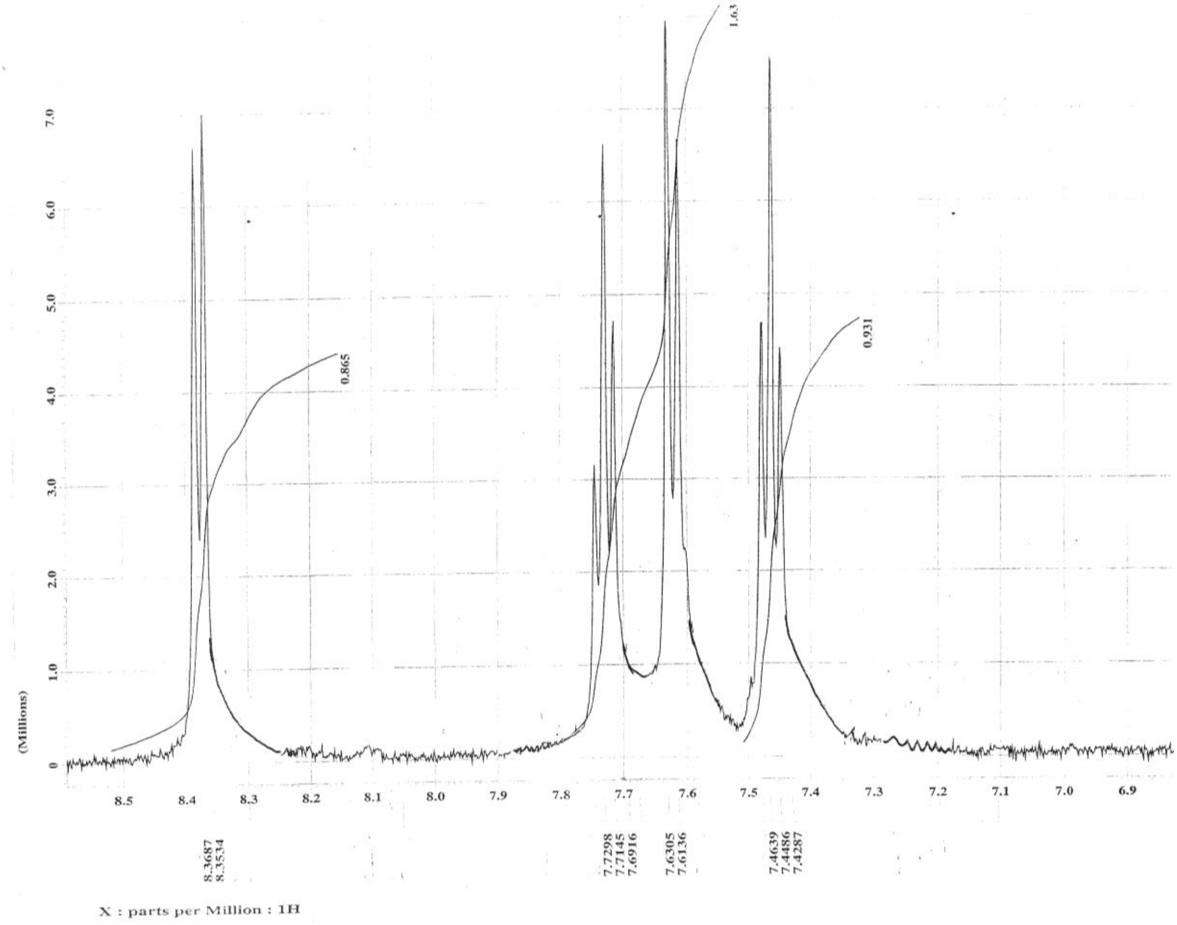

Figure 10. *Cont.*

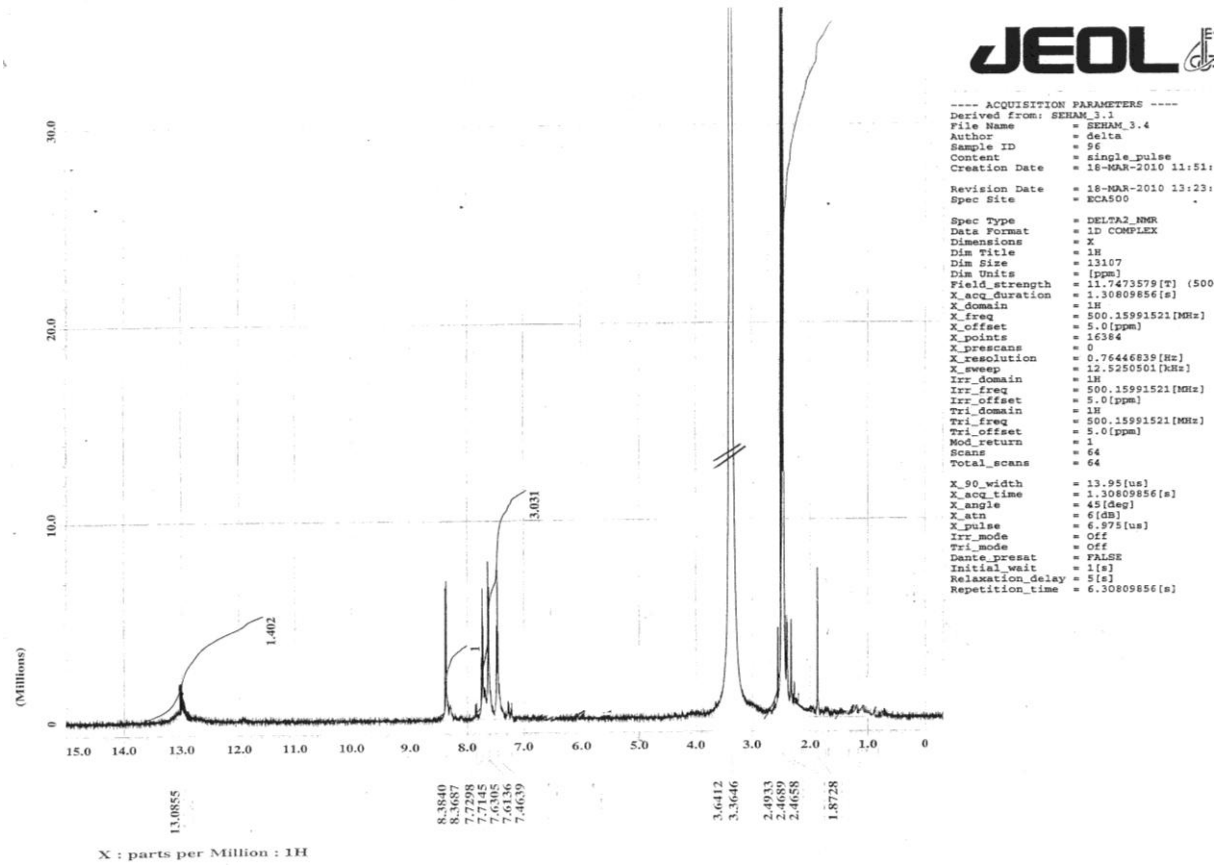

Figure 11. ^{1}H-NMR of compound **36**.

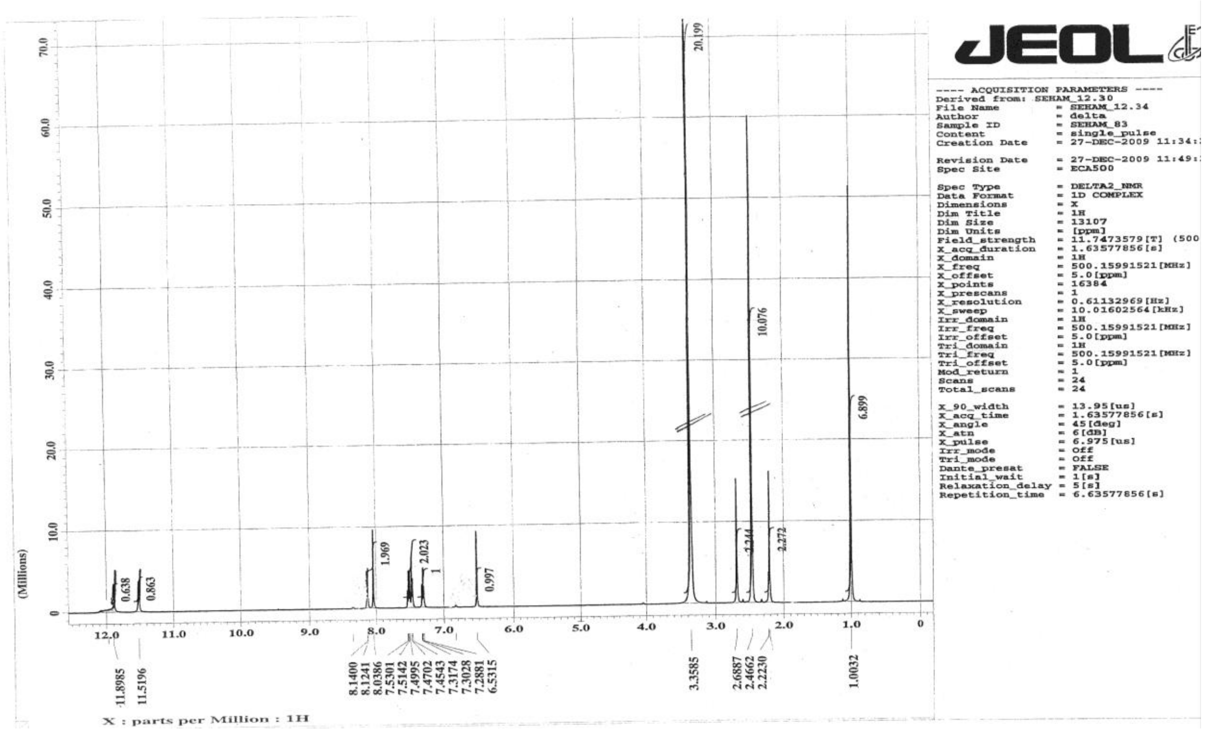

Figure 11. *Cont.*

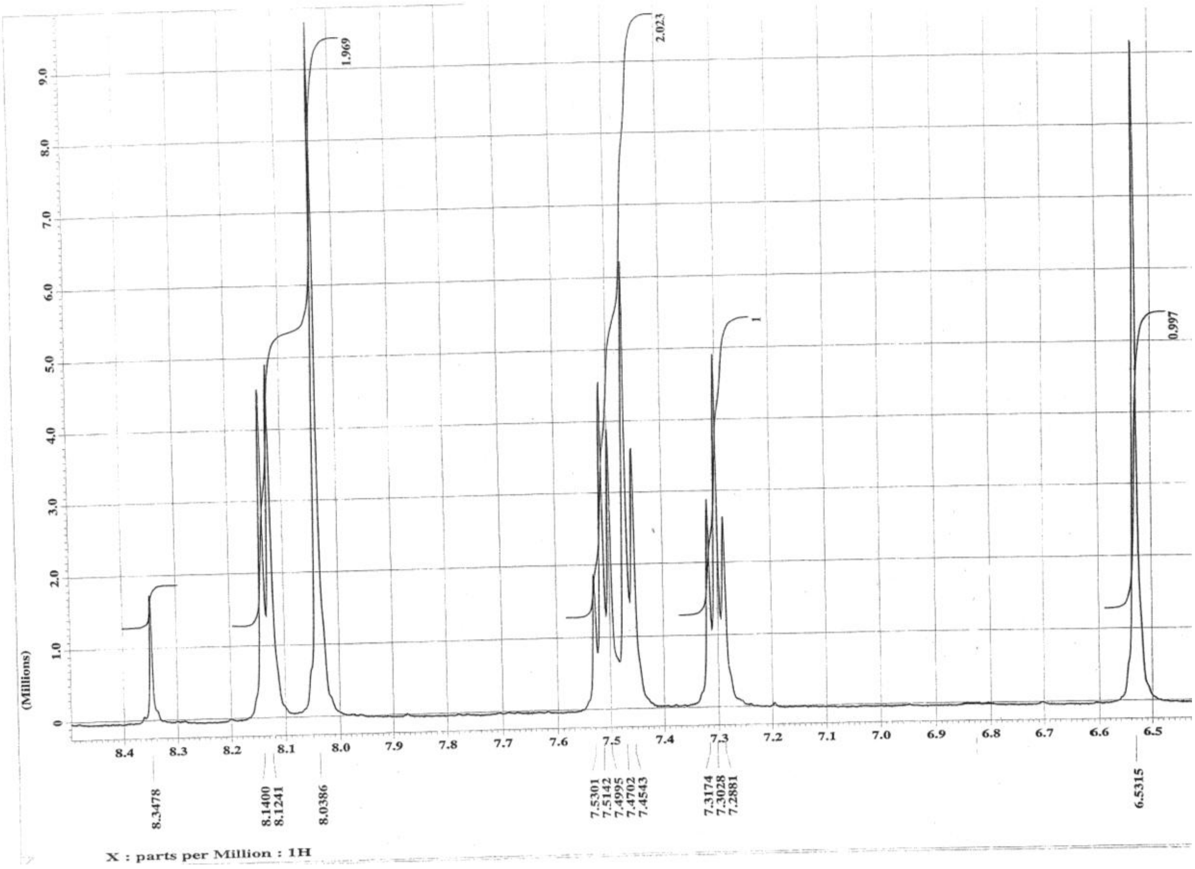

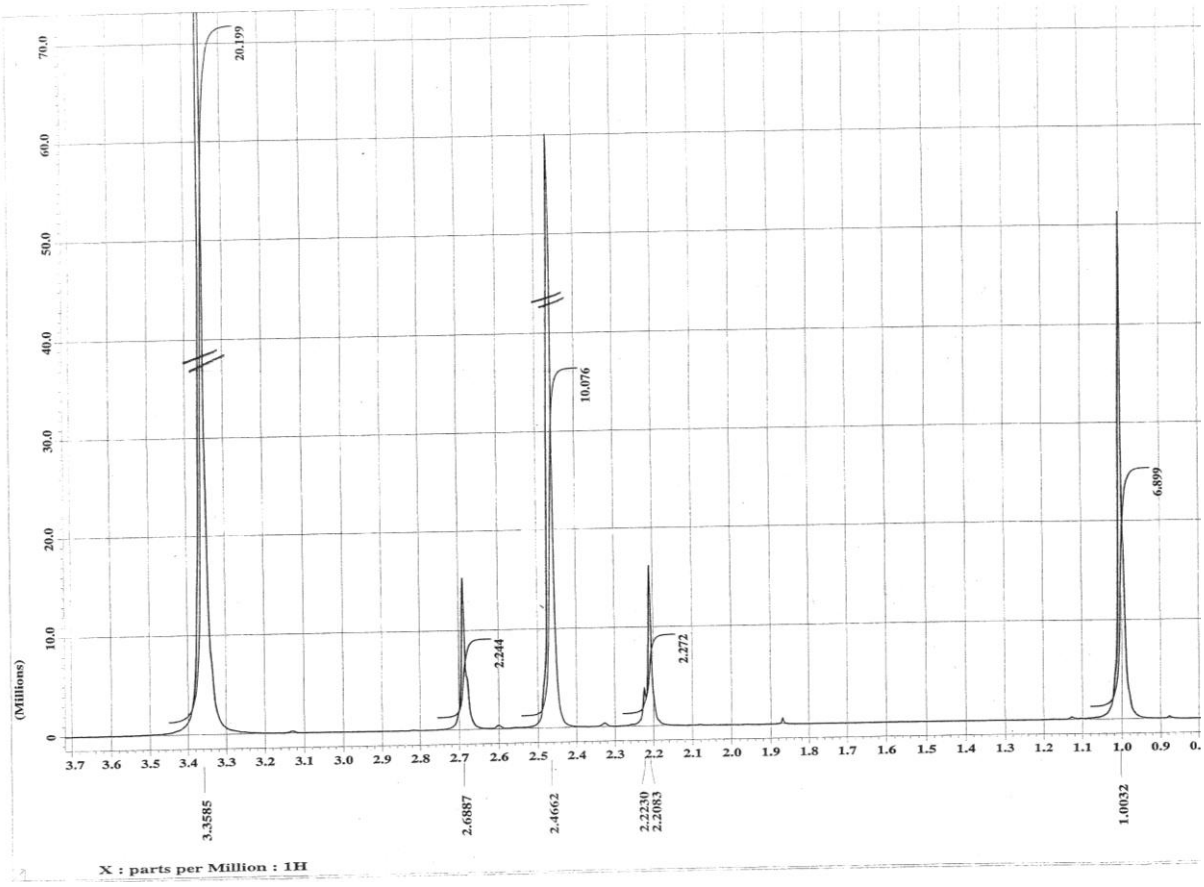

Figure 12. ^{1}H-NMR of compound **38**.

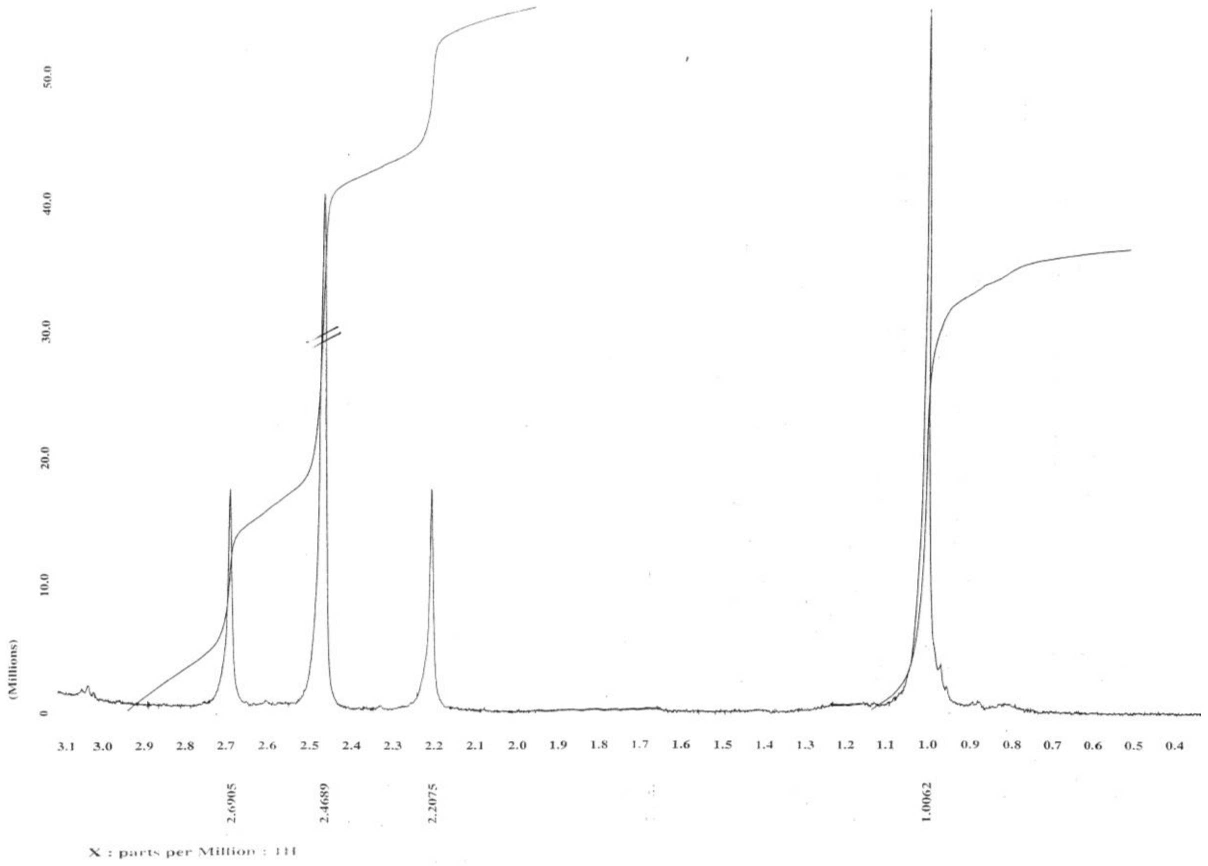

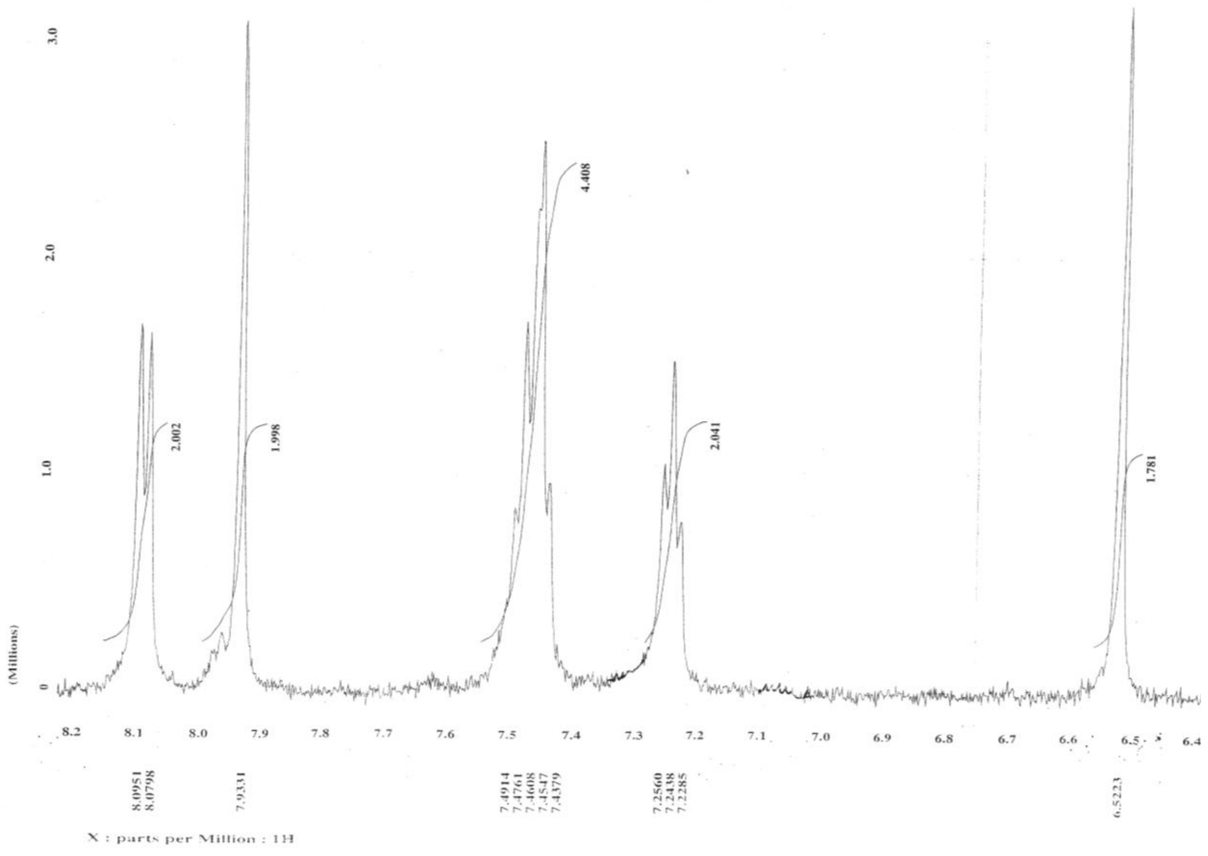

Figure 12. *Cont*.

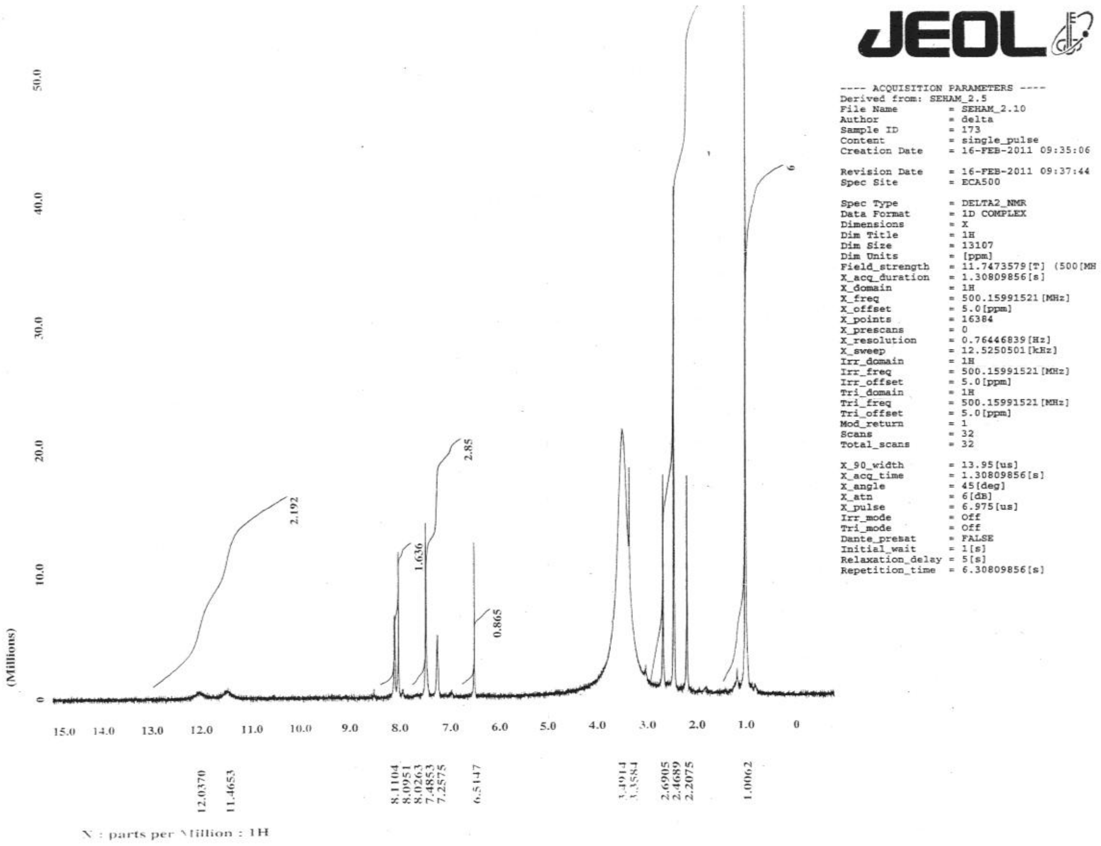

Figure 13. ^{1}H-NMR of compound **40**.

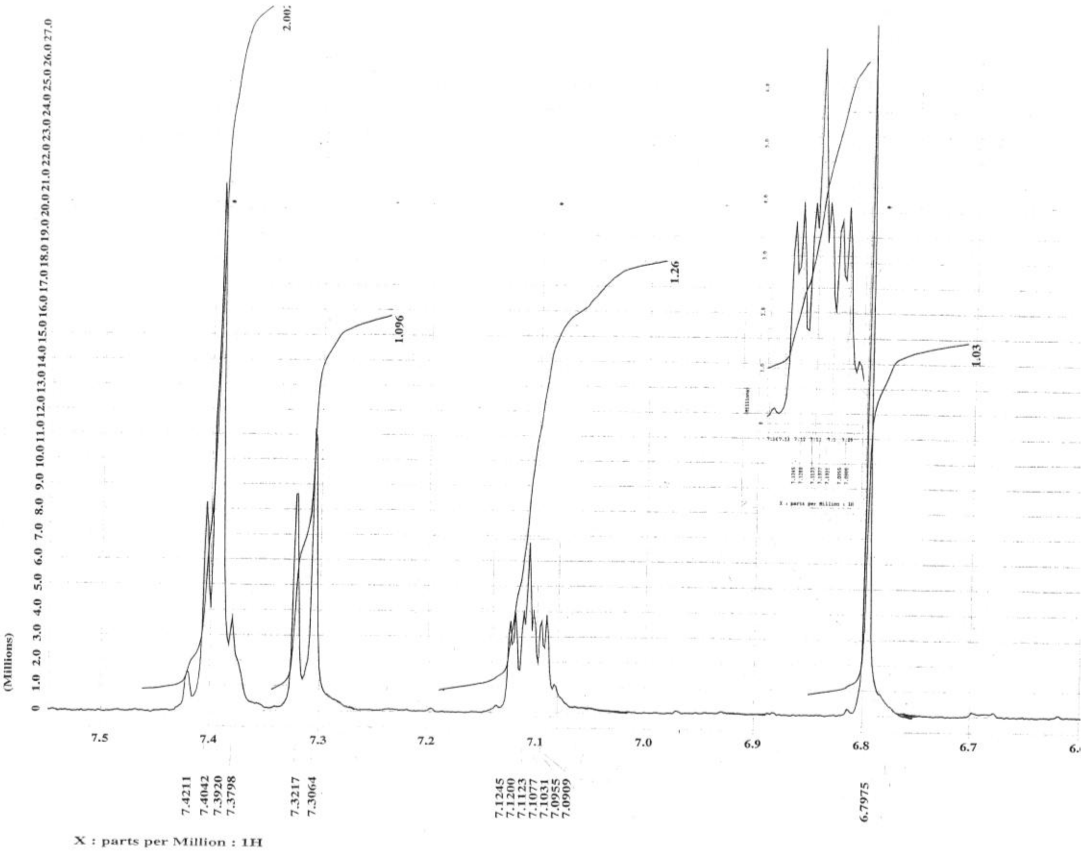

Figure 13. *Cont.*

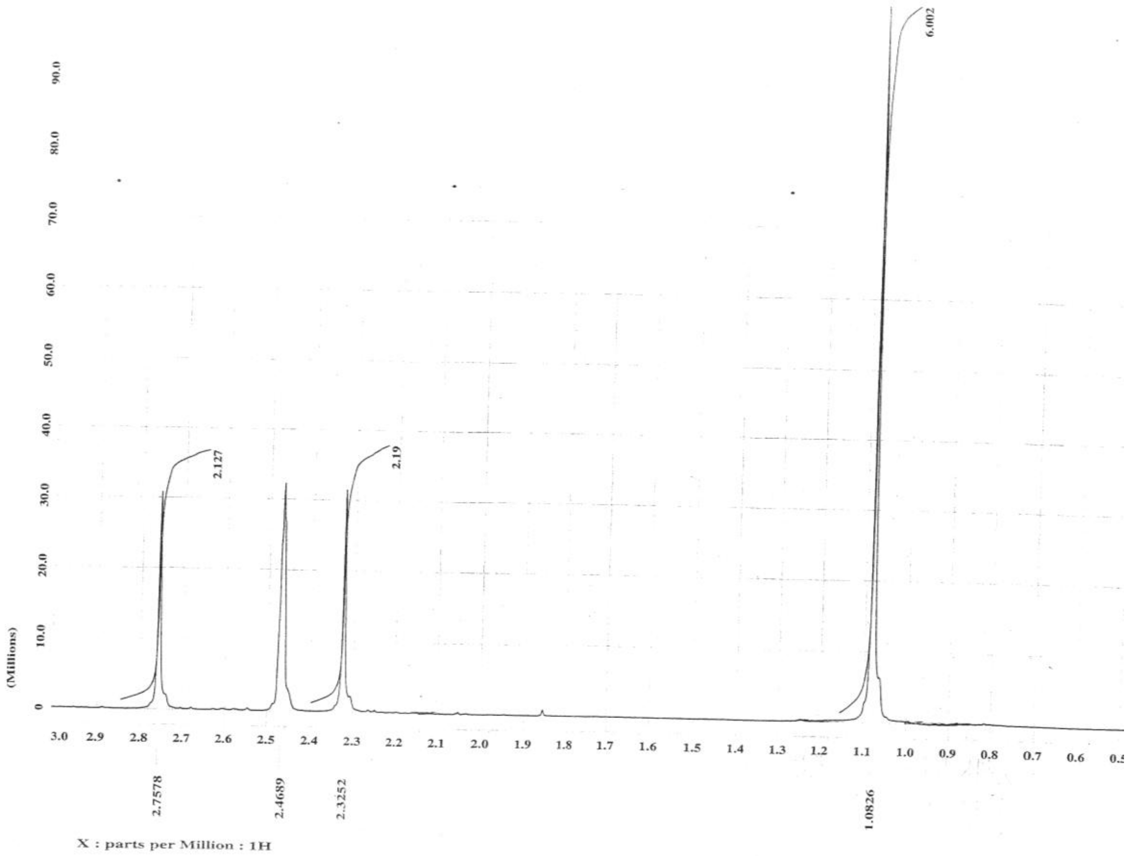

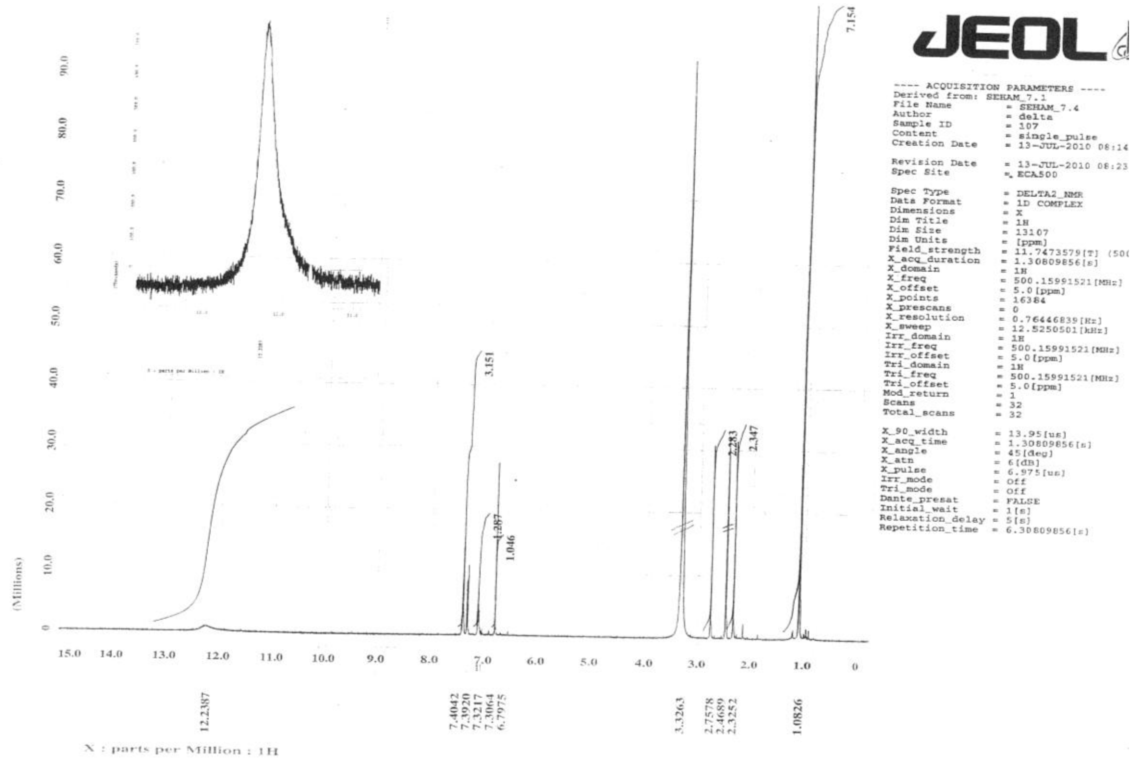

Figure 14. ^{13}C-NMR of compound **2**.

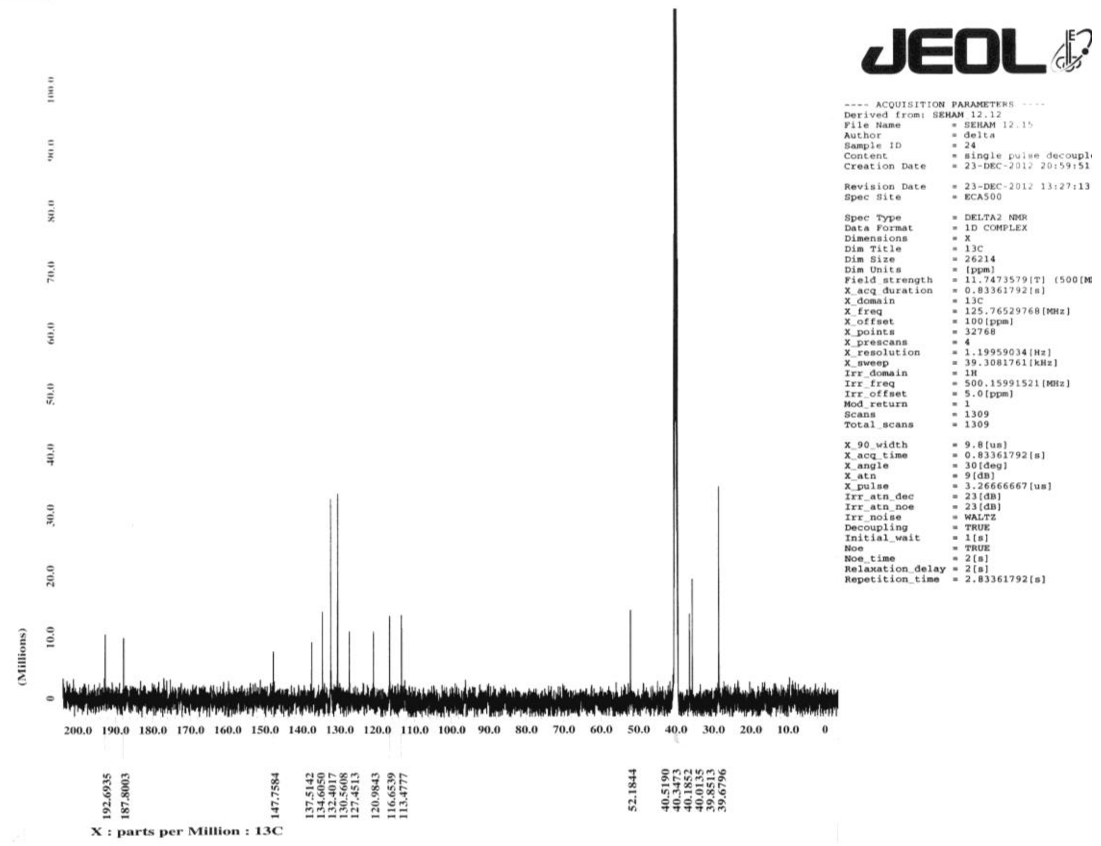

Figure 15. ^{13}C-NMR of compound **27**.

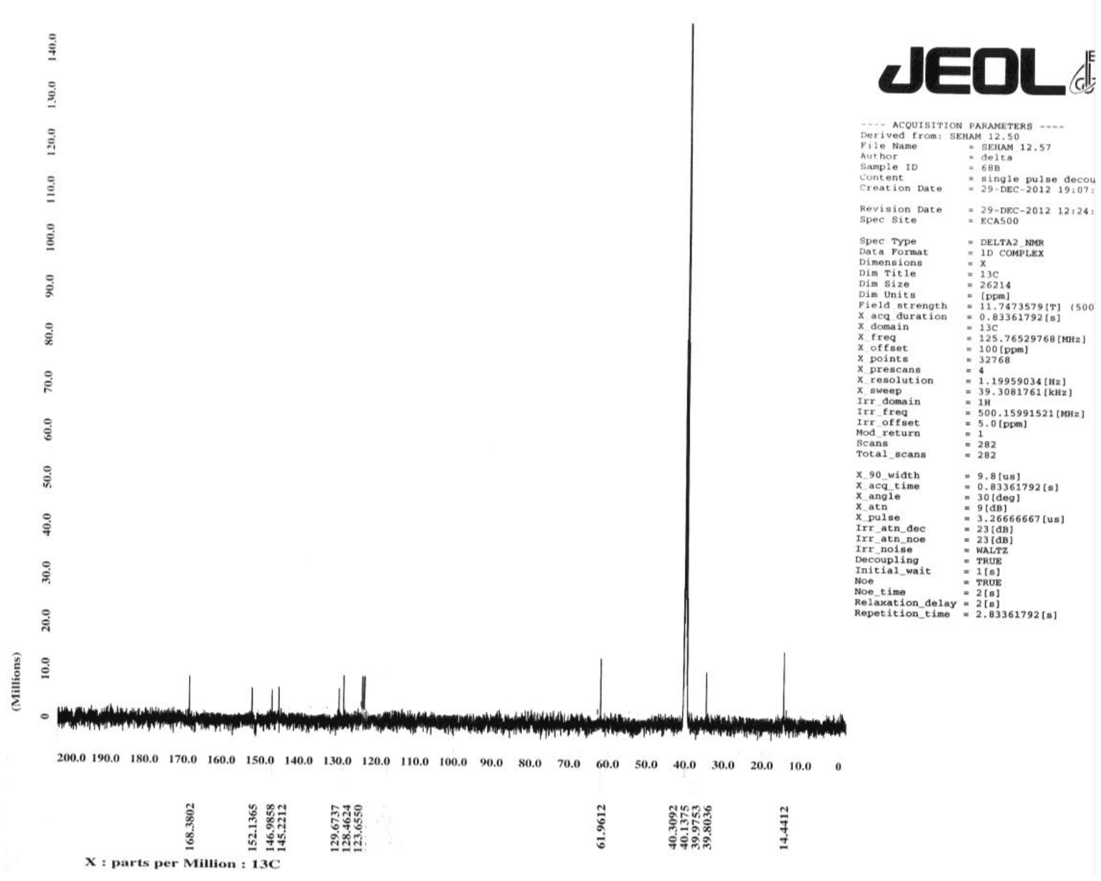

Figure 15. *Cont*.

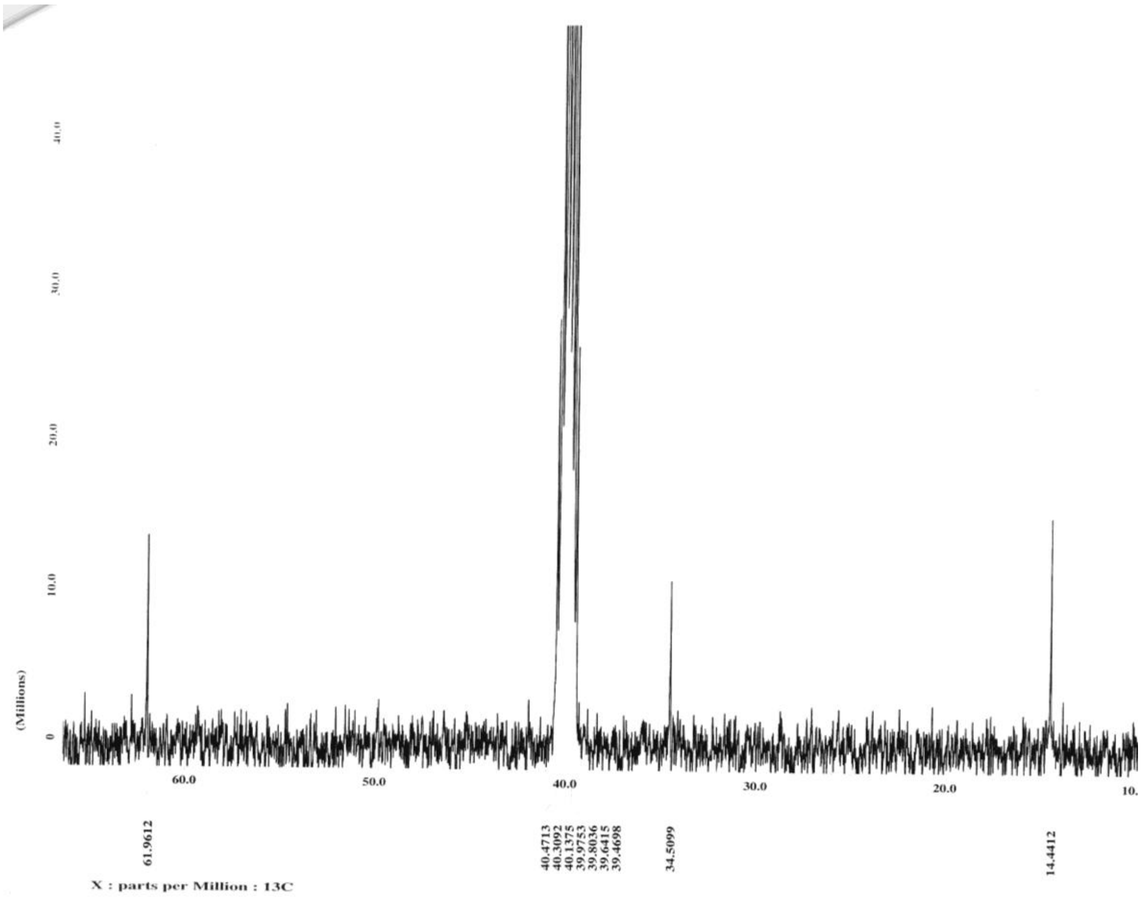

Figure 16. ^{13}C-NMR of compound **35**.

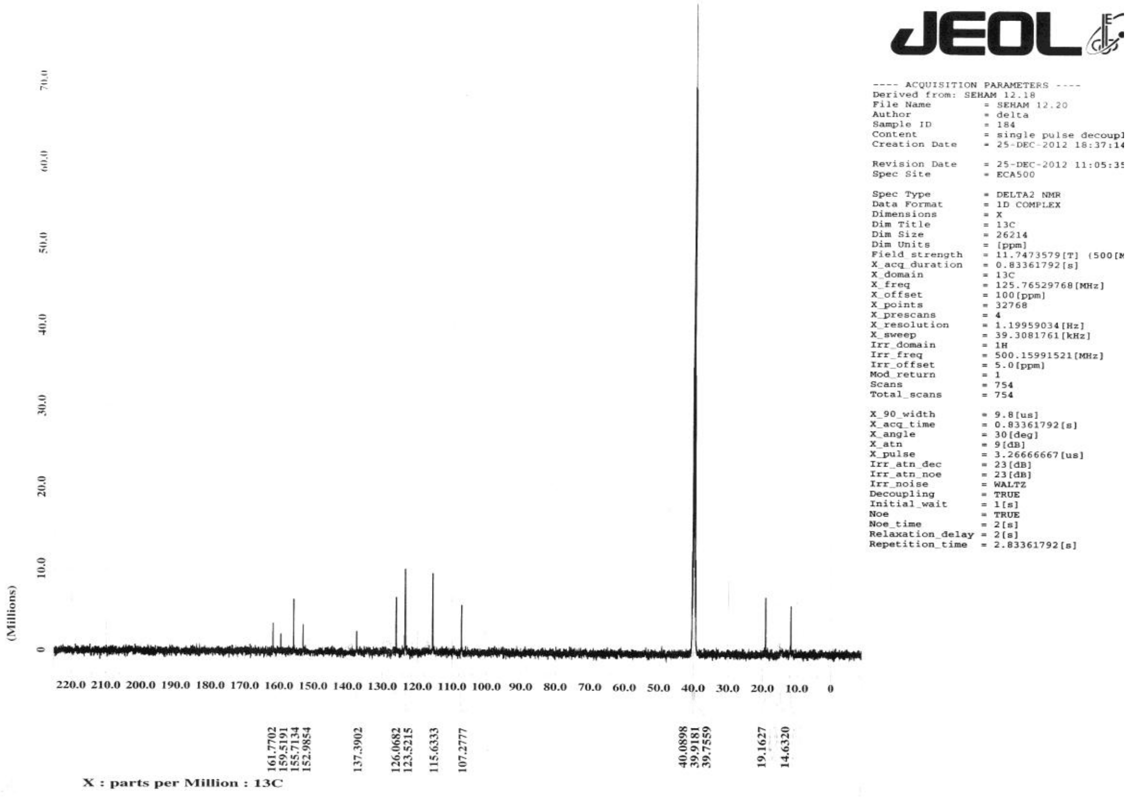

Figure 17. ^{13}C-NMR of compound 36.

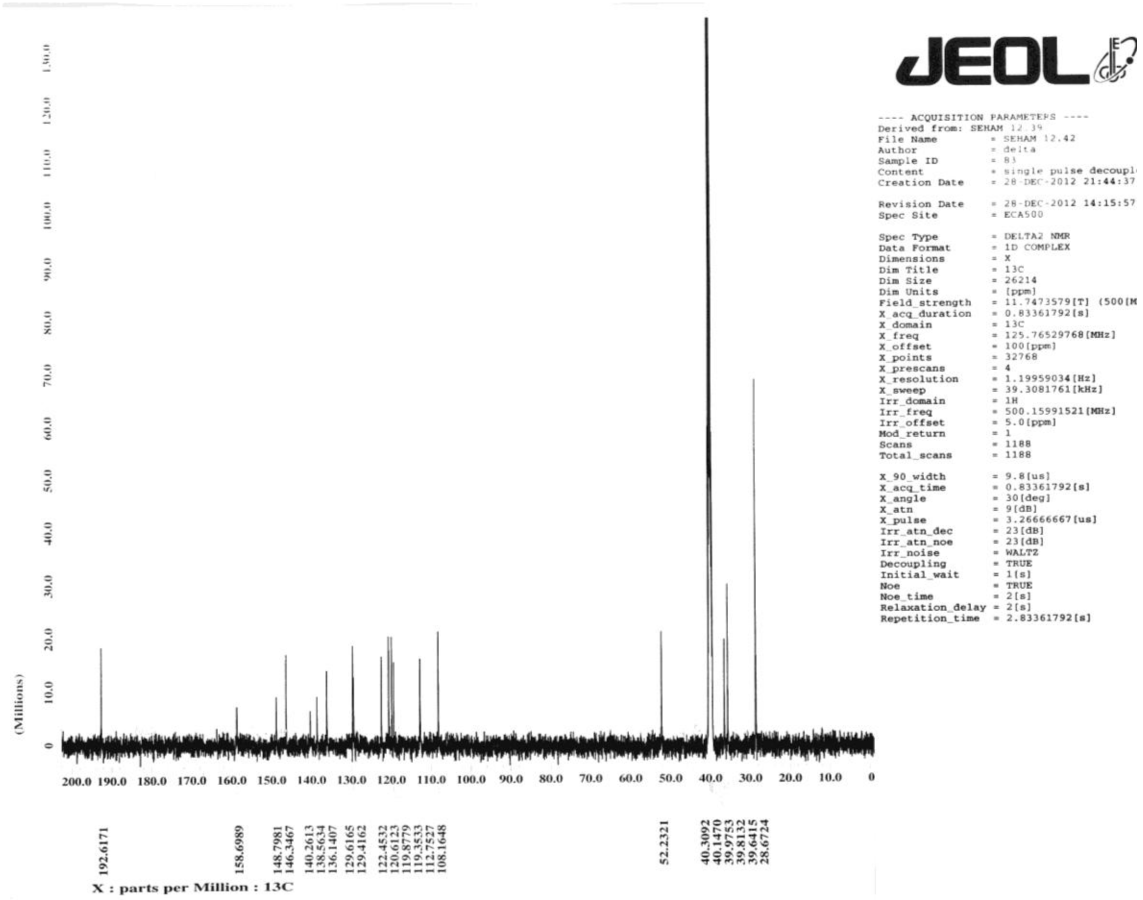

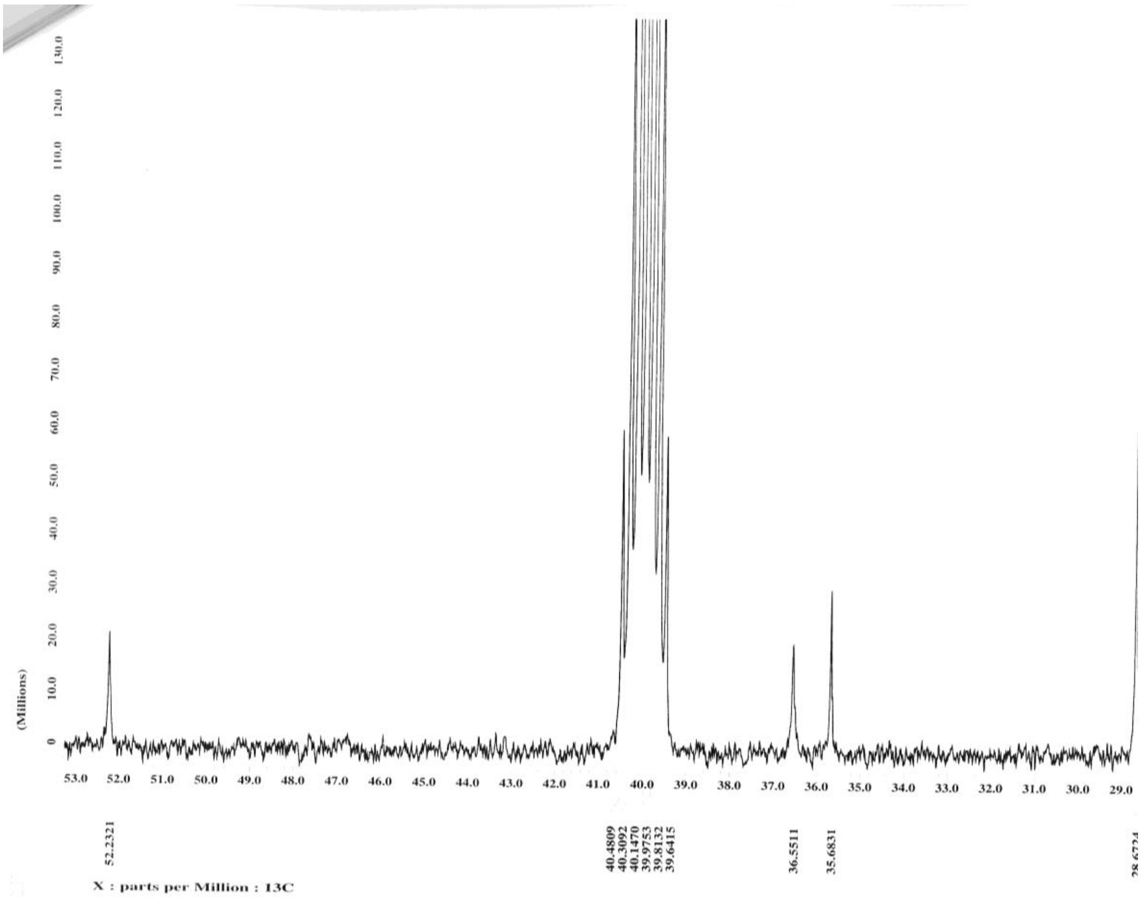

Figure 18. ^{13}C-NMR of compound **22**.

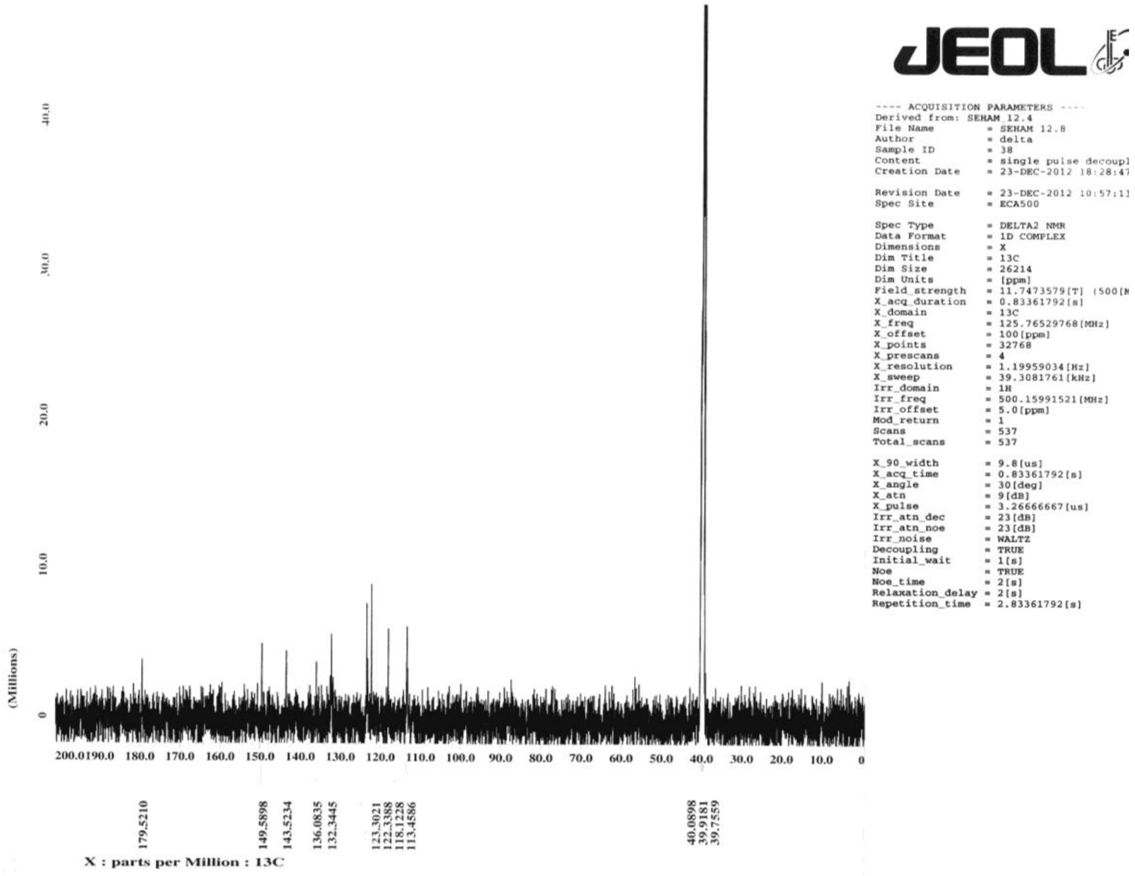

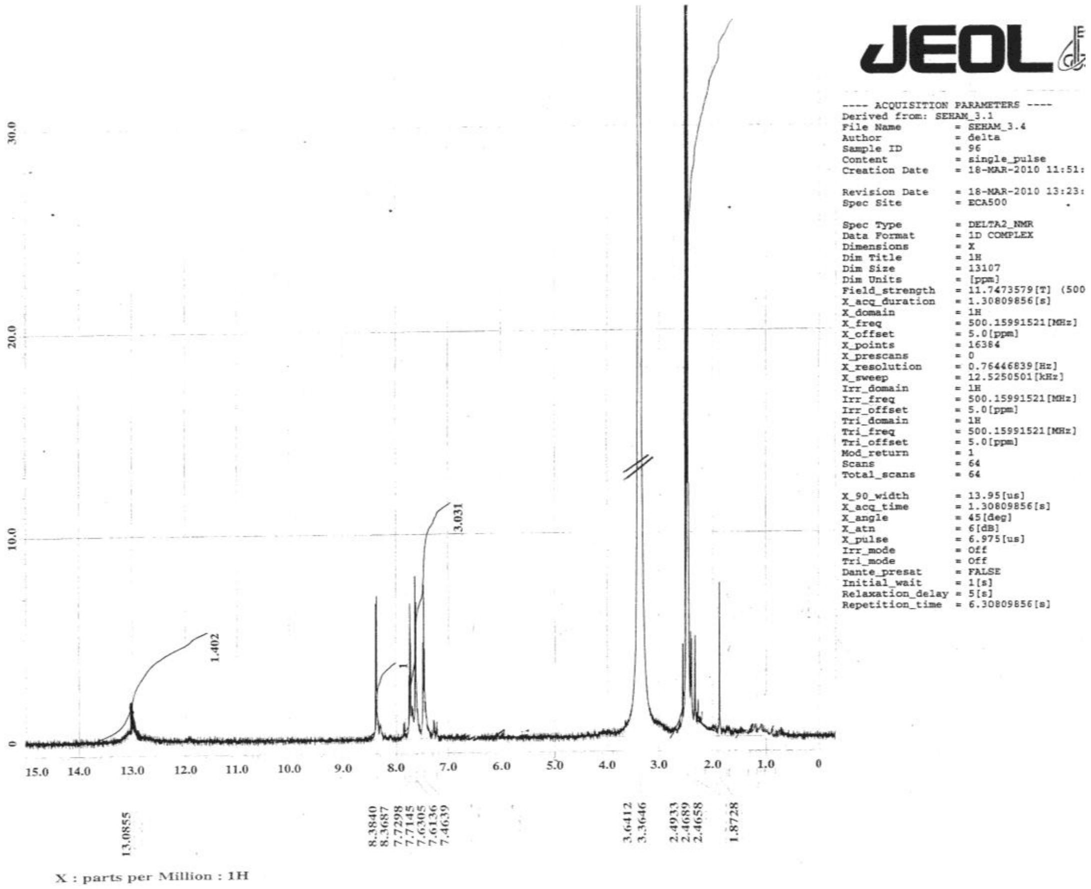

Supplement: Supplementary file 1 [file molecules-18-02683-s001.pdf]
